# Photocatalyst for Visible-Light-Driven Sm(II)-Mediated Reductions

**DOI:** 10.1021/acs.orglett.4c03723

**Published:** 2024-12-11

**Authors:** Monika Tomar, Caroline Bosch, Jules Everaert, Rohan Bhimpuria, Anders Thapper, Andreas Orthaber, K. Eszter Borbas

**Affiliations:** Department of Chemistry, Ångström Laboratory, Uppsala University, Box 523, 75120 Uppsala, Sweden

## Abstract

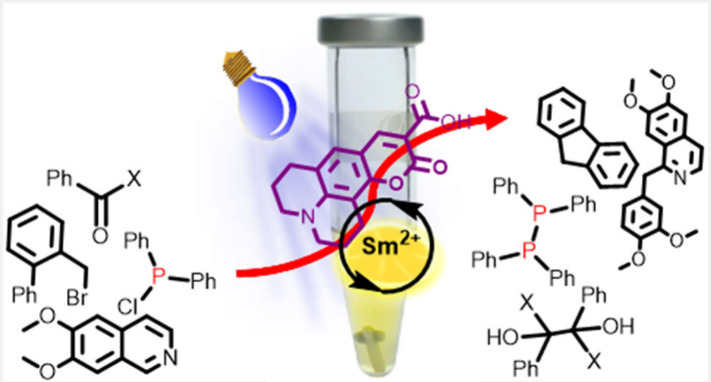

Commercially available coumarin 343 in combination with reducible Sm(III) ions catalyzed divalent lanthanide-mediated C=O, C–halogen, P–Cl, and N=N reductions at ambient temperature in aqueous solvent mixtures. The catalyst absorbs visible light efficiently. The active divalent species is formed by photoinduced electron transfer from coumarin 343 to the stable trivalent precursor, and the coumarin could be regenerated by strictly 1 equiv of ascorbic acid.

The role of lanthanides (Ln) in catalysis has long been restricted to Lewis acid catalysis.^1^ It is only recently that cycling between stable Ln(III) and a less stable Ln(IV) or Ln(II) state to promote a redox reaction by catalytic amounts of a lanthanide has become possible. Ce(III) photocatalysts can mediate dehalogenations, C–H activations, borylations, and C–C bond formations.^2^ Ln(II) compounds are versatile one-electron reductants that can affect functional group interconversions and C–C and C–X bond formations with high selectivity even in complex substrates.^3^ SmI_2_, the most common Ln(II) reagent, is typically used in large excess, which makes such reactions environmentally burdensome. Approaches toward Ln(II) catalysis include chemical^4^ or electrochemical^5^ Ln(II) generation or the reliance on radical propagation.^6^ These attempts suffer from a limited substrate scope, diminished selectivity, or the need for toxic additives.

Ln(III) can be reduced by excited-state chromophores.^7^ The chromophore can be regenerated by a reductant, closing the catalytic cycle.^8,9^ The catalysts in Figure 1 could reduce benzyl and aryl halides and nitro, C=O, and P=O groups, and the reductions could initiate C–C and C–N bond formations in yields comparable to the analogous stoichiometric reactions. While promising, **LnL1**, **LnL2**, and **LnL3** had limitations: weak or no visible absorption, the need for Zn or *N*,*N*-diisopropylethylamine (DIPEA) sacrificial reductants, and multistep ligand syntheses in the case of **LnL1** and **LnL2**.

**Figure 1:**
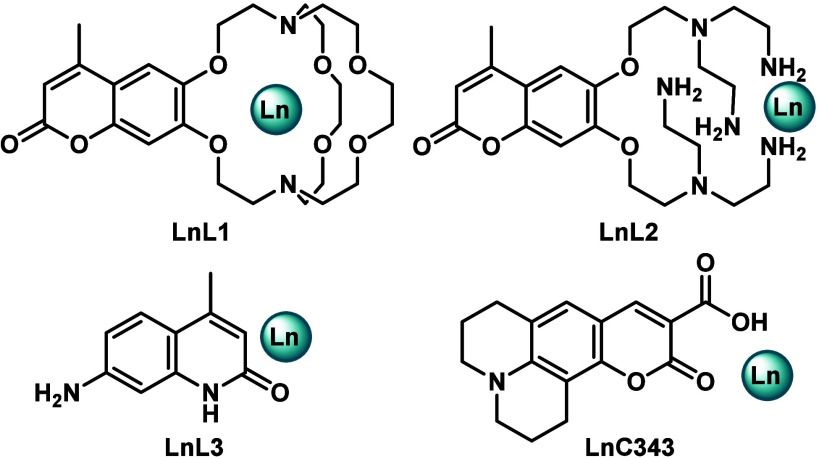
Lanthanide photocatalysts, with Ln = Eu or Sm.

Here, we report that a simple combination of commercially available coumarin 343 (**C343**; Figure 1) and a reducible Ln(III) salt is an excellent photocatalyst for several Ln(II)-mediated reductions. Reactions proceed under ambient conditions in aqueous solvent mixtures without Ir or Ru (co)catalysts. The absorption maximum of **C343** is at λ_abs_ = 446 nm, matching the output of the blue light-emitting diode (LED) common in photoreactors.^10^ Unlike **L1** and **L2**, **C343** does not have to be modified with a synthetically appended metal binding site. As a sacrificial reductant, 1 equiv of cheap ascorbic acid could be used. This catalyst afforded significantly improved yields of previously low-yielding reactions and catalyzed reactions that did not take place using **LnL1**–**LnL3**, greatly improving the operational simplicity of photocatalytic Ln(II)-mediated reductions.

Benzaldehyde (**1a**) was chosen as a model substrate. Irradiation with blue LED of a solution containing Sm(OTf)_3_ and **C343** (1:1, 0.1 equiv) under conditions optimized for **LnL2** [*N*,*N*-dimethylformamide (DMF)/H_2_O (4:1), [**1a**] = 30 mM, DIPEA (5 equiv), and LiCl (5 equiv)]^9^ afforded 15% conversion to compound **1c** in 24 h, which increased to 42% after 48 h. Longer reaction times did not improve upon this result (entry 1 in Table 1). Solvents and proton sources were then screened. Almost no product formed when increasing the water content to 50% (entry 2) or in tetrahydrofuran (THF)/H_2_O (4:1, entry 3). Conversion improved to 77% in MeCN/H_2_O (4:1, entry 4). The reaction gave a mixture of dl and *meso* isomers. Chiral additives [proline, 2,2′-dihydroxy-1,1′-binaphthyl, and 3-(heptafluoropropylhydroxymethylene)-(+)-camphorate] did not alter the product distribution (Table S2 of the Supporting Information). The optimized conditions (condition A) are thus 10% catalyst loading [Sm(OTf)_3_/**C343**, 1:1], 5 equiv each of DIPEA and LiCl, in MeCN/H_2_O (4:1, 35 mM), affording compound **1c** selectively in 77% yield; compound **1b** was not detected.

**Table 1:**
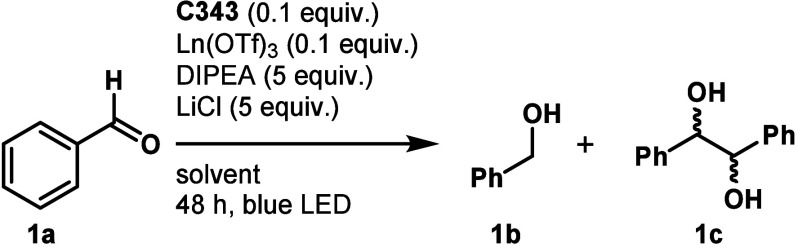
Optimization and Selected Control Experiments

entry	Ln^a^	solvent	conversion (%)^b^	dl/*meso* (%)^c^
1	Sm	DMF/H_2_O (4:1)	42	32/68
2	Sm	DMF/H_2_O (1:1)	<1	
3	Sm	THF/H_2_O (4:1)	<1	
4	Sm	MeCN/H_2_O (4:1)	77	49/51
5^d^	Sm	MeCN/H_2_O (4:1)	<1	
6^e^		MeCN/H_2_O (4:1)	4	43/57
7	Gd	MeCN/H_2_O (4:1)	7	31/69
8	Eu	MeCN/H_2_O (4:1)	91	38/62
9	Yb	MeCN/H_2_O (4:1)	98	36/64

a As Ln(OTf)_3_.

b Determined by gas chromatography–mass spectrometry (GC–MS).

c On the basis of the ratio of integrated signal areas of the separated product peaks.

d In the dark.

e With only **C343**.

The roles of the different components were probed. Changing the proton source to hexafluoroisopropanol or *tert*-BuOH decreased the yield, which is consistent with water-coordinating Sm(II) serving as a proton-coupled electron transfer agent.^11^ Diglyme and tetraglyme additives were detrimental to the yield; these bind Sm ions and block substrate access (Table S1 of the Supporting Information).^12^ The reaction did not work without light; therefore, Sm(II) was not generated by DIPEA directly (entry 5 in Table 1). Only 4–7% conversion was observed in the absence of Sm(OTf)_3_ (entry 6 in Table 1) or with Gd(OTf)_3_ instead of Sm(OTf)_3_ (entry 7 in Table 1). Both **EuC343** and **YbC343** afforded the pinacol product in excellent yields (entries 8 and 9). Differences between reactions carried out with reducible Ln(III) (Ln = Eu, Yb, or Sm) and non-reducible Gd(III) enable the identification of those processes that are dependent upon Ln redox activity. The results confirm that photochemical Ln(II) formation is essential for reactivity.

The substrate scope was explored under the optimized conditions but with a shortened reaction time, as most substrates were consumed within 24 h (Figure 1). **SmC343** was used as the catalyst because Sm(II) is a stronger reductant that Yb(II) and Eu(II);^13^ compounds **2a** and **7a** were unreactive with **YbC343**. Aldehydes bearing electron-neutral and electron-poor (**3a** and **4a**) substituents were selectively reduced in good to excellent yields to the corresponding pinacol products. Substrates carrying electron-donating *p*-methyl and *p*-F substituents were unreactive, likely due to their low electrophilicity. Free alcohols (**20a** and **21a**), a tertiary amine (**6a**), and a thioether (**5a**) were tolerated. The electron-donating ability of the tertiary amine may be reduced by the Lewis acidic additive. Reducible C=O functionalities, such as methyl ester (**10a**) and methyl ketone (**15a**), were retained. One aldehyde could be reduced selectively in 1,4-benzodialdehyde (**16a**). Multiple spots were present in the reaction mixture with 4-acetyl benzaldehyde, suggesting the reactivity of both the aldehyde and ketone groups. The pinacol product was isolated in a 40% yield. Low solubility of the starting material or the product was a problem in some cases, especially on a larger scale. For 4-formylbenzonitrile, changing the solvent to a DMF-based solvent resulted in a faster reaction (8 h versus 16 h) and good isolated yield (63%). Most products were obtained after purification with column chromatography, but compound **3c** precipitated out of the reaction mixture and was isolated by simple filtration.

Some reactions yielded the alcohol rather than the pinacol product. Both the nitro and aldehyde groups were reactive in 4-nitrobenzaldehyde; 4-nitrobenzylalcohol was the major product (36%). 4-(Methylsulfonyl)benzaldehyde was reduced to the alcohol in 22% yield in 24 h; the yield did not increase upon continuing the reaction for 48 h. The low yield of compounds **2b** and **2c** was due to the sluggish reaction of compound **2a**, as 70% of the starting naphthaldehyde remained after 48 h. Cinnamaldehyde formed the pinacol product in a 43% yield. Some of the low yields were due to purification problems, as a **C343**-derived byproduct was difficult to remove.

The low yields of some of the reactions and the purification problems prompted a search for a more inexpensive and water-soluble sacrificial reductant. Satisfyingly, 1 equiv of ascorbic acid could replace 5 equiv of DIPEA/LiCl (condition B in Figure 2). The absorption of **SmC343** in the presence of ascorbic acid remains at λ_abs_ = 446 nm, while DIPEA shifts it to λ_abs_ = 412 nm (Figure S4 of the Supporting Information). Similar or higher yields of the products were obtained in the same reaction time after a simpler workup procedure. Some products precipitated after aqueous–organic workup, eliminating the need for column chromatographic purification. The formation of **C343** degradation products was suppressed under condition B. **C343** could be recovered and reused with similar efficiency (**12c**, 40% using 2 mol %, section 2 of the Supporting Information). Compounds **3a**–**6a**, which were reduced efficiently under condition A, afforded the products in comparable yields under condition B. The pinacol products formed selectively even when a mixture of the alcohol and the pinacol product was formed under condition A (**2a**, **12a**, and **13a**). Several substrates that reacted only sluggishly under condition A gave the product under condition B in good to excellent yields (**10a**–**12a**, **18a**, and **21a**). 4-Methylbenzaldehyde (**7a**), 3,4,5-trimethoxybenzaldehyde (**8b**), 4-fluorobenzaldehyde (**14a**), and 4-methoxyacetophenone (**18a**) did not react under condition A but did under condition B. The catalyst loading could be decreased to 5 mol % and even 2 mol % (**5a**, **11a**, **12a**, and **15a**).

**Figure 2:**
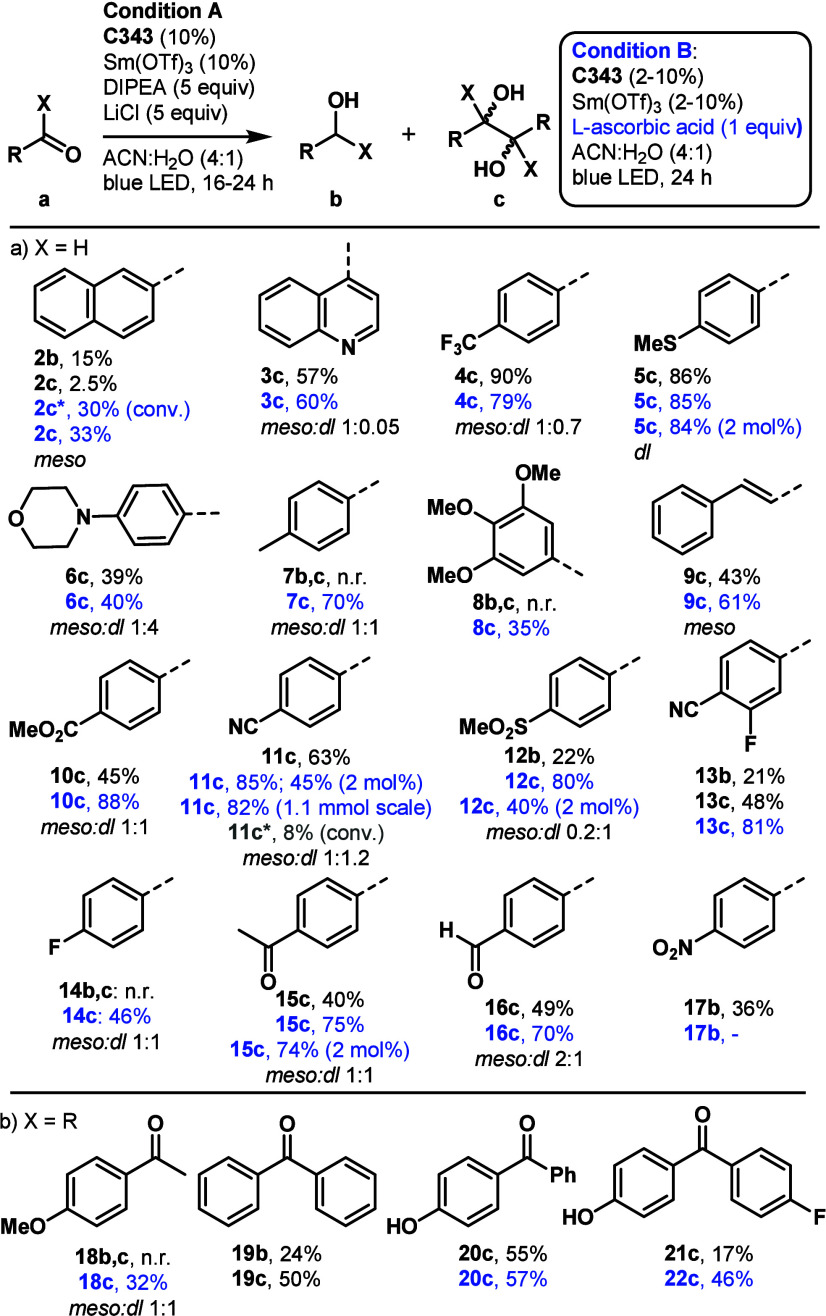
**SmC343**-catalyzed C=O reduction under condition A or B. 2c*, with both DIPEA:LiCl and ascorbic acid (2.5:2.5:1); 11c*, with Gd(OTf)_3_ (5 mol %), condition B.

SmI_2_ is a versatile reagent, and a useful catalytic alternative should promote a variety of transformations. Under condition B, C–halogen and N=N bonds could be reduced with 2–5% catalyst loading, while a P–Cl bond was reduced under condition A (Figure 3). The reductions could be followed by C–C and P–P bond formations. Benzyl bromide (**22a**) underwent dimerization (41%, **22b**) and intramolecular cyclization (10%, **22c**) under condition B. Decreasing the catalyst loading to 2 mol % improved the yield of compounds **22b** and **22c** to 56 and 11%, respectively. The reaction between 2,3-dimethoxybenzyl chloride and 6,7-dimethoxyisoquinoline yielded papaverine (**23b**) in 77% isolated yield, improving upon the previous photocatalyzed procedure (56%).^9^ Diazo compound **24a** was reduced to hydrazine (**24b**). *trans*-1,2-Diol (**25b**) was obtained in a highly stereoselective reaction. Catalytic halophosphine reduction afforded diphosphine (**26b**) and Ph_2_PH (**26c**) in 35 and 23% yields, respectively. Thus, **SmC343** catalyzes several typical reductions carried out by stoichiometric SmI_2_.

**Figure 3:**
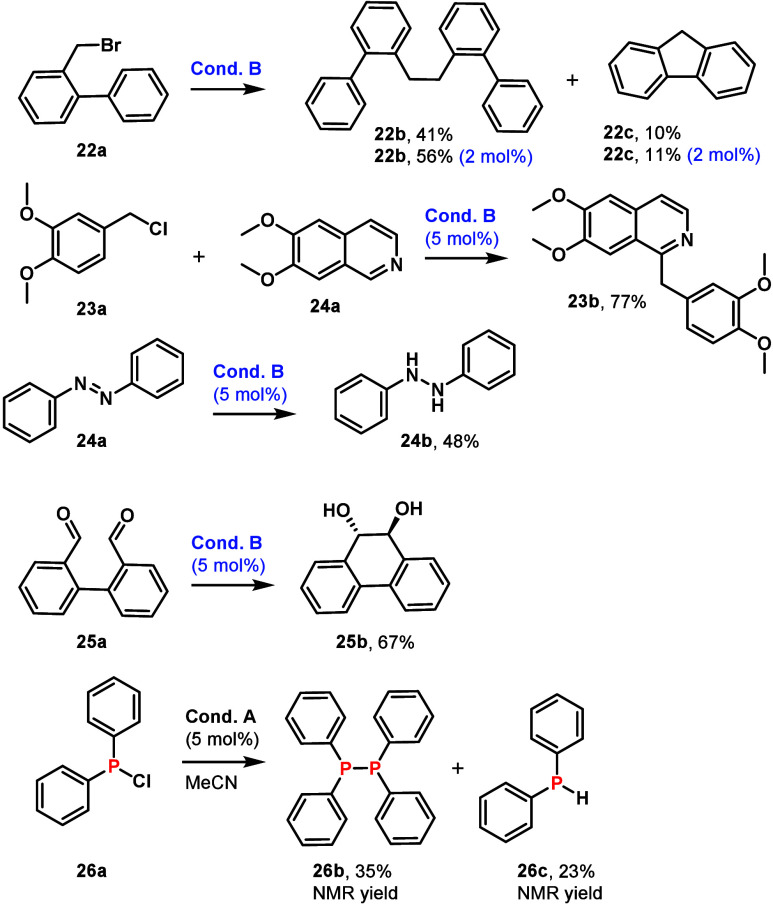
C–X, X=X, P–Cl, and C=O reductions using **SmC343**.

The proposed mechanism is shown in Scheme 1. Ln(III) is highly oxophilic and has high coordination numbers.^14^ The ultraviolet–visible (UV–vis) spectrum of **C343** changes upon the addition of 1 equiv of Sm(OTf)_3_ (Figures S2 and S3 of the Supporting Information). Eu(III) has information-dense luminescence^15^ and similar chemical properties to Sm(III); therefore, luminescence spectroscopic experiments were carried out with Eu(OTf)_3_. The luminescence lifetime of Eu(III) was 0.31 ms without **C343** and 0.24 ms with **C343** in MeCN (Table S4 of the Supporting Information). The shortening of the lifetime is consistent with **C343** binding bringing a quenching O–H oscillator^16^ into the inner sphere of Eu(III), and suggests that Ln(III) may coordinate to **C343**. Carbonyl reductions did not proceed without LiCl. Li^+^ likely acts as a Lewis acid (LA; Scheme 1), increasing the electrophilicity of the C=O group.

**Scheme 1:**
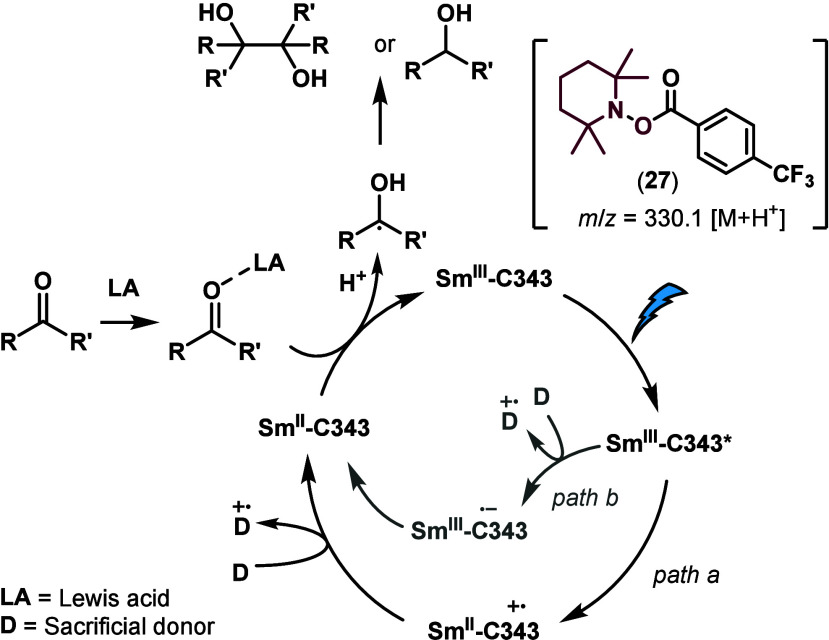
Proposed Mechanism of the Ln-Catalyzed C=O Reduction

Electron transfer from the S_1_ state of **C343** is exergonic.^17^**C343** fluorescence is quenched by Ln(III) (Ln = Gd, Sm, Eu, or Yb). Quenching is largest for Eu, Sm, and Yb, which can deplete excited-state **C343** (C343*) by electron and energy transfer in addition to the heavy atom effect, which is also possible for Gd(III). The **C343** fluorescence lifetime decreased in the presence of Eu(III), Yb(III), and Sm(III) (Figures S5–S12, S15, and S17–S24 and Table S3 of the Supporting Information). These observations are consistent with C343* being quenched by electron transfer to Ln(III) (path a in Scheme 1).

The reduction of Ln(III) by C343* yields Ln(II) and **C343**^**•**^ ^**+**^. This process is thermodynamically downhill for Ln = Eu, Sm, and Yb (Δ*G*_eT_ = −1.47, −0.63, and −0.72 eV, respectively; Table S5 of the Supporting Information). Irradiation of solutions containing equimolar amounts of **C343** and Ln(OTf)_3_ (Ln = Eu or Sm) in the presence of a radical trap, *N*-*tert*-butyl-α-phenylnitrone (PBN) gave a N-based radical from a N adduct visible in the electron paramagnetic resonance (EPR) spectrum recorded at room temperature, which is consistent with the formation of the **C343**^**•**^ ^**+**^ radical cation (Figure S28 of the Supporting Information). In the sample with Eu(OTf)_3_ and **C343** an additional wider EPR signal is present that we tentatively assign to a Eu(II) species (Figure S28 of the Supporting Information).

An alternative pathway (path b in Scheme 1) leading to Ln(II) involves the formation of **C343**^**•**^ ^**–**^ through the reduction of C343* by DIPEA [*E*_ox_ = 0.86 V versus saturated calomel electrode (SCE)^18^] or ascorbate (*E*_ox_ = 0.35 V versus SHE^19^). **C343**^**•**^ ^**–**^, in turn, could reduce Ln(III) and return to **C343**. All of these steps were calculated to be exergonic, with Δ*G*_eT_ values of −0.25 and −0.99 eV for the electron transfers from DIPEA and ascorbate to **C343**, respectively, and −0.33 eV for Sm(III) reduction by **C343**^**•**^ ^**–**^. Neither the **C343** fluorescence intensity nor lifetime changed in the presence of Gd(III) and increasing amounts of ascorbate (Figures S13, S14, and S16 and Table S3 of the Supporting Information). Thus, C343* is not quenched by ascorbate, and this pathway is not operational. These results support electron transfer taking place from C343* to Ln(III) under the reaction conditions to form a reactive Ln(II) species.

From photochemically generated Sm(II), inner-sphere electron transfer to the substrate is likely, as is the case for the analogous stoichiometric reactions.^20^ Irradiation of trifluoromethyl benzaldehyde in the presence of 3 equiv of the radical quencher (2,2,6,6-tetramethylpiperidin-1-yl)oxyl (TEMPO) formed compound **27** (*m*/*z* 330.1 [M + H]^+^), which is consistent with the intermediacy of the corresponding substrate-derived radical in the reaction.

In conclusion, a simple photocatalyst was identified for the promotion of a range of Sm(II)-mediated reductions and subsequent P–P and C–C bond formations. The catalyst consists of commercially available Sm(OTf)_3_ and **C343** that can be added directly to the reaction mixture. DIPEA and ascorbic acid were suitable terminal reductants, with the latter affording better yields for several substrates. This simple, cheap, and efficient catalyst renders photocatalytic Ln(II)-mediated reductions accessible and significantly more sustainable than the corresponding stoichiometric reactions.

## Data Availability

The data underlying this study are available in the published article and its Supporting Information.

## Supporting Information Available

Reaction optimization, chemical characterization of products, photophysical and electrochemical characterization of the catalyst, ^1^H, ^13^C, and ^19^F nuclear magnetic resonance (NMR) spectra for new compounds, and X-ray crystallography of compound **21c** (PDF)
