# Supplementary material for: Photocatalyst for Visible-Light-Driven Sm(II)-Mediated Reductions
Source: Org Lett. 2024 Dec 11;26(50):10752–6. doi: 10.1021/acs.orglett.4c03723 (PMC11667727; doi:10.1021/acs.orglett.4c03723)
Supplement: Supplementary file 1 — ol4c03723_si_001.pdf [file ol4c03723_si_001.pdf]

A photocatalyst for visible light-driven Sm(II)-mediated reductions

Monika Tomar, Caroline Bosch, Jules Everaert, Rohan Bhimpuria, Anders Thapper, Andreas Orthaber, K. Eszter Borbas*

[1]Department of Chemistry, Ångström Laboratory, Uppsala University; Uppsala, 75120, Sweden.

*eszter.borbas@kemi.uu.se

Table of Contents

1. **Materials and methods** 2
2. **Photocatalytic reaction procedures** 4
3. **Characterization data for compounds** 6
4. **Photophysical characterization** 9

   Quenching experiments 11

   C343 fluorescent lifetimes and Ln(III) luminescent lifetimes 16

5. **Electrochemistry** 21
6. **EPR Spectroscopy** 23
7. **GC-MS traces** 24
8. **X-ray crystallography** 25
9. **^{1}H, ^{13}C and ^{19}F NMR spectra** 27
10. **HR-MS data for new compounds** 45
11. **References** 48

1. Materials and methods

Materials. All chemicals were purchased from commercial sources. MeCN and DMF were obtained from an Inert Puresolv solvent purification system. All solid chemicals were dried under vacuum overnight before being used in a glovebox.

General Procedures. ^{1}H NMR (400 MHz), ^{13}C NMR (100 MHz) spectra were recorded on a JEOL 400 MHz instrument. Chemical shifts were referenced to residual solvent peaks and are given as follows: chemical shift (δ, ppm), multiplicity (s, singlet; br, broad; d, doublet, t, triplet; m, multiplet), coupling constant (Hz), integration. HR-ESI-MS analyses were performed at the Organisch Chemisches Institut WWU Münster, Germany or at the Stenhagen Analyslab AB, Mölndal. All compounds displayed the expected isotope distribution pattern.

Spectroscopy. All the measurements were performed in MeCN unless indicated otherwise. Quartz cells with 1 cm optical pathlengths were used for the room temperature measurements. The absorption spectra were recorded on a Varian Cary 100 Bio UV-Visible spectrophotometer. The emission and excitation spectra, lanthanide luminescence lifetimes, time-resolved spectra, and luminescence quantum yields were recorded on a Horiba FluoroMax-4P instrument. All emissions were corrected by the spectrometer's wavelength sensitivity (correction function). All measurements were performed at room temperature unless stated otherwise. Low-temperature measurements were done in quartz capillaries (0.2 cm optical pathlength) at 77 K by immersion in a liquid N_2-filled quartz Dewar.

Lifetimes were recorded 0.05 ms after pulsed excitation at the excitation maxima (λ_{ex}) of the ligand by measuring the decay of the lanthanide main emission peak (Eu(III): 616 nm) The increments after the initial delay were adjusted between 0.2–20 µs depending on the lifetime to have a good sampling of the decay. The obtained data were fitted by single and double exponential decay models in OriginPro 9, and the most reliable value was chosen according to the adjusted R^2 value and the shape of the residuals. A relative error of 10% is typically found among a series of measurements on the same sample.

The fluorescence lifetime decays in the nanosecond range were measured on a Spectrofluorometer FS5 system from Edinburgh Instruments. The system was equipped with picosecond pulsed light emitting diode EPLED-340 with excitation wavelength at 470 nm. The data were acquired in the 50 ns measurement range with peak preset at 10^4 counts in 1024 channels. The repetition rate of the excitation source was 10 MHz, and the synchronization delay was 80 s. The scatter light profile (prompt signal, black in the decay figures) was recorded for each experiment individually in the same quartz cuvette using diluted Ludox solution in HPLC water at 470 nm emission wavelength with similar parameters as were used for the measured sample (red in the decay figures). The obtained data were fitted in the Fluoracle software (green trace in the decay figures) using mono and biexponential reconvolution/tail fit model in Equations S1 and S2, where τ_1 is the sample lifetime, t is time represented in ns, B_1 is the population (100% in all cases) (Table S3).

$$R(t) = B_1 * \exp(-t/\tau_1) \qquad \text{(S1)}$$

$$R(t) = A + B_1 * \exp\left(-\frac{t}{\tau_1}\right) + B_2 * \exp\left(-\frac{t}{\tau_2}\right) \qquad \text{(S2)}$$

Gas chromatography- Mass spectrometry (GCMS). Photoreactions were monitored by GC-MS (Agilent 7890A GC and 5975 MSD system). Samples were injected using split injection (1 µL injection volume; split ratio: 100:1; 250 °C inlet temperature; flow rate: 120 mL/min). The temperature rate was set to 20 °C/min resulting in a 12.5 min total run time. He was used as a carrier gas at a flow rate of 1.2 mL/min. The column used was an Agilent 19091S-433: 325 °C: 30 m x 250 µm x 0.25 µm (front SS-inlet: He; out: vacuum). Mass spectrometer: Source temperature: 230 °C, Quad-temperature 150 °C.

Electrochemistry. Cyclic voltammograms (CV) were obtained at room temperature (~20 °C) using an AUTOLAB PGSTAT 100 potentiostat, or an AUTOLAB PGSTAT 204N potentiostat. The setup was equipped with a 3 mm glassy carbon (GC) working electrode, a Pt wire auxiliary electrode, and an Ag/AgCl as a reference electrode. Voltammograms are shown and values are reported for NHE (Table S5). Measurements were done in anhydrous DMF and MeCN with NBu_4PF_6 (0.1 M) as the supporting electrolyte. The voltammograms were recorded by scanning first toward more negative potential values (reduction). A step-potential of –0.9 mV was used for 100 mV/s scan rates.

A solution of NBu_4PF_6 (0.1 M) in MeCN (5 mL) taken from SPS (solvent purification system) was added to the electrochemical cell. The working electrode was polished with 0.05 µm alumina on a polishing pad, washed with water and ethanol and dried. This was repeated before each new sample. The three electrodes (GC working electrode, platinum wire auxiliary electrode, and Ag/AgCl reference electrode) were inserted into the cell setup followed by argon purging for 10 mins and a background scan was recorded with a scan rate of 100 mV/s, and two sweeps. The complexes were added to the solution (2 mM), purged again for 10 mins and the sample was recorded.

EPR Spectroscopy. EPR measurements at room temperature were performed using a Bruker EMX Micro spectrometer, equipped with an ER 4119HS resonator. EPR samples were prepared in a 1 mm capillary. EPR parameters: microwave frequency, 9.86 GHz; modulation frequency, 100 kHz.

Photoreaction setup. All reactions were performed in Microwave vials equipped with a stirring bar, in a dry glovebox [O_2 (<0.5 ppm), H_2O (<0.5 ppm)] with an Ar atmosphere. The vials were sealed with an electric black tape. 40 W blue LED lamp (Kessil A160WE Tuna Blue, λ_{max} = ~ 450 nm, set highest blue color and intensity) was used for irradiation (Figure S1). No filter was used. Reactions were stirred at 1000–1800 rpm at a distance of 3.5 cm from the light source. The full emission spectrum of the A160WE Tuna Blue light source given below [1].

- Producer: Kessil Lighting
- Catalog number: KES-A160WE TB
- EAN: 0092145339251
- Dimensions: 10.24 x 6.35cm (4" x 2.48")
- Coverage: Up to 24" surface diameter
- Power supply: 100-240 V AC (input), 19-24 V DC (output)
- Power consumption: 40 W

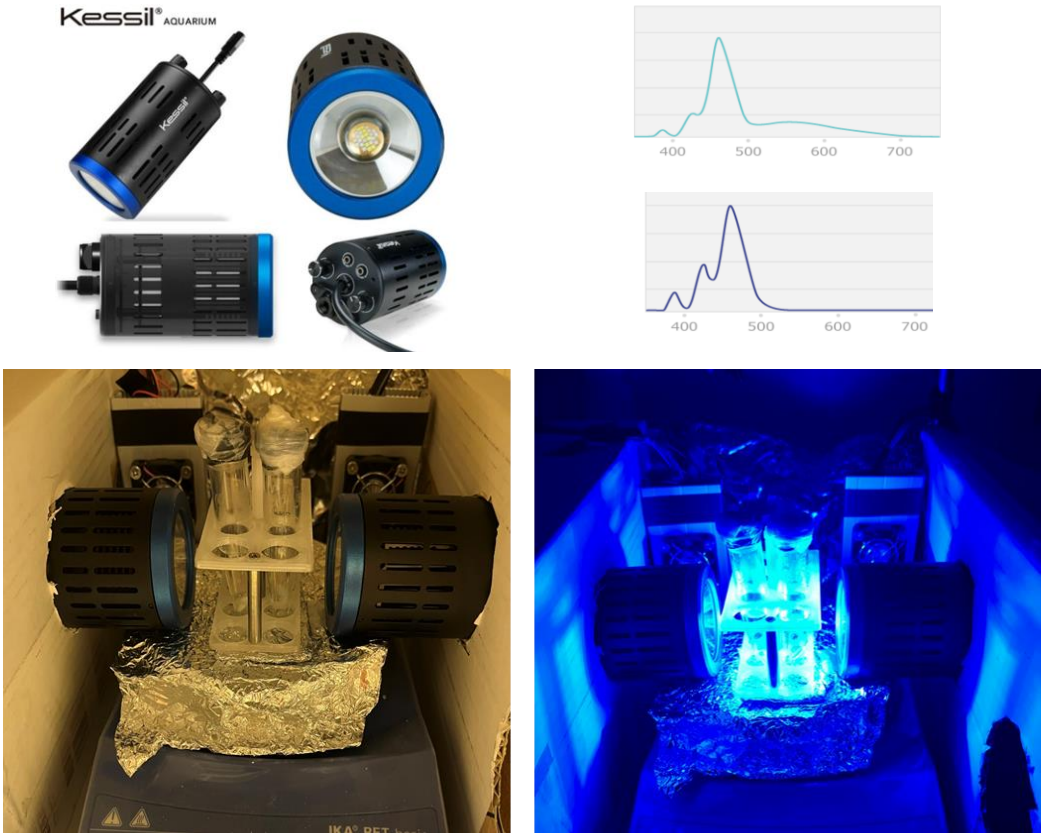

Figure S1. Top: lamp profile from the manufacturer website (https://www.kessil.com/index.php). Bottom: Kessil blue LED lamp setup for reactions in our system.

2. Photocatalytic reaction procedures

General procedures for small-scale reactions (0.039 mmol). In an Ar-filled glovebox, **C343** (0.1 equiv.), $Ln(OTf)_3$ (Ln = Eu, Gd, Sm, Yb) (0.1 equiv.), substrate (1.0 equiv.), sacrificial reductant or additive (10.0 equiv.) were added to a vial, followed by anhydrous MeCN (800 µL). The vial was sealed and covered with electrical tape, and taken out of the glovebox. Water (200 µL) was added. The reaction mixture was purged for 15 mins with Ar before placing it in the photoreactor and stirring for the specified time, while it changed color from yellow to dark orange. The reactions were analyzed using GC-MS.

General procedure for the scaled-up reactions with condition A (0.17–0.25 mmol). In an Ar-filled glovebox, **C343** (0.1 equiv.), $Sm(OTf)_3$ (0.1 equiv.), substrate (1.0 equiv.), DIPEA (5.0 equiv.) and LiCl (5.0 equiv.) were added to a vial, followed by anhydrous MeCN (to yield a solution 44 mM in substrate). The vial was sealed and covered with electrical tape, taken out of the glovebox, water (0.25x the volume of MeCN, to yield a solution 35 mM in substrate) was added, and the reaction mixture was purged with Ar for 15–20 mins before placing it in the photoreactor. The mixture was stirred for the specified time, during which it changed color from yellow to dark orange-brown. The reactions were followed by GC-MS or TLC analysis. Purification was performed with silica gel column chromatography using mixtures of ethyl acetate and heptane as eluents unless otherwise stated.

General procedure for the scaled-up reactions with condition B (0.17–0.25 mmol). In an Ar-filled glovebox, **C343** (0.1 equiv.), $Sm(OTf)_3$ (0.1 equiv.), substrate (1.0 equiv.), and L-ascorbic acid (1.0 equiv.) were added to a vial, followed by anhydrous MeCN (to yield a solution 44 mM in substrate). The vial was sealed and covered with electrical tape, taken out of the glovebox, water (0.25x the volume of MeCN, to yield a solution 35 mM in substrate) was added, and the reaction mixture was purged with Ar for 15–20 mins before placing it in the photoreactor. The mixture was stirred for the specified time, and followed by TLC analysis. After completion, water was added, and the mixture was extracted with ethyl acetate. The combined organic layers were dried over anhydrous sodium sulfate, filtered, and concentrated under reduced pressure to afford the crude product. Purification was performed by silica gel column chromatography using mixtures of ethyl acetate and heptane as eluents unless otherwise specified.

1.145 mmol-scale reaction using condition B. In an Ar-filled glovebox, **11a** (1.0 equiv., 1.145 mmol, 150.0 mg), **C343** (0.1 equiv., 0.057 mmol, 16.3 mg), $Sm(OTf)_3$ (0.1 equiv., 0.057 mmol, 34.2 mg), and L-ascorbic acid (1.0 equiv., 1.145 mmol, 200.0 mg) were added to a vial, followed by anhydrous MeCN (12.0 mL). The vial was sealed and covered with electrical tape, taken out of the glovebox, water (3.0 mL) was added to it, and the mixture was purged with Ar for 20 mins before placing it in the photoreactor. The mixture was stirred for 24 h. At this point TLC showed the full conversion of the starting material to a single product. Water was added to the mixture, and the mixture was extracted with ethyl acetate. The combined organic layers were dried over anhydrous Na_2SO_4, filtered, and the filtrate was concentrated under reduced pressure. Purification by silica gel column chromatography using mixtures of heptane and ethyl acetate as the eluent afforded **11c** in 82% yield (123 mg).

Reuse of the catalyst. After the reaction, part of the catalyst **C343**, was isolated by column chromatography using ethyl acetate in heptane (10%→90%) as the eluent. The evaporation of the solvent and drying of the residue under vacuum yielded an off-white solid. This isolated solid was reused in a reaction of **12a**, utilizing a catalyst loading of 2 mol%.

Table S1: Optimization using different solvents and proton sources

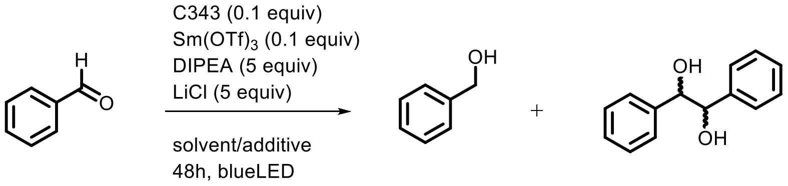

Entry	LnX_3	Solvent	Time	Conversion (%) [a]	dl:meso [b]
1	$Sm(OTf)_3$	DMF:H_2O (4:1)	24 h	15	40:60

2	Sm(OTf)$_3$	DMF:H_2O (4:1)	48 h	42	32:68
3	Sm(OTf)$_3$	DMF:H_2O (4:1)	48 h	<1	-
4	Sm(OTf)$_3$	MeCN:H_2O (4:1)	48 h	77	43:57
5	Sm(OTf)$_3$	THF:H_2O (4:1)	48 h	<1	-
4	Sm(OTf)$_3$	MeCN:diglyme (4:1)	48 h	25	45:55
5	Sm(OTf)$_3$	MeCN:HFIP(4:1)	48 h	76	45:55
6	Sm(OTf)$_3$	DMF:diglyme (4:1)	48 h	<1	-
8	Sm(OTf)$_3$	MeCN:^{t}BuOH (4:1)	48 h	46	36:64
9	Sm(OTf)$_3$	MeCN:tetraglyme (4:1)	48 h	34	35:65
10	Sm(OTf)$_3$	MeCN:^{t}BuOH (1:1)	48 h	34	48/52
11	Sm(OTf)$_3$	MeCN:tetraglyme (1:1)	48 h	<1	-

HFIP = Hexaflouroisopropanol

Table S2: Reactions with chiral additives. [a]

Entry	LnX$_3$	Additive	Conversion (%) [a]	dl/meso [b]
1	Sm(OTf)$_3$	-	77	43/57
2	Sm(OTf)$_3$	Proline (0.5 equiv.)	25	43/57
3	Sm(OTf)$_3$	2,2’-dihydroxy-1,1’-binaphthyl	6	-
4	Eu(III) tris[3-(heptafluoropropylhydroxymethylene)-(+)-camphorate		<1	-

[a] Reactions were carried out in ACN:H_2O (4:1) for 48 h under the optimized reaction conditions.

3. Characterization data for compounds

Products were purified by silica gel column chromatography using EtOAc containing 90%→0% heptane as the eluent. With the exception of **3c**, **12c**, **13c**, **20c,** and **21c** all products are known, and their characterization data matched the reported literature values. Products were isolated as mixtures of isomers.

Naphthalen-2-ylmethanol (2b).[2] Off-white solid (4 mg, 15%): ^{1}H NMR (400 MHz, DMSO-d_6) 7.89–7.82 (m, 4H), 7.50–7.45 (m, 3H), 5.35–5.32 (m, 1H), 4.67–4.66 (1H, m).

1,2-di(naphthalen-2-yl)ethane-1,2-diol (2c).[3] Yellowish solid (8 mg, 33%, meso isomer) yellowish solid was obtained as meso isomer. ^{1}H NMR (400 MHz, $CDCl_3$) 7.77–7.65 (m, 6H), 7.52–7.38 (m, 6H), 7.26–7.18 (m, 2H), 5.02 (s, 2H), 2.97 (s, 2H).

1,2-di(quinolin-4-yl)ethane-1,2-diol (3c). Yellowish solid (14 mg, 60%, meso:*d*/*l* = 1:0.05): ^{1}H NMR (400 MHz, DMSO-d_6) δ 8.81 (d, *J* = 4.5 Hz, 2H), 8.13 (dd, 2H), 8.01 (dd, *J* = 8.5, 1.0 Hz, 2H), 7.74–7.69 (m, 2H), 7.57 (d, *J* = 4.5 Hz, 2H), 7.52–7.48 (m, 2H), 5.87 (s, 2H), 5.63 (s, 2H); ^{13}C NMR (101 MHz, DMSO-d_6) 149.9, 148.5, 147.6, 129.5, 128.7, 126.7, 125.9, 124.3, 124.1, 120.3, 120.0, 72.5. HR-MS (ESI-Ion trap) *m*/*z*: $[M + H]^+$ Calcd for $C_{20}H_{16}O_2N_2H$ 317.1212; Found 317.1283.

1,2-bis(4-(trifluoromethyl)phenyl)ethane-1,2-diol (4c).[4] White solid (9 mg, 79%, meso:*d*/*l* = 1:0.67): ^{1}H NMR (400 MHz, DMSO-d_6) 7.62–7.53 (d, 4H), 7.42–7.36 (d, 4H), 5.65–5.61 (s, 2H), 4.9 (s, 2H, meso).

1,2-bis(4-(methylthio)phenyl)ethane-1,2-diol (5c).[5] Yellowish-white solid (16 mg, 85%, *d*/*l*): ^{1}H NMR (400 MHz, DMSO-d_6) δ 7.16 (d, *J* = 7.0 Hz, 4H), 7.1 (d, *J* = 5.0 Hz, 4H), 5.33 (dd, *J* = 3.0, 1.5 Hz, 1H), 5.20 (dd, *J* = 3.0, 1.5 Hz, 1H), 4.54–4.49 (m, 2H), 2.44 (s, 1H), 2.41 (s, 1H).

1,2-bis(4-morpholinophenyl)ethane-1,2-diol (6c).[6] Reddish solid (9 mg, 40%, *d*/*l*:meso (4:1)): ^{1}H NMR (400 MHz, DMSO-d_6) δ 7.11 (*d*/*l*, d, *J* = 8.0 Hz, 4H), 6.95 (meso, d, *J* = 8.0 Hz, 1H), 6.84 (*d*/*l*, d, *J* = 8.0 Hz, 4H), 6.75 (meso, d, *J* = 8.0 Hz, 1H), 4.42 (s, 3H), 3.73–3.71 (*d*/*l* + meso, m, 14H), 3.07–3.06 (*d*/*l* + meso, m, 12H); HR-MS (ESI-Ion trap) *m*/*z*: $[M + Na]^+$ Calcd for $C_{22}H_{26}N_2O_4Na$ 405.1790; Found 405.1784.

1,2-di-p-tolylethane-1,2-diol (7c).[7] Yellowish solid, 1:1 mixture of *d*/*l* and meso isomers (10 mg, 70%). ^{1}H NMR (400 MHz, DMSO-d_6) δ 7.13–7.06 (m, 4H), 7.06–7.02 (m, 4H), 6.98–6.94 (m, 8H), 5.25 (s, 2H), 5.09–5.03 (m, 2H), 4.53–4.46 (m, 4H), 2.30 (s, 6H), 2.26 (s, 6H).

1,2-bis(3,4,5-trimethoxyphenyl)ethane-1,2-diol (8c).[7] Yellowish solid (6 mg, 35%). ^{1}H NMR (400 MHz, DMSO-d_6) δ 6.51(2 H, s), 6.37 (2 H, s), 5.31 (s, 1H), 5.15 (s, 1H), 4.49 (s, 1H), 4.45 (s, 1H) 3.69 (s, 6H), 3.63 (s, 6H), 3.62 (s, 2H), 3.59 (s, 3H).

(1E,5E)-1,6-diphenylhexa-1,5-diene-3,4-diol (9c).[8] Off-white solid, meso isomer (11 mg, 61%). ^{1}H NMR (400 MHz, $CDCl_3$) δ 7.35 (m, 2H), 6.72 (dd, *J* = 16.0, 9.0 Hz, 1H), 6.29 (dt, *J* = 11.5, 4.5 Hz, 1H), 4.44 (d, *J* = 6.0 Hz, 1H), 4.29 (d, *J* = 6.0 Hz, 1H), 1.54 (d, *J* = 6.0 Hz, 1H), 1.43 (d, *J* = 6.0 Hz, 1H).

Dimethyl 4,4'-(1,2-dihydroxyethane-1,2-diyl)dibenzoate (10c).[5] White solid, 1:1 mixture of *d*/*l* and meso isomers (19 mg, 88%). ^{1}H NMR (400 MHz, DMSO-d_6) δ 7.84 (d, *J* = 8.0 Hz, 4H), 7.24 (d, *J* = 8.0 Hz, 4H), 4.78 (s, 2H), 3.87 (s, 6H).

4,4'-(1,2-dihydroxyethane-1,2-diyl)dibenzonitrile (11c).[9] Reddish solid, 1:1.2 mixture of *d*/*l* and meso isomers (15 mg, 85%): ^{1}H NMR (400 MHz, DMSO-d_6) δ ^{1}H NMR (400 MHz, DMSO-d_6) δ 7.70 (d, *J* = 8.0 Hz, 4H), 7.46 (d, *J* = 8.0 Hz, 4H), 5.62 (dd, *J* = 3.0, 1.5 Hz, 2H), 4.8 (dd, *J* = 3.0, 1.5 Hz, 2H).

Methyl 4-(hydroxymethyl)benzenesulfinate (12b).[10] Off-white solid (4 mg, 22%) (400 MHz, DMSO-d_6) δ 7.88 (d, *J* = 8.2 Hz, 2H), 7.57 (d, *J* = 8.5 Hz, 2H), 5.46 (t, *J* = 5.7 Hz, 1H), 4.61 (d, *J* = 5.1 Hz, 2H), 3.20 (s, 3H).

Dimethyl 4,4'-(1,2-dihydroxyethane-1,2-diyl) dibenzenesulfinate (12c). Yellowish solid 1:0.2 mixture of *d*/*l* and meso isomers (16 mg, 80%): ^{1}H NMR (400 MHz, DMSO-d_6) δ 7.85 (*d*/*l*, d, *J* = 8.0

Hz, 4H), 7.79 (meso, d, *J* = 8.0 Hz, 1H), 7.56 (*d*/*l*, d, *J* = 8.5 Hz, 4H), 7.48 (meso, d, *J* = 8.5 Hz, 1H), 5.65 (s, 2H), 4.66 (s, 2H), 3.19 (*d*/*l*, s, 6H), 3.17 (meso, s, 1H); ^{13}C NMR (101 MHz, DMSO-d_6) δ 149.2, 139.4, 128.2, 126.2, 76.2, 43.6; HR-MS (ESI-Ion trap) *m*/*z*: [M + Na]$^+$ Calcd for $C_{16}H_{18}O_6S_2Na$ 393.0442; Found 393.0443.

2-fluoro-4-(hydroxymethyl)benzonitrile (13b).[11] Off-white solid (5 mg, 21%): ^{1}H NMR (400 MHz, DMSO-d_6) δ 7.85–7.80 (m, 2H), 7.41–7.34 (m, 2H), 7.28–7.24 (m, 2H), 5.84 (s, 2H), 4.87 (s, 1H), 4.69 (s, 1H).

4,4'-(1,2-dihydroxyethane-1,2-diyl)bis(2-fluorobenzonitrile) (13c). Yellow-green solid (16 mg, 81%): ^{1}H NMR (400 MHz, DMSO-d_6) δ 7.87–7.77 (ddd, *J* = 8.0, 7.0, 3.0 Hz, 2H), 7.42–7.33 (m, 2H), 7.31–7.21 (ddd, *J* = 13.5, 8.0, 1.5 Hz, 2H), 5.92–5.80 (ddd, *J* = 12.5, 3.5, 1.5 Hz, 2H), 4.92–4.85 (d, *J* = 5.0 Hz, 1H), 4.73–4.65 (d, *J* = 3.5 Hz, 1H); ^{13}C NMR (101 MHz, DMSO-d_6) 164.7, 162.2, 152.8, 134.4, 125.7, 116.3, 99.9, 76.8, 50.0; HR-MS (ESI-Ion trap) *m*/*z*: [M + H]$^+$ Calcd for $C_{16}H_{10}N_2F_2O_2H$ 301.0710; Found 301.0783; [M + Na]$^+$ Calcd for $C_{16}H_{10}N_2F_2O_2Na$ 323.0608; Found 323.0602.

1,2-bis(4-fluorophenyl)ethane-1,2-diol (14c).[12] Off-white solid, 1:1 mixture of *d*/*l* and meso isomers (6 mg, 46%): ^{1}H NMR (400 MHz, $CDCl_3$) δ 7.20–7.09 (m, 4H), 7.07–7.02 (m, 4H), 7.00–6.95 (m, 4H), 6.94–6.88 (m, 4H), 4.83 (*d*/*l*, s, 2H), 4.63 (meso, s, 2H), 2.77 (s, 4H).

1,1'-((1,2-dihydroxyethane-1,2-diyl)bis(4,1-phenylene))bis-(ethan-1-one) (15c).[13] Off-white solid, 1:1 mixture of *d*/*l* and meso isomers (18 mg, 75%).

meso: ^{1}H NMR (400 MHz, DMSO-d_6) δ 7.80–7.77 (m, 4H), 7.28 (d, *J* = 8.0 Hz, 4H), 5.67 (s, 2H), 4.76 (s, 2H), 2.53 (d, *J* = 1.0 Hz, 6H).

d/*l* isomer: ^{1}H NMR (400 MHz, DMSO-d_6) δ 7.85 (d, *J* = 8.0 Hz, 2H), 7.37 (d, *J* = 8.0 Hz, 2H), 4.68 (s, 1H), 2.55 (d, *J* = 1.0 Hz, 3H).

4,4'-(1,2-dihydroxyethane-1,2-diyl)dibenzaldehyde (16c).[14] Yellowish solid, 1:2 mixture of *d*/*l* and meso isomers (13 mg, 70%).

d/*l* isomer: ^{1}H NMR (400 MHz, DMSO-d_6) δ 9.97 (s, 2H), 7.80 (d, *J* = 8.0 Hz, 4H), 7.46 (d, *J* = 8.0 Hz, 4H), 5.70 (s, 1H), 4.81 (s, 1H).

meso: ^{1}H NMR (400 MHz, DMSO-d_6) δ 9.93 (s, 1H), 7.73 (d, *J* = 8.0 Hz, 2H), 7.36 (d, *J* = 8.0 Hz, 2H), 5.62 (s, 2H), 4.72 (s, 2H).

(4-nitrophenyl)methanol (17b).[15] white solid (8 mg, 36%): ^{1}H NMR (400 MHz, DMSO-d_6) δ 8.20 (d, *J* = 8.0 Hz, 2H), 7.67–7.51 (m, 2H), 4.64 (s, 2H).

2,3-bis(4-methoxyphenyl)butane-2,3-diol (18c).[16] Off-white solid, 1:1 mixture of *d*/*l* and meso isomers (5 mg, 32%): ^{1}H NMR (400 MHz, DMSO-d_6) δ 7.30 (d, *J* = 8.0 Hz, 4H), 6.96 (d, *J* = 8.0 Hz, 4H), 6.76 (d, *J* = 8.0 Hz, 4H), 6.65 (d, *J* = 8.0 Hz, 4H), 4.82 (s, 2H), 4.71 (s, 2H), 3.72 (s,6), 3.68 (s, 6H), 1.40 (s, 6H), 1.24 (s, 6H).

Diphenylmethanol (19b).[7] Off-white solid (4.5 mg, 24%): ^{1}H NMR (400 MHz, $CDCl_3$) δ 7.40–7.18 (m, 8H), 7.15–7.19 (m, 2H), 5.86 (s, 1H), 2.22 (s, 1H).

1,1,2,2-tetraphenylethane-1,2-diol (19c).[17] Off-white solid (10 mg, 50%): ^{1}H NMR (400 MHz, DMSO-d_6) δ 7.77–7.64 (m, 12H), 7.61–7.52 (m, 8H), 5.98–5.91 (s, 1H), 5.70–5.65 (s, 1H).

1,2-bis(4-hydroxyphenyl)-1,2-diphenylethane-1,2-diol (20c). Off-white solid, mixture of *d*/*l* and meso isomers (10 mg, 57%): ^{1}H NMR (400 MHz, DMSO-d_6) δ 9.05(s, 3H), 7.39–7.23 (m, 6H), 7.16–6.98 (m, 15H), 6.49–6.38 (m, 6H), 5.54 (s, 3H); ^{13}C NMR (101 MHz, DMSO-d_6) δ 155.2, 155.1, 147.1, 136.9, 130.4, 130.2, 129.2, 129.1, 126.0, 125.9, 125.52, 125.48, 112.9, 112.8, 82.5; HR-MS (ESI-Ion trap) *m*/*z*: [M + Na]$^+$ Calcd for $C_{26}H_{22}O_4Na$ 421.1416; Found 421.1411.

1,2-bis(4-fluorophenyl)-1,2-bis(4-hydroxyphenyl)ethane-1,2-diol (21c). Off-white solid (9 mg, 46%): ^{1}H NMR (400 MHz, DMSO-d_6) δ 7.36–7.25 (dd, *J* = 8.8, 5.8 Hz, 4H), 7.10–7.01 (m, 4H), 6.92–6.81 (m, 4H), 6.50–6.43 (m, 4H), 5.73 (s, 2H); ^{13}C NMR (101 MHz, DMSO-d_6) δ 162.9, 155.9, 131.5,

131.4, 130.8, 113.5, 113.2, 113.0, 82.7; ^{19}F NMR (376 MHz, DMSO-d_6) δ 117.1; HR-MS (ESI-Ion trap) m/z: $[M + Na]^+$ Calcd for $C_{26}H_{20}O_4F_2Na$ 457.1227; Found 457.1221.

1,2-di([1,1'-biphenyl]-2-yl)ethane (22b).[18] White solid (9 mg, 41%): ^{1}H NMR (400 MHz, $CDCl_3$) δ 7.59–7.53 (1H, m), 7.50–7.34 (7 H, m), 7.31–7.28 (1H, m), 4.65–4.61 (2H, s).

9H-Fluorene (22c).[19] Colorless solid (2 mg, 10%): ^{1}H NMR (400 MHz, $CDCl_3$) δ 7.80 (d, J = 7.5 Hz, 2H), 7.56 (d, J = 7.5 Hz, 2H), 7.39 (m, 2H), 7.31 (td, J = 7.5, 1.0 Hz, 2H), 3.91 (s, 2H).

Papaverine (23b).[20] Yellow solid (4 mg, 77%): ^{1}H NMR (400 MHz, $CDCl_3$) δ 8.32 (d, J = 5.5 Hz, 1H), 7.81 (d, J = 5.7 Hz, 1H), 7.58 (s, 1H), 7.22 (s, 1H), 6.90 (d, J = 8.0 Hz, 1H), 6.76 (d, J = 8.0 Hz, 1H), 4.96 (s, 2H), 4.10 (d, J = 1.5 Hz, 3H), 4.01 (d, J = 1.5 Hz, 3H), 3.87 (d, J = 1.5 Hz, 3H), 3.81 (d, J = 1.5 Hz, 3H).

1,2-diphenylamine (24b).[16] White solid (8 mg, 48%): ^{1}H NMR (400 MHz, $CDCl_3$) δ 7.23–7.18 (m, 4H), 6.87–6.80 (m, 6H), 5.63 (brs, 2H).

9,10-dihydrophenanthrene-9,10-diol (25b).[16] Off-white solid (5 mg, 67%): ^{1}H NMR (400 MHz, $CDCl_3$) δ 7.75–7.74 (m, 2H), 7.68–7.66 (m, 2H), 7.43–7.36 (m, 4H), 4.76 (s, 2H), 2.09 (s, 2H).

1,1,2,2-tetraphenyl-diphosphine (26b).[21] The yield was calculated from the ^{31}P NMR spectrum according to a literature procedure using tris(4-fluorophenyl) phosphine as an internal standard.[21] ^{31}P NMR (CD_3CN): δ = –16.3 ppm.

Diphenylphosphine (26c).[21] The yield was calculated from the ^{31}P NMR spectrum according to a literature procedure using tris(4-fluorophenyl) phosphine as an internal standard.[21] ^{31}P NMR (CD_3CN): δ = –38.8 ppm (d, $^1J_{P\text{-}H}$ = 222 Hz).

4. Photophysical characterization

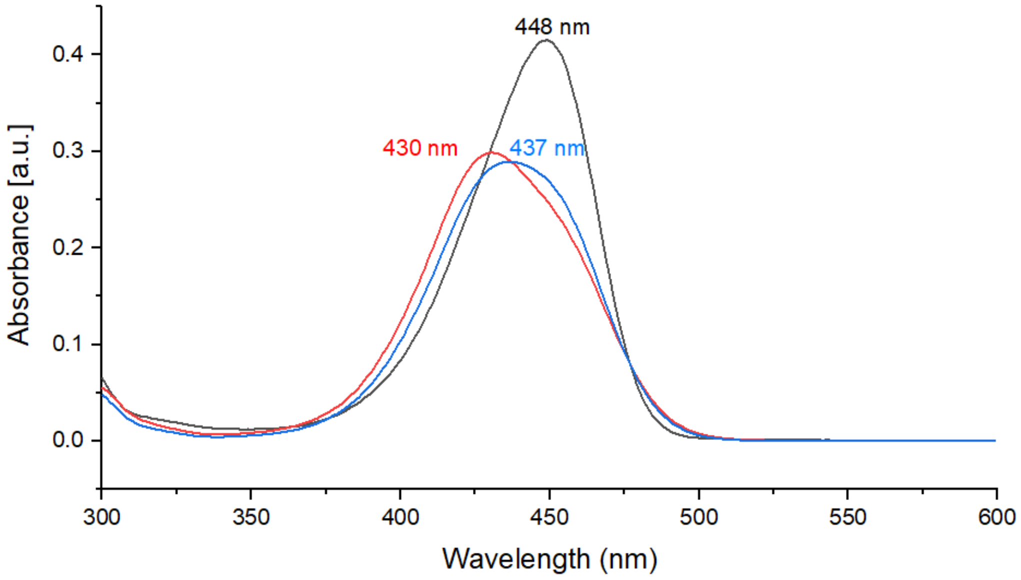

Figure S2. UV-Vis absorption spectra of **C343** (black), **C343** + Sm(OTf)$_3$ (1:1, red), and **C343** + Gd(OTf)$_3$ (blue) in MeCN. All components had a concentration of 17 µM.

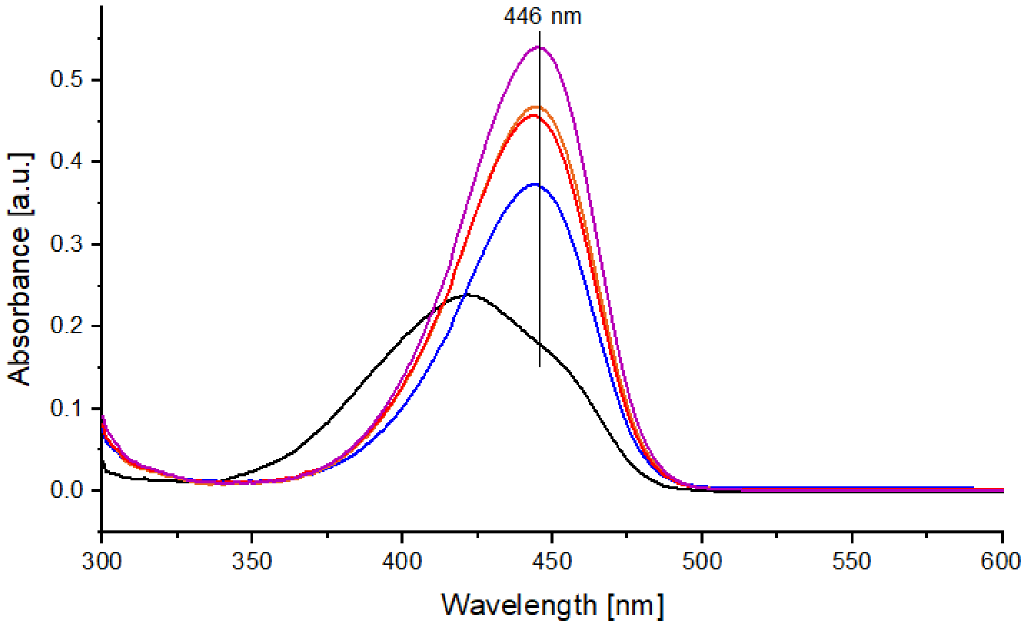

Figure S3. UV-Vis absorption spectra of **C343** (black), **C343** + Sm(OTf)$_3$ (1:1, red), **C343** + Gd(OTf)$_3$ (blue), **C343** + Eu(OTf)$_3$ (orange) and **C343** + Yb(OTf)$_3$ (purple) in MeCN:H_2O (4:1). All components had a concentration of 17 µM.

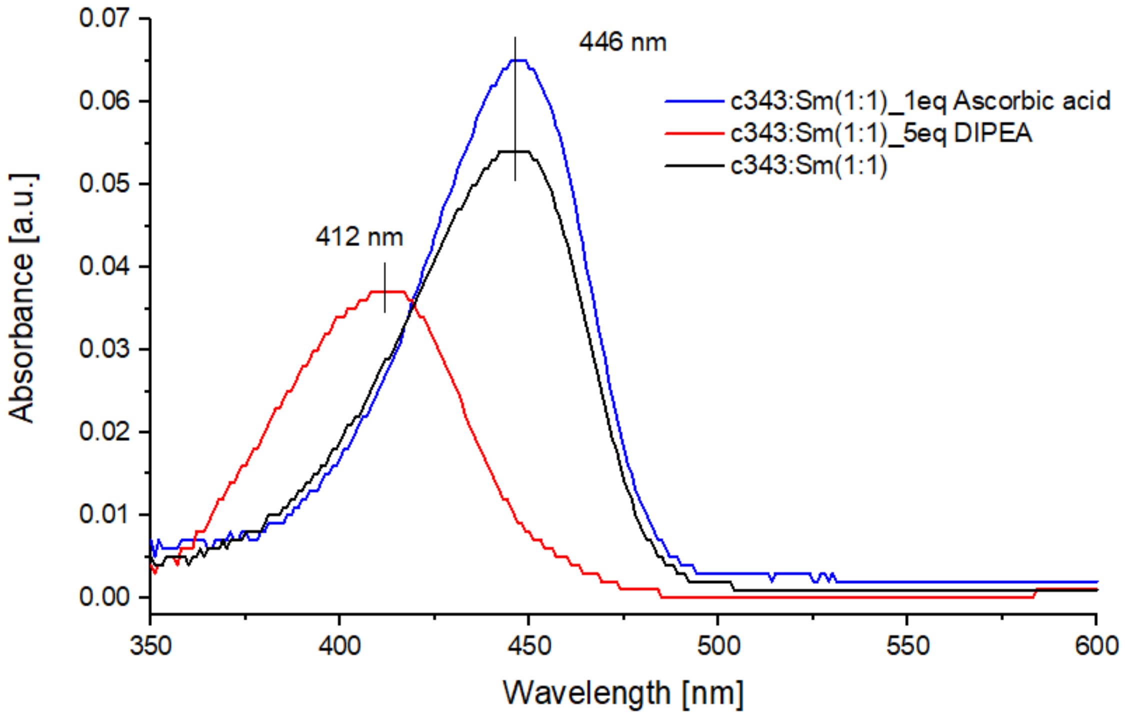

Figure S4. UV-Vis absorption spectrum of **C343** under different conditions in MeCN:H_2O (4:1); [**C343**] = 2 µM.

Quenching experiments

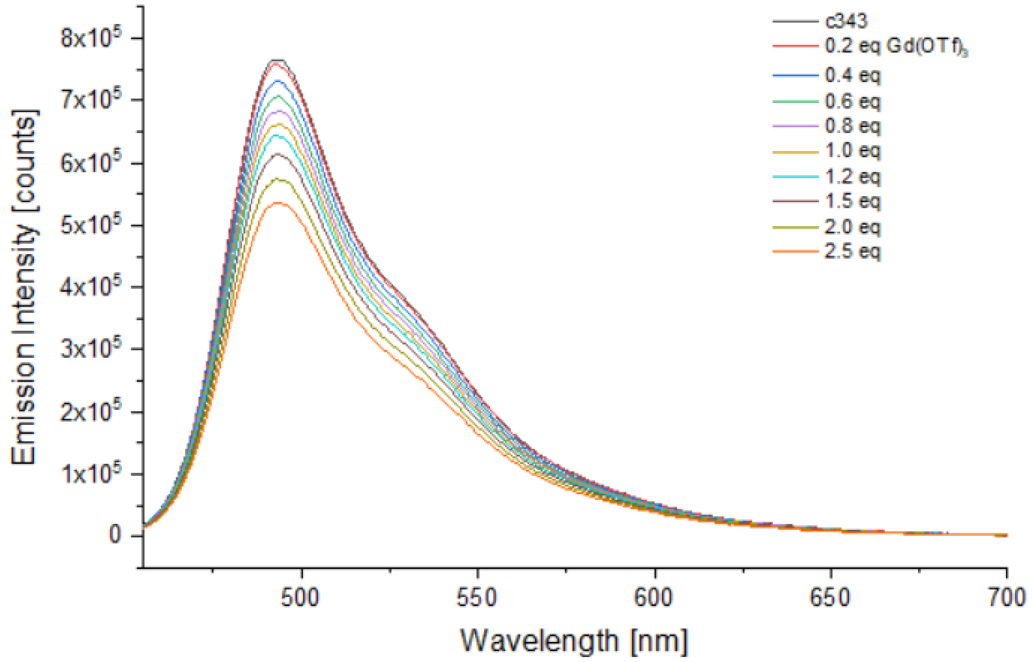

Figure S5. Changes in the steady-state fluorescence spectrum of **C343** upon addition of $Gd(OTf)_3$ in $MeCN:H_2O$ (4:1); λ_{ex} = 445 nm.

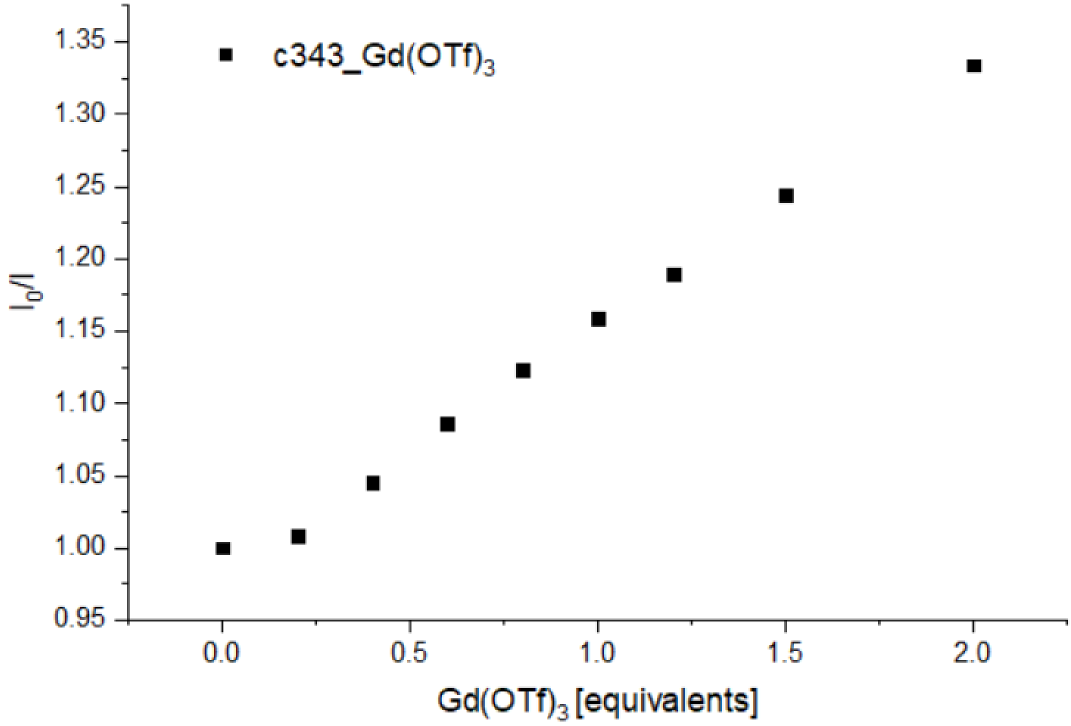

Figure S6. Quenching of the fluorescence emission of **C343** with $Gd(OTf)_3$ in $MeCN:H_2O$ (4:1); λ_{ex} = 445 nm.

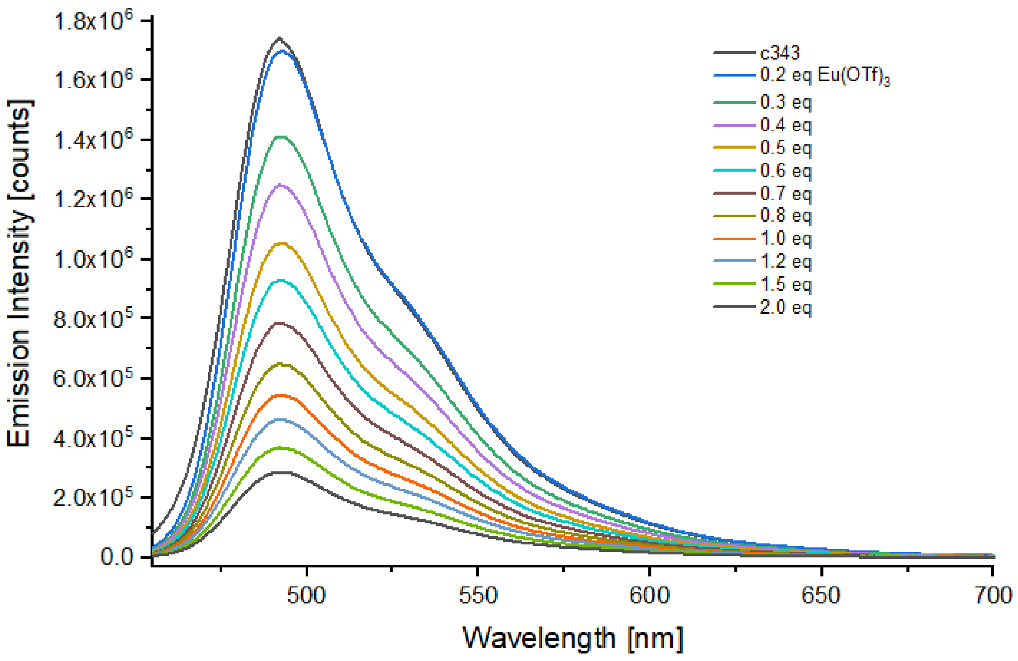

Figure S7. Changes in the steady-state fluorescence spectrum of **C343** upon addition of $Eu(OTf)_3$ in MeCN:H_2O (4:1); λ_{ex} = 445 nm.

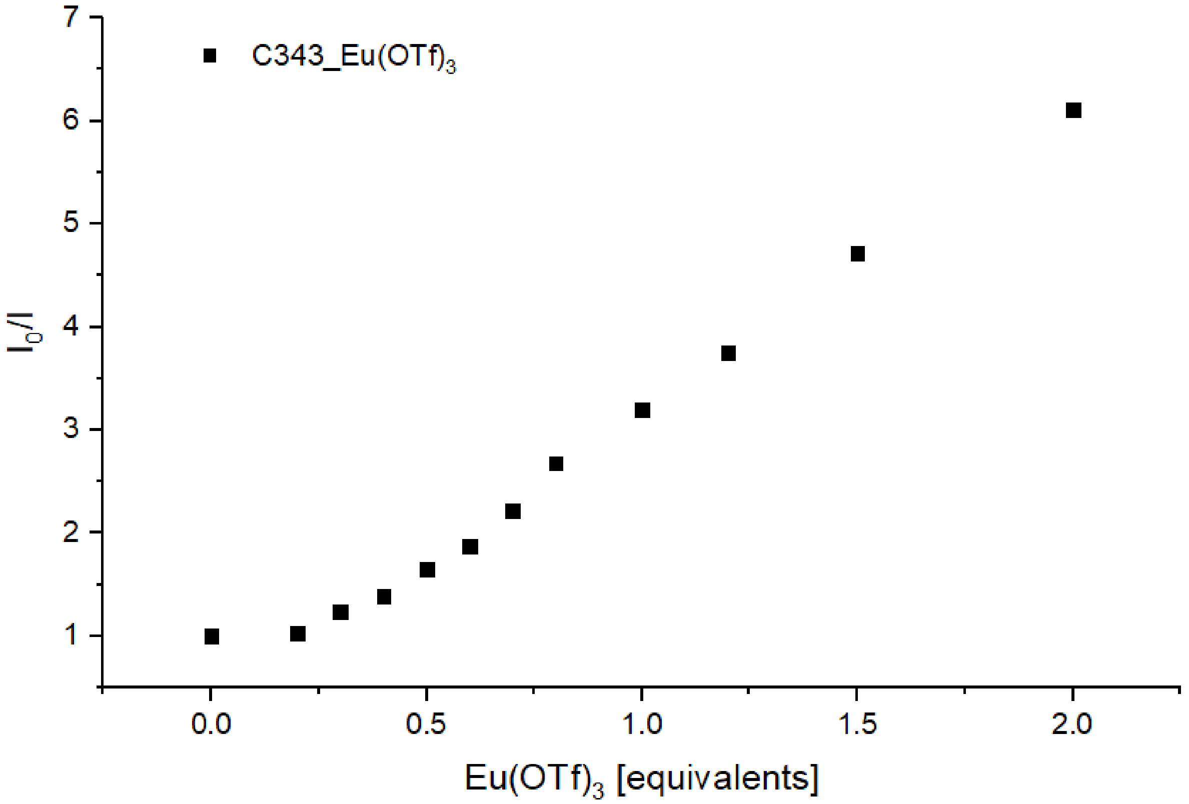

Figure S8. Quenching of the fluorescence emission of **C343** with $Eu(OTf)_3$ in MeCN:H_2O (4:1); λ_{ex} = 445 nm.

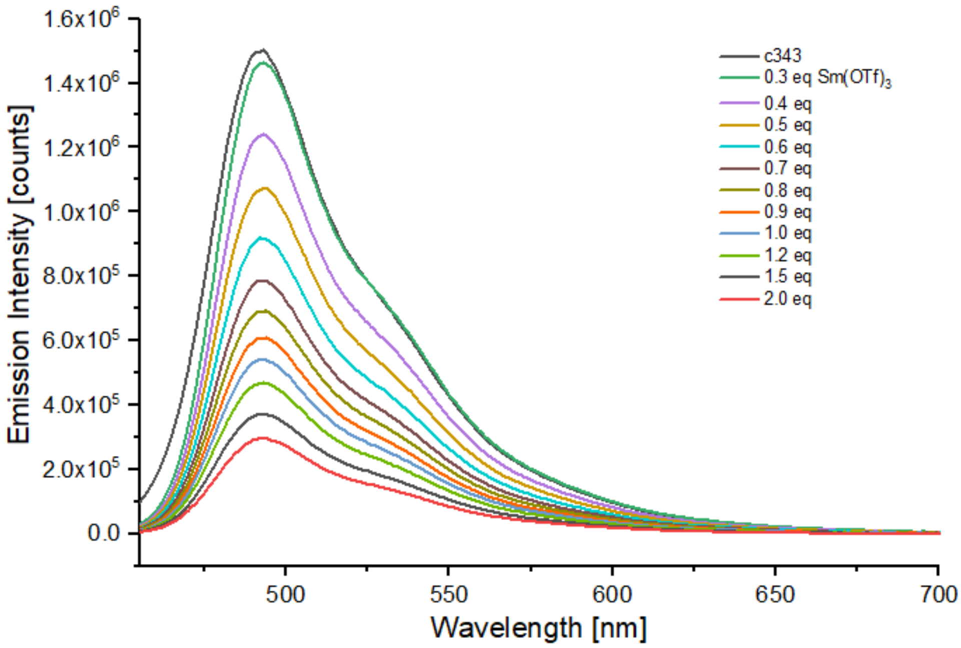

Figure S9. Changes in the steady-state fluorescence spectra of **C343** upon the addition of $Sm(OTf)_3$ in MeCN:H_2O (4:1); λ_{ex} = 445 nm.

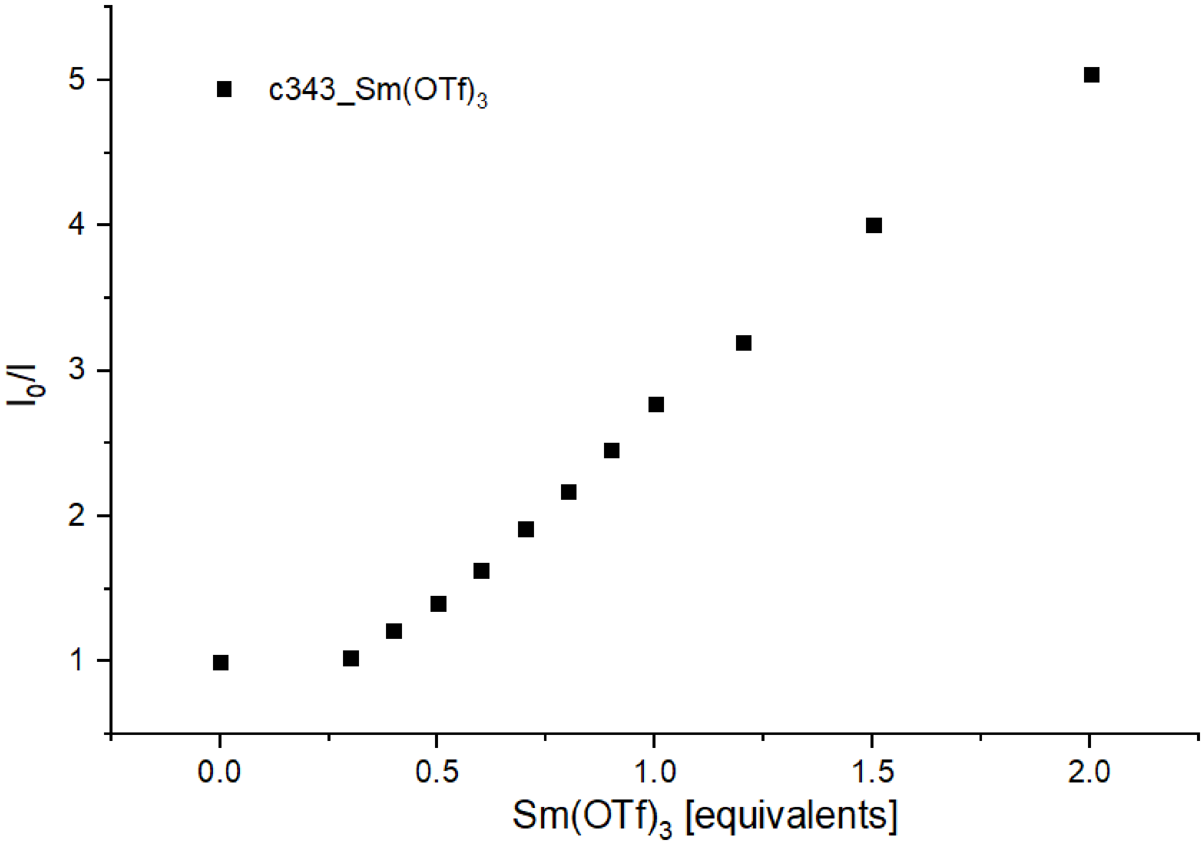

Figure S10. Quenching of the fluorescence emission of **C343** upon addition of $Sm(OTf)_3$ in MeCN:H_2O (4:1); λ_{ex} = 440 nm.

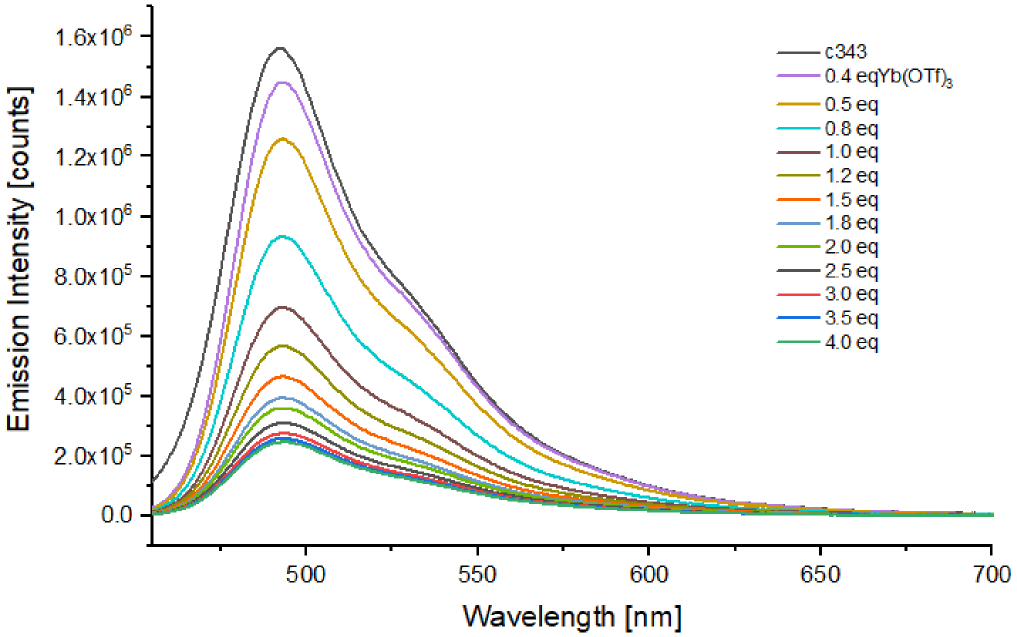

Figure S11. Changes in the steady-state fluorescence spectrum of **C343** upon addition of $Yb(OTf)_3$ in MeCN:H_2O (4:1); λ_{ex} = 445 nm.

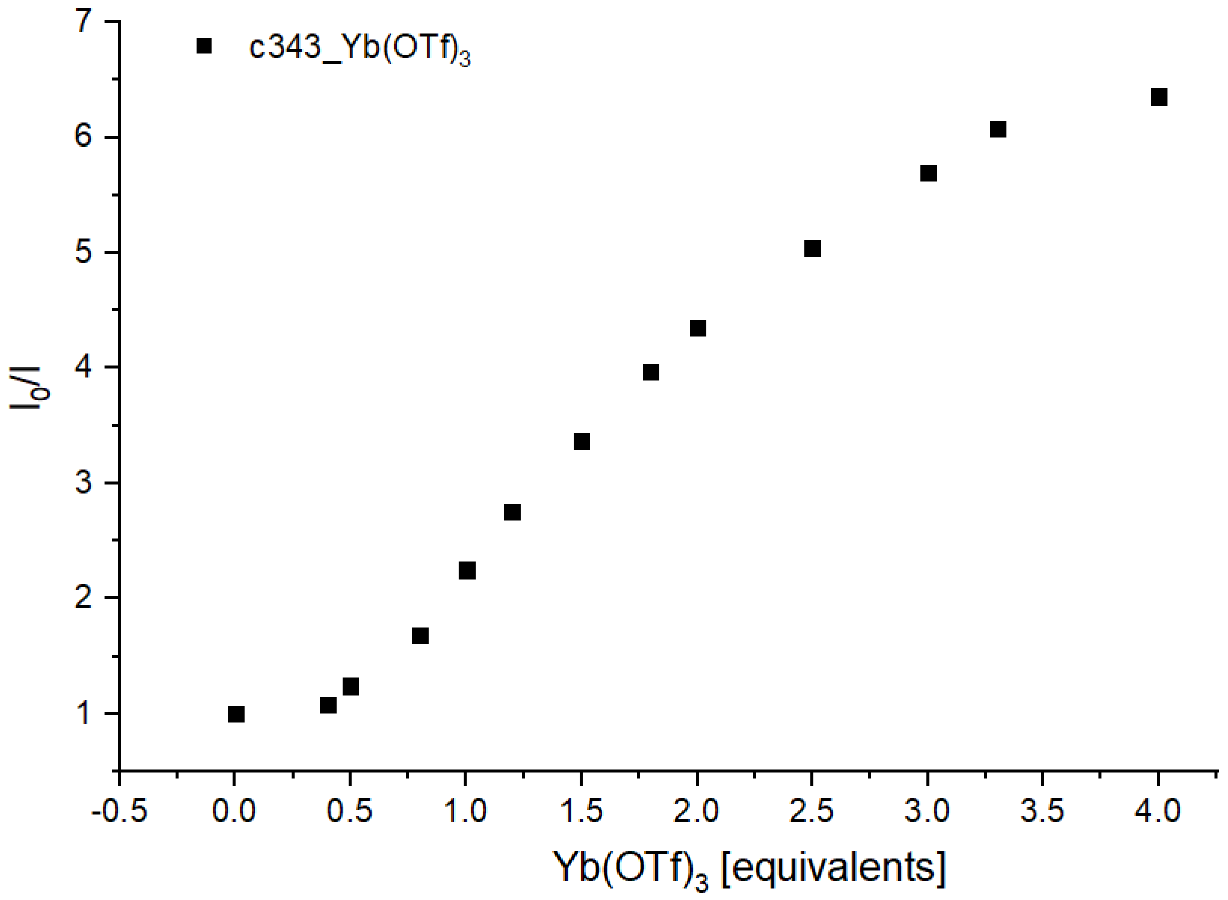

Figure S12. Quenching of the fluorescence emission **C343** upon addition of $Yb(OTf)_3$ in MeCN:H_2O (4:1); λ_{ex} = 445 nm.

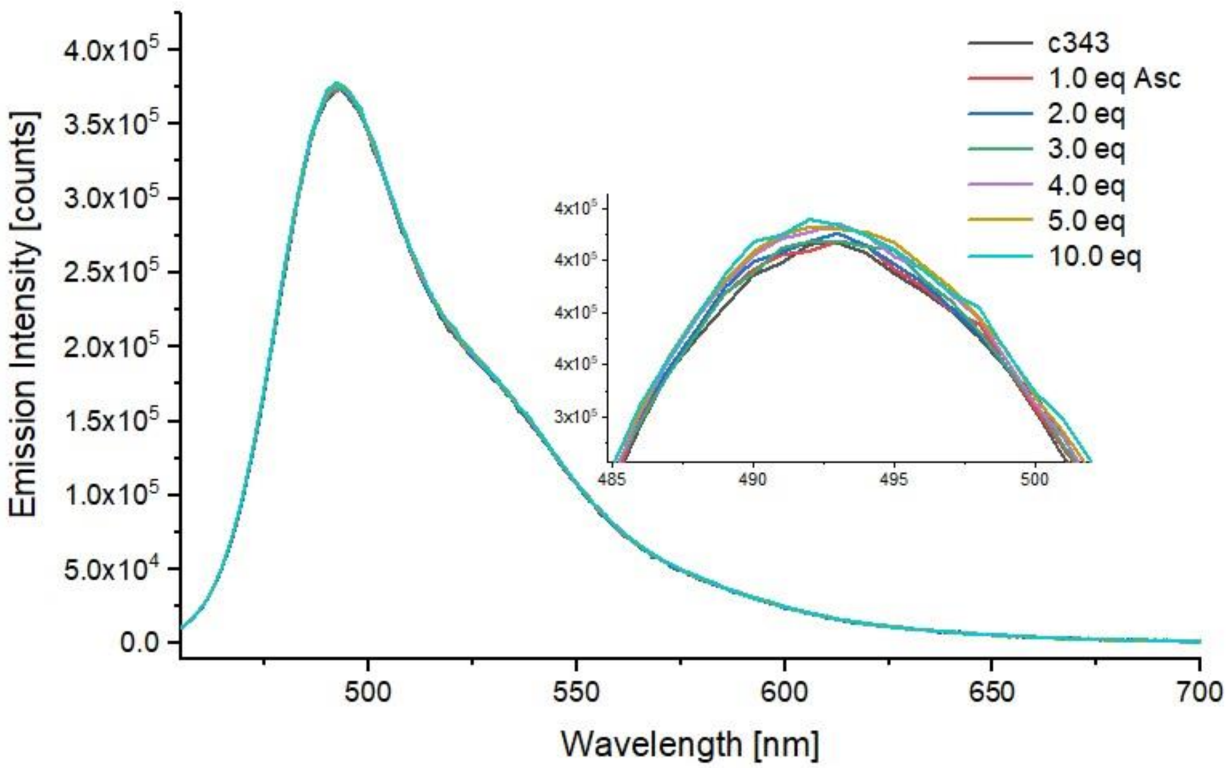

Figure S13. Fluorescence emission spectra of **C343** in the presence of increasing amounts of ascorbic acid in MeCN:H_2O (4:1); λ_{ex} = 445 nm.

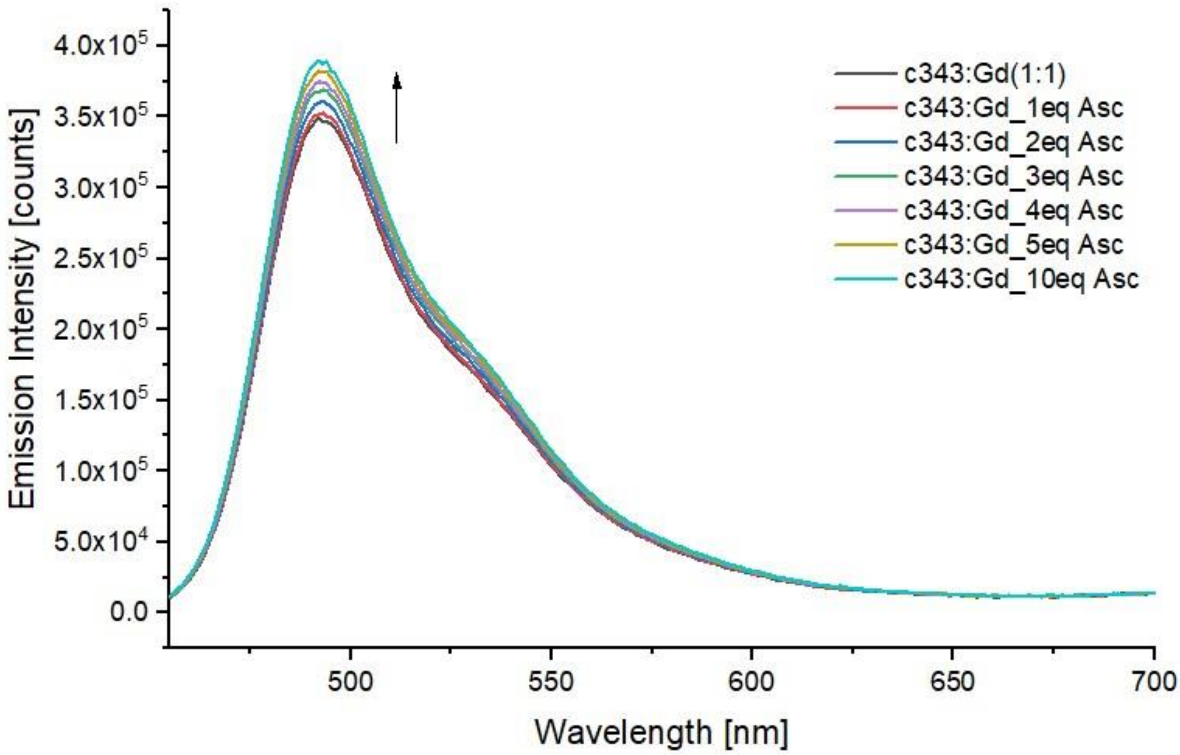

Figure S14. Fluorescence emission spectra of **C343** in a solution containing Gd(OTf)$_3$ (1 equiv.) in the presence of increasing amounts of ascorbic acid in MeCN:H_2O (4:1); λ_{ex} = 445 nm.

C343 fluorescent lifetimes and Ln(III) luminescent lifetimes

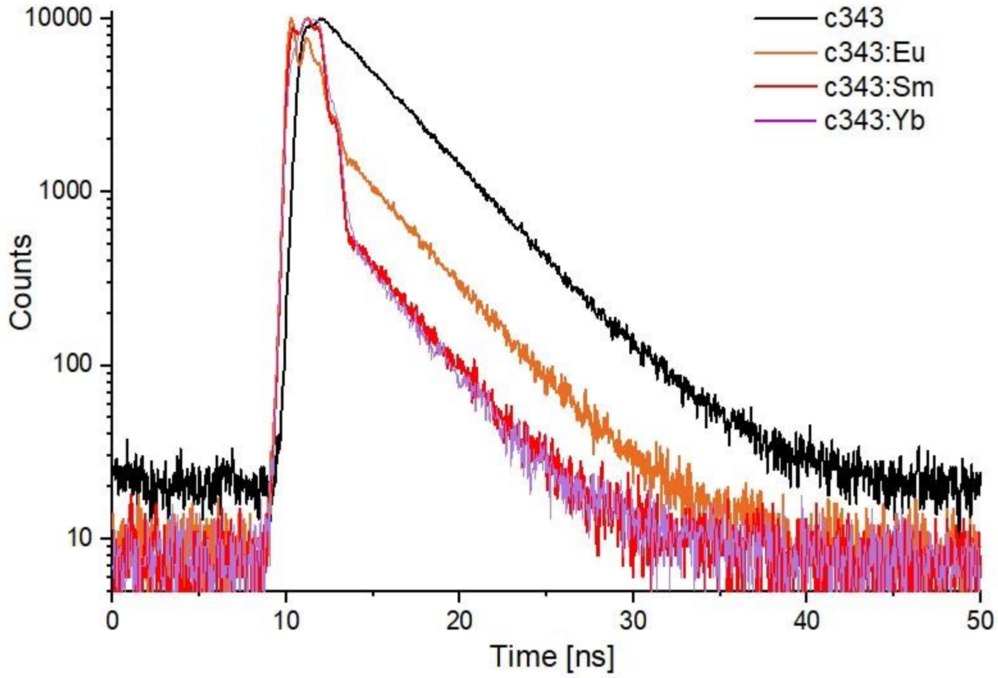

Figure S15. Luminescence decay of **C343** (black) and **C343** + Ln(OTf)₃ (1:1, Eu: orange, Sm: red, Yb: magenta) in MeCN, λ_{ex}= 470 nm.

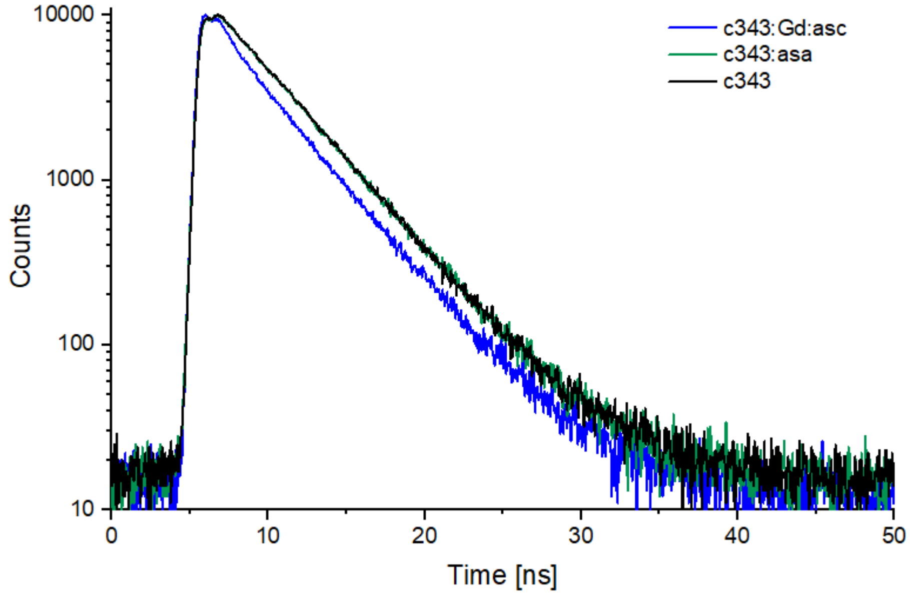

Figure S16. Luminescence decay of **C343** (black), **C343** + ascorbic acid (1:10, green), and **C343** + Gd(OTf)$_3$ + ascorbic acid (1:1:10, blue) in MeCN, λ_{ex}= 470 nm.

Table S3. Lifetimes ($\tau_{f,L}$) obtained by biexponential tail fit of **C343** and **LnC343** in MeCN; λ_{em} = 470 nm, λ_{em} = 492 nm. The χ^2 value is the goodness of fit (χ^2 = 1 is the best fit).

Compound	$\tau_{f,L}$ (ns)	χ^2
**C343**	3.99–4.00 ns	1.19
**C343** + Eu(OTf)$_3$	t_1 = 1.57 ns (54%), t_2 = 4.50 ns (46%)	17.1
**C343** + Sm(OTf)$_3$	t_1 = 0.76 ns (79%), t_2 = 4.51 ns (21%)	17.6
**C343** + Yb(OTf)$_3$	t_1 = 0.84 ns (90%), t_2 = 4.89 ns (10%)	20.2
**C343** + ascorbic acid	3.99 ns	1.20
**C343** + Gd(OTf)$_3$+ ascorbic acid	3.65 ns<br>t_1 = 3.10 ns (58%), t_2 = 4.50 ns (42%)	3.00<br>2.53

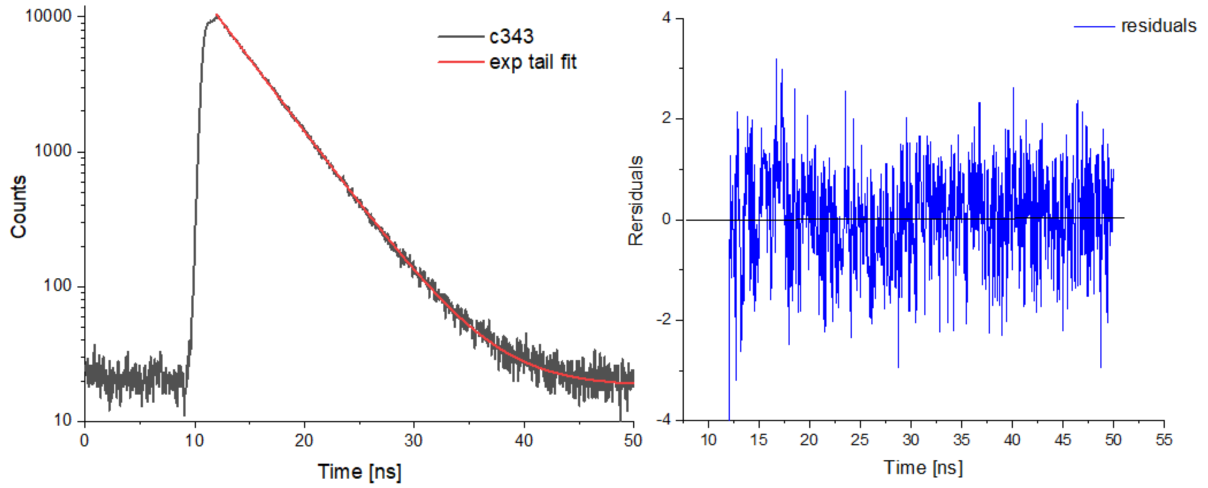

Figure S17. The fluorescence decay and biexponential tail fit of **C343** in MeCN (left) and residuals of the fit (right).

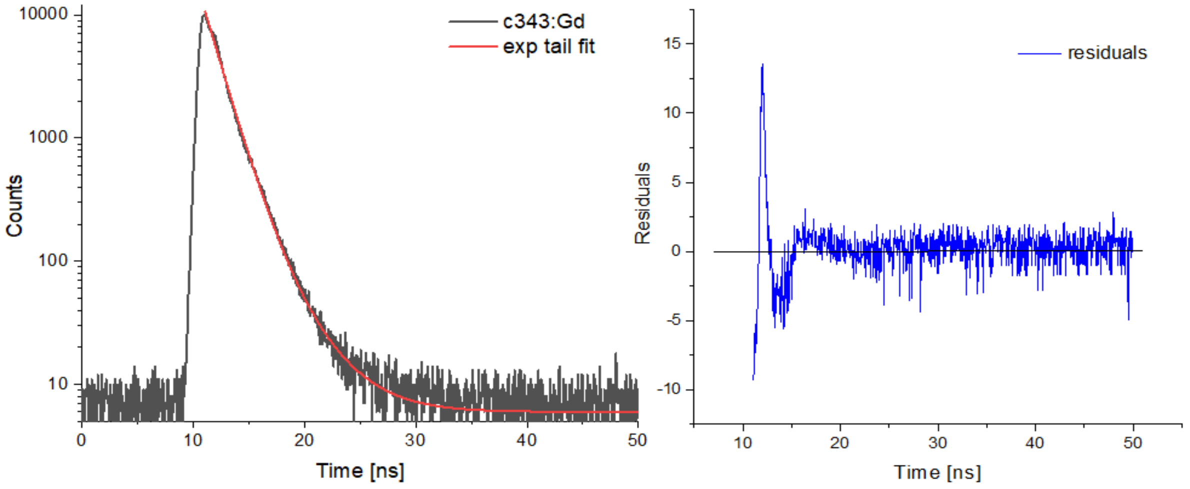

Figure S18. The fluorescence decay and biexponential tail fit of **C343**:Gd(OTf)$_3$ (1:1) in MeCN (left) and residuals of the fit (right).

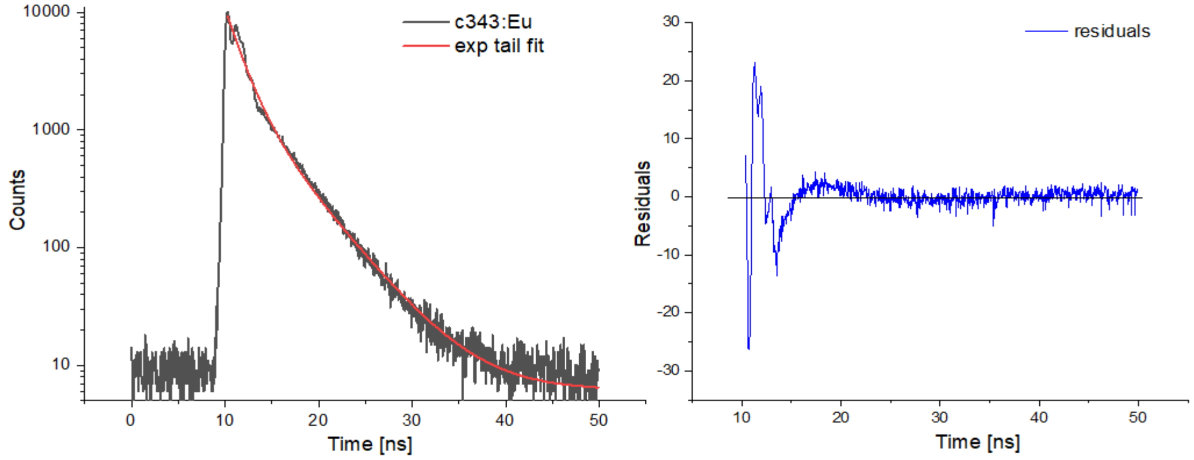

Figure S19. The fluorescence decay and biexponential tail fit of **C343**:Eu(OTf)$_3$ (1:1) in MeCN (left) and residuals of the fit (right).

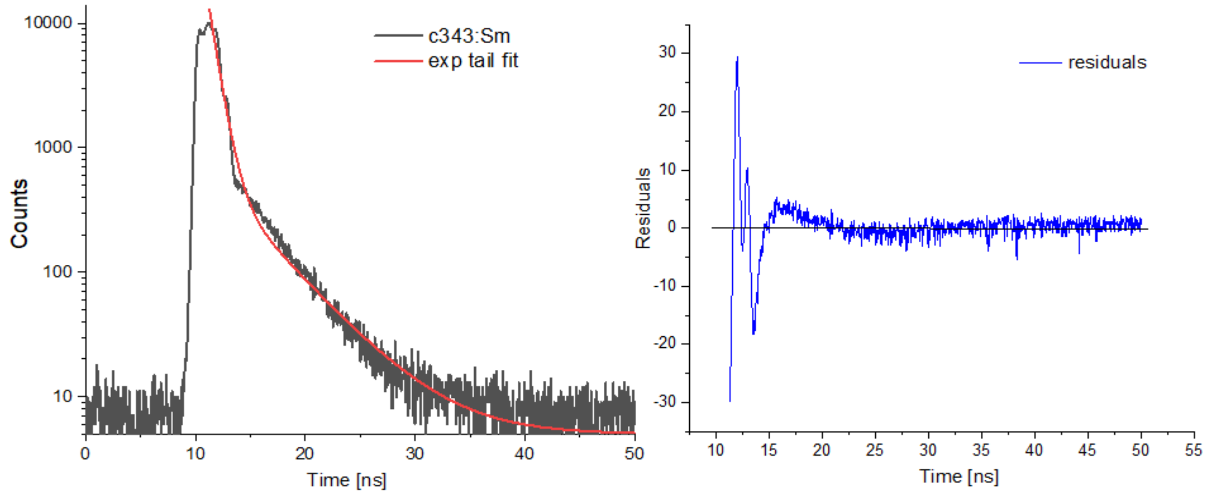

Figure S20. The fluorescence decay and biexponential tail fit of **C343**:Sm(OTf)$_3$ (1:1) in MeCN (left) and residuals of the fit (right).

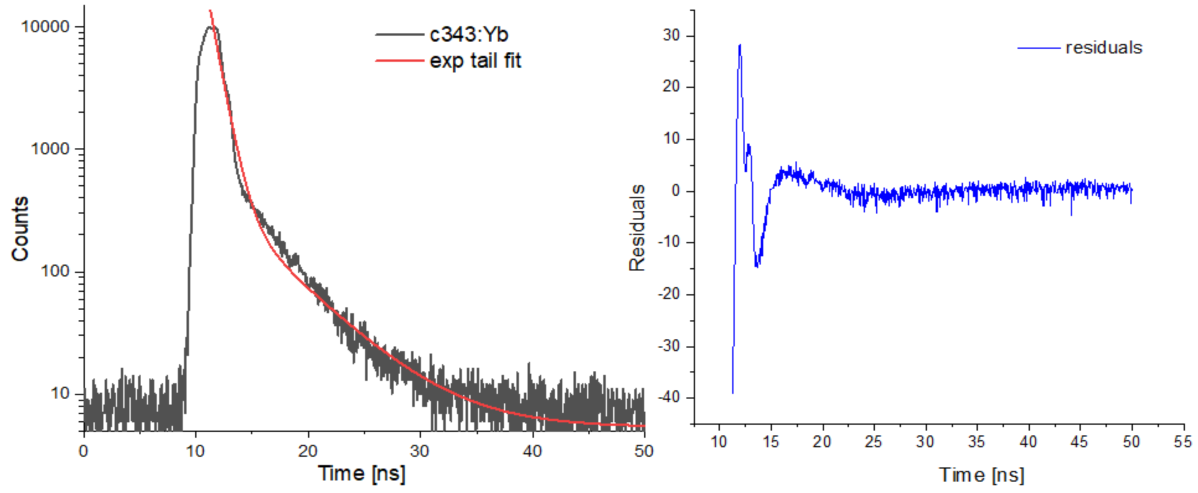

Figure S21. The fluorescence decay and biexponential tail fit of **C343**:Yb(OTf)$_3$ (1:1) in MeCN (left) and residuals of the fit (right).

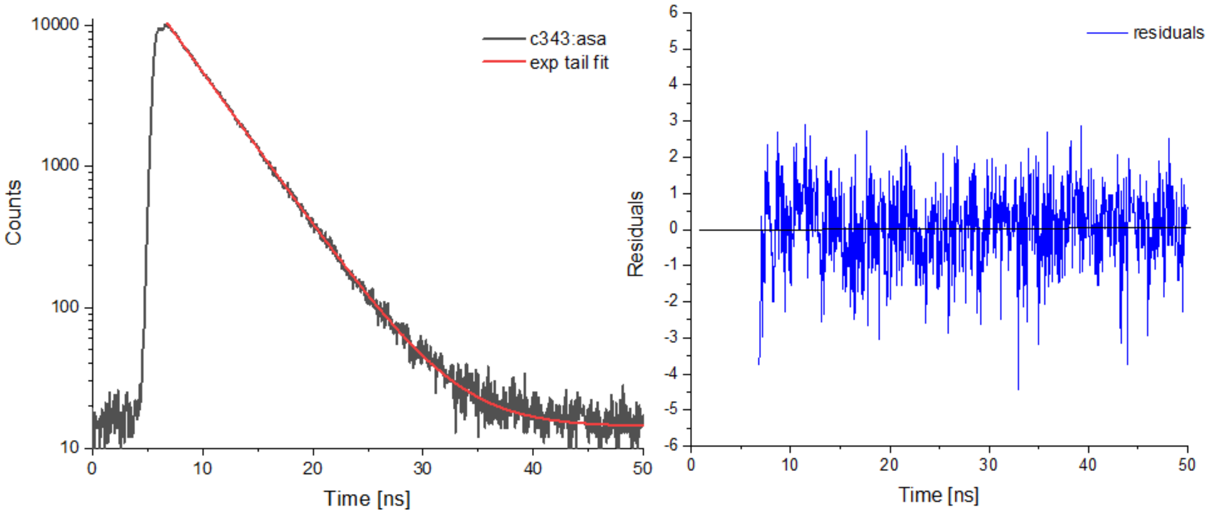

Figure S22. The fluorescence decay and biexponential tail fit of **C343**:ascorbic acid (1:10) in MeCN (left) and residuals of the fit (right).

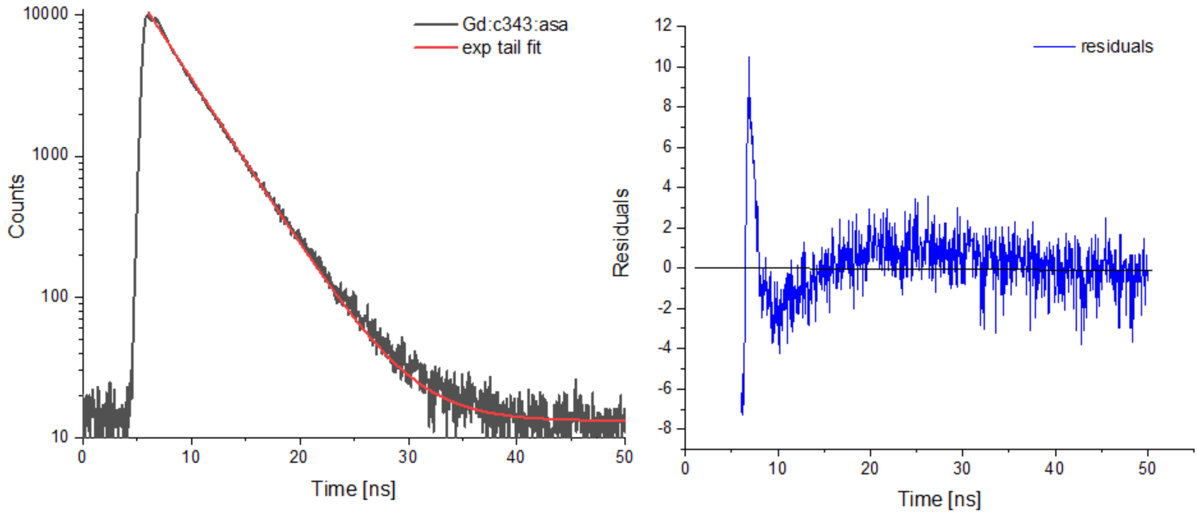

Figure S23. The fluorescence decay and monoexponential tail fit of **C343**:Gd(OTf)$_3$:ascorbic acid (1:1:10) in MeCN (left) and residuals of the fit (right).

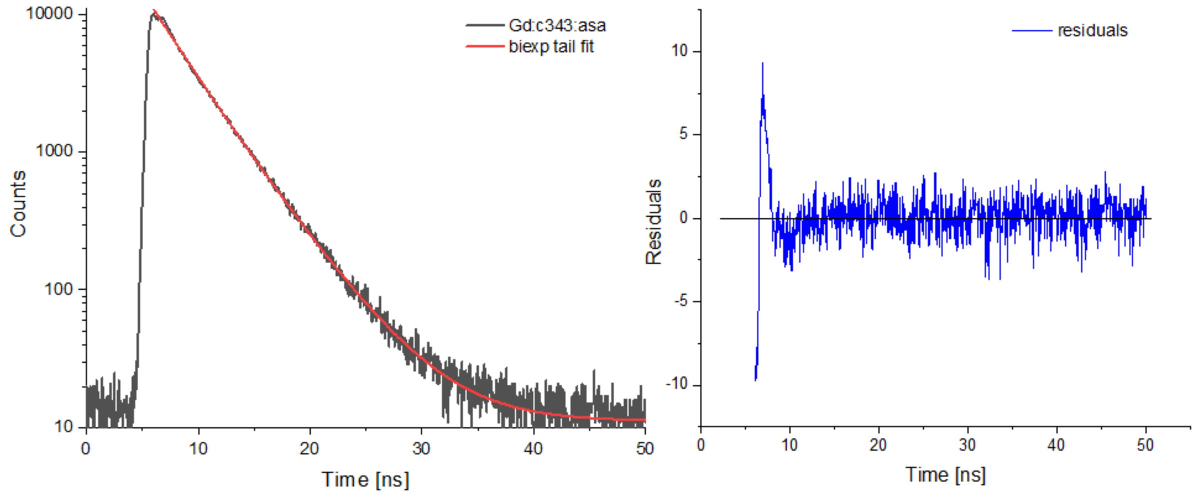

Figure S24. The fluorescence decay and biexponential tail fit of **C343**:Gd(OTf)$_3$:ascorbic acid (1:1:10) in MeCN (left) and residuals of the fit (right).

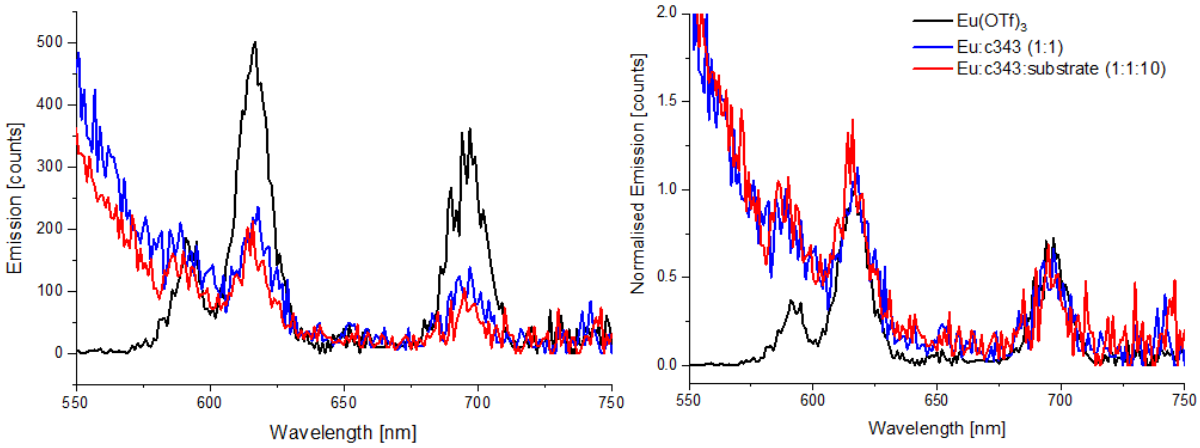

Figure S25. Time-resolved emission spectra (left) and normalized time-resolved emission spectra of Eu(OTf)$_3$ (black), Eu(OTf)$_3$ in the presence of **C343** (blue), and Eu(OTf)$_3$ in the presence of **C343** and trifluoromethyl benzaldehyde (red) in acetonitrile [Eu(OTf)$_3$] =17 µM, λ_{ex} = 393 nm; 0.05 ms time delay.

Table S4. Lifetimes of Eu(OTf)$_3$ in different conditions on direct excitation, λ_{ex} = 393 nm.

compound	τ_{Eu} (ms) in MeCN	τ_{Eu} (ms) in MeCN:H_2O (4:1)
Eu(OTf)$_3$	0.31	0.19
**EuC343**	0.24	0.083

5. Electrochemistry

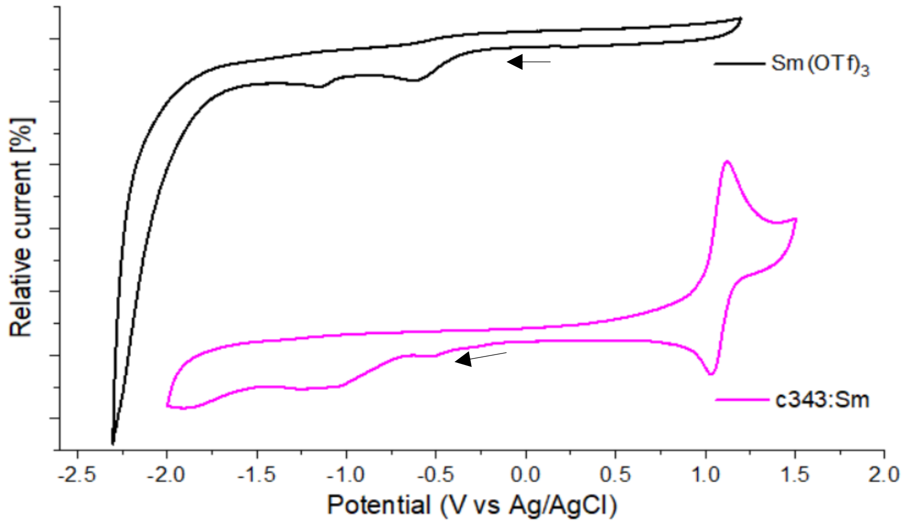

Figure S26. Cyclic voltammogram of Sm(OTf)$_3$ (black) and **C343** + Sm(OTf)$_3$ (1:1, pink), [**C343**] = [Sm(OTf)$_3$] = 5 mM, NBu$_4$PF$_6$ (0.1 M) in MeCN (5 mL) at room temperature; reference electrode, Ag/AgCl; working electrode, GC electrode; counter electrode, Pt wire; scan rate, 0.1 V/s (IUPAC convention), starting point is 0 towards the direction of the arrow.

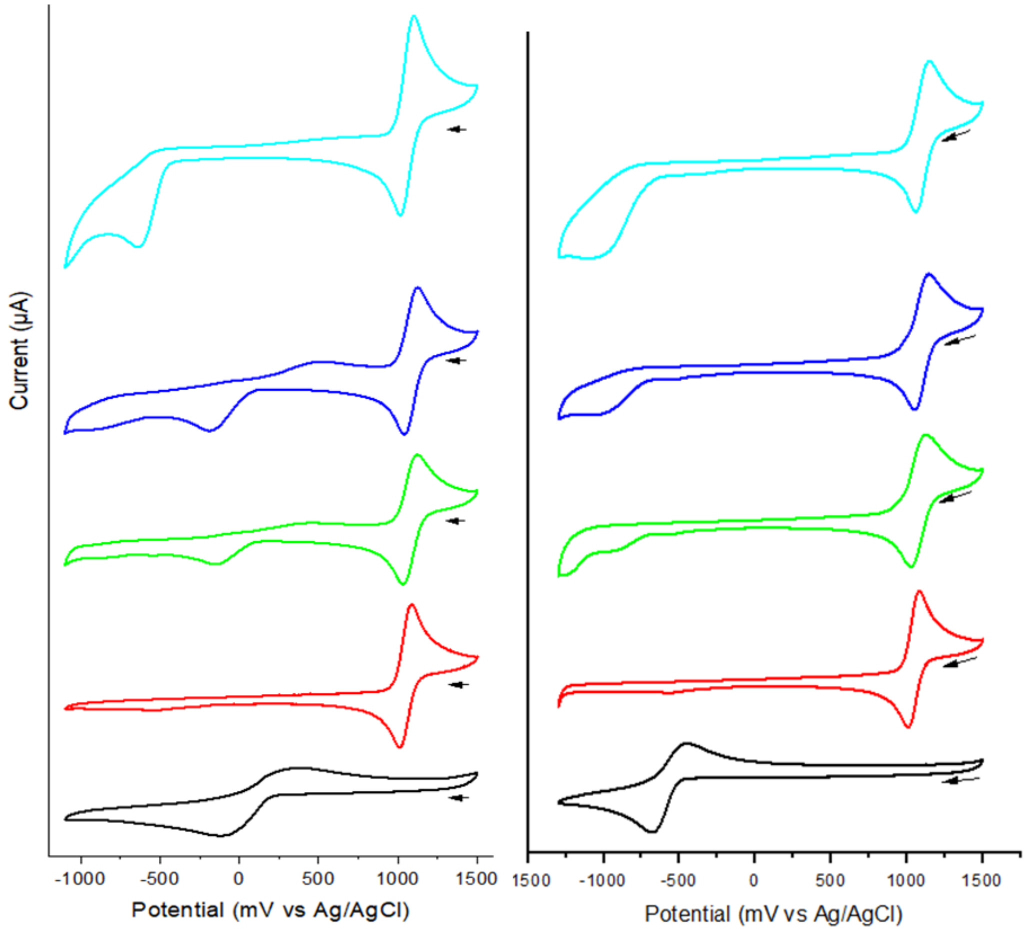

Figure S27. Cyclic voltammograms of **C343** + Eu(OTf)$_3$ (left side) and **C343** + Yb(OTf)$_3$ (right side) with different equivalents of Ln(OTf)$_3$ at room temperature (black: just Ln(OTf)$_3$; red: **C343**; green: 0.5 equiv.; blue: 1.0 equiv.; cyan: 2.0 equiv.), [**C343**] = [Ln(OTf)$_3$] = 5 mM, NBu$_4$PF$_6$ (0.1 M) in MeCN (5 mL); reference electrode, Ag/AgCl; working electrode, GC electrode; counter electrode, Pt wire; scan rate, 0.1 V/s (IUPAC convention), starting point is 0 towards the direction of the arrow.

Table S5: Calculated driving force for photoinduced electron transfer (ΔG_{PeT}) in MeCN (Ag/AgCl potential values converted to NHE).

Compound	E_{Ln}^{red} V NHE	ΔG_{PeT}
* $Eu(OTf)_3$	0.088	–1.57
**C343**:$Eu(OTf)_3$	–0.012	–1.47
* $Sm(OTf)_3$	–1.1	–0.4
**C343**:$Sm(OTf)_3$	–0.85	–0.63
* $Yb(OTf)_3$	–0.47	–1.01
**C343**:$Yb(OTf)_3$	–0.76	–0.72

***** E_{Ln}^{red} value without **C343**.

6. EPR Spectroscopy

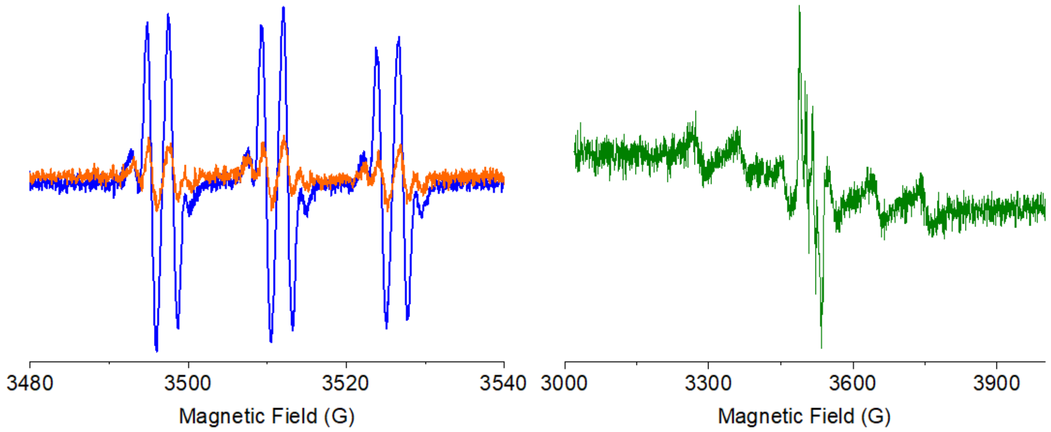

Figure S28. Left: EPR spectra of a mixture of **C343** and $Eu(OTf)_3$ (1:1, blue) or $Sm(OTf)_3$ (1:1, orange) and PBN in DMF after 12 h irradiation at room temperature. T = 293 K, microwave power: 2mW, Modulation amplitude: 1 G. Right: EPR spectrum of a mixture of **C343** and $Eu(OTf)_3$ (1:1, green) showing the wider Eu(II) signal. The central sharper signal is the N-based radical signal. T = 293 K, microwave power: 2 mW, modulation amplitude: 20 G.

7. GC-MS traces

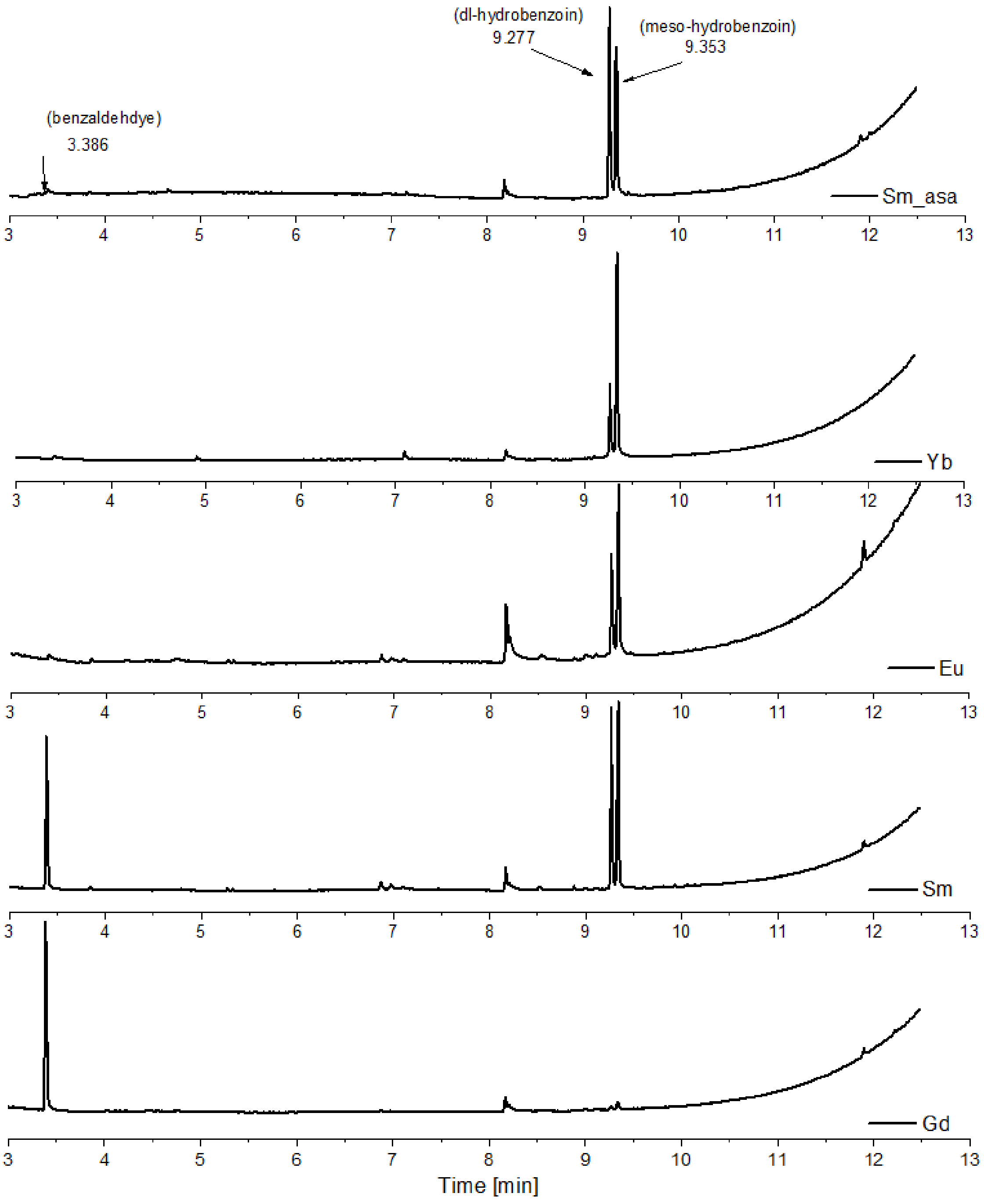

Figure S29. Typical GC-MS trace of the products of **1a**.

8. X-ray crystallography

Single crystals were mounted on a fiber loop and fixated using Fomblin oil. The data were collected on a Bruker SMART APEX2 area detector using MoK□ radiation (□ = 0.71073 Å) at ambient temperature. Data reduction was performed with SAINT, absorption corrections for the area detector were performed using SADABS (2016/2). Structures were solved by direct methods and refined by least squares methods on F2 using the SHELX and the OLEX2 software suites, respectively. All the non-hydrogen atoms were refined using an anisotropic model and all the hydrogen atoms were constrained in geometrical positions to their parent atom. A solvent DMSO solvent molecule was modelled with a positional disorder and site occupancy factors of 0.64:0.36. Crystallographic data are presented in **Table S6**. Deposition number 2386770 contains the supplementary crystallographic data for this paper. These data can be obtained free of charge via www.ccdc.cam.ac.uk/data_request/cif, or by emailing data_request@ccdc.cam.ac.uk, or by contacting The Cambridge Crystallographic Data Centre, 12 Union Road, Cambridge CB21EZ, UK; fax:+441223336033.

Table S6. Crystal data and structure refinement for **21c**.

Identification code CCDC	2386770
Empirical formula	$C_{28}H_{26}F_2O_5S$
Formula weight	512.55
Temperature/K	293.15
Crystal system	monoclinic
Space group	$P2_1/n$
a/Å	9.719(3)
b/Å	11.782(4)
c/Å	21.830(7)
α/°	90
β/°	78.320(7)
γ/°	90
Volume/Å^3	2447.9(13)
Z	4
ρ_{calc}g/cm^3	1.391
μ/mm^{-1}	0.186
F(000)	1072.0
Crystal size/mm^3	0.12 × 0.11 × 0.05
Radiation	MoKα (λ = 0.71073)
2Θ range for data collection/°	5.146 to 50.786
Index ranges	$-11 \leq h \leq 11, -14 \leq k \leq 14, -26 \leq l \leq 26$
Reflections collected	39473
Independent reflections	4458 [R_{int} = 0.1483, R_{sigma} = 0.1471]
Data/restraints/parameters	4458/67/368
Goodness-of-fit on F^2	1.014
Final R indexes [I>=2σ (I)]	R_1 = 0.0735, wR_2 = 0.1708
Final R indexes [all data]	R_1 = 0.2334, wR_2 = 0.2293
Largest diff. peak/hole / e Å^{-3}	0.22/–0.25

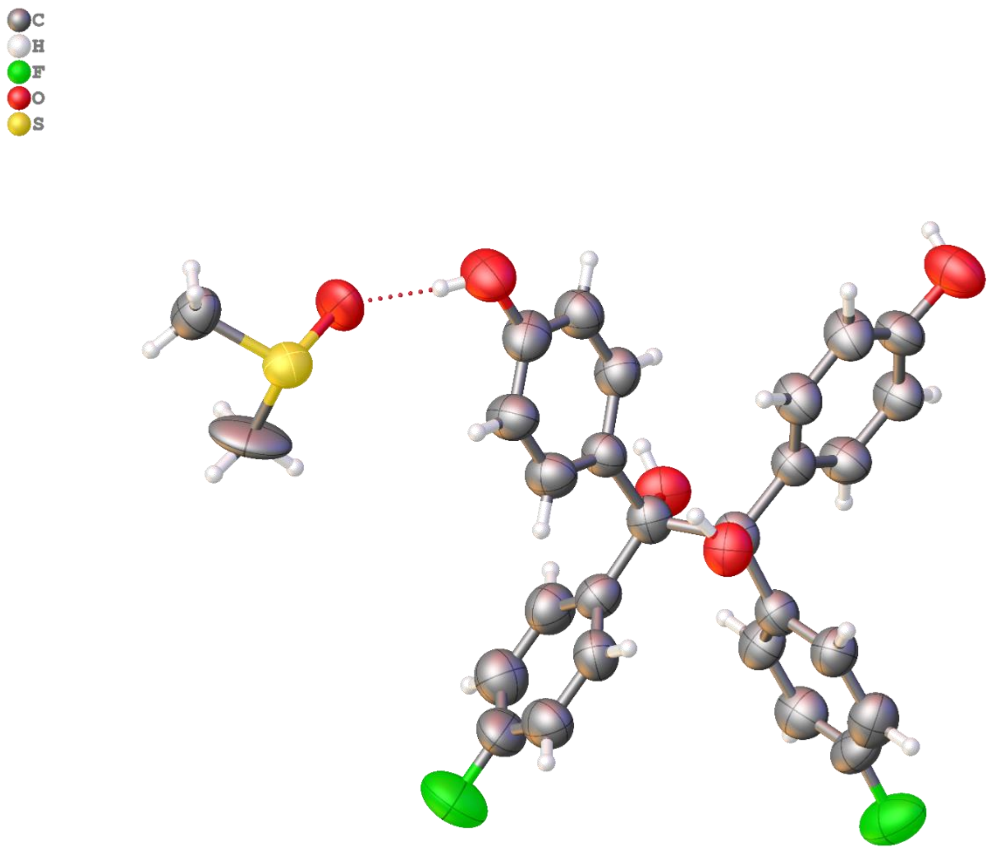

Figure S30. ORTEP representation of the solid-state structure of compound **21c**. Ellipsoids are set at a 50% probability level.

9. ^{1}H, ^{13}C and ^{19}F NMR spectra

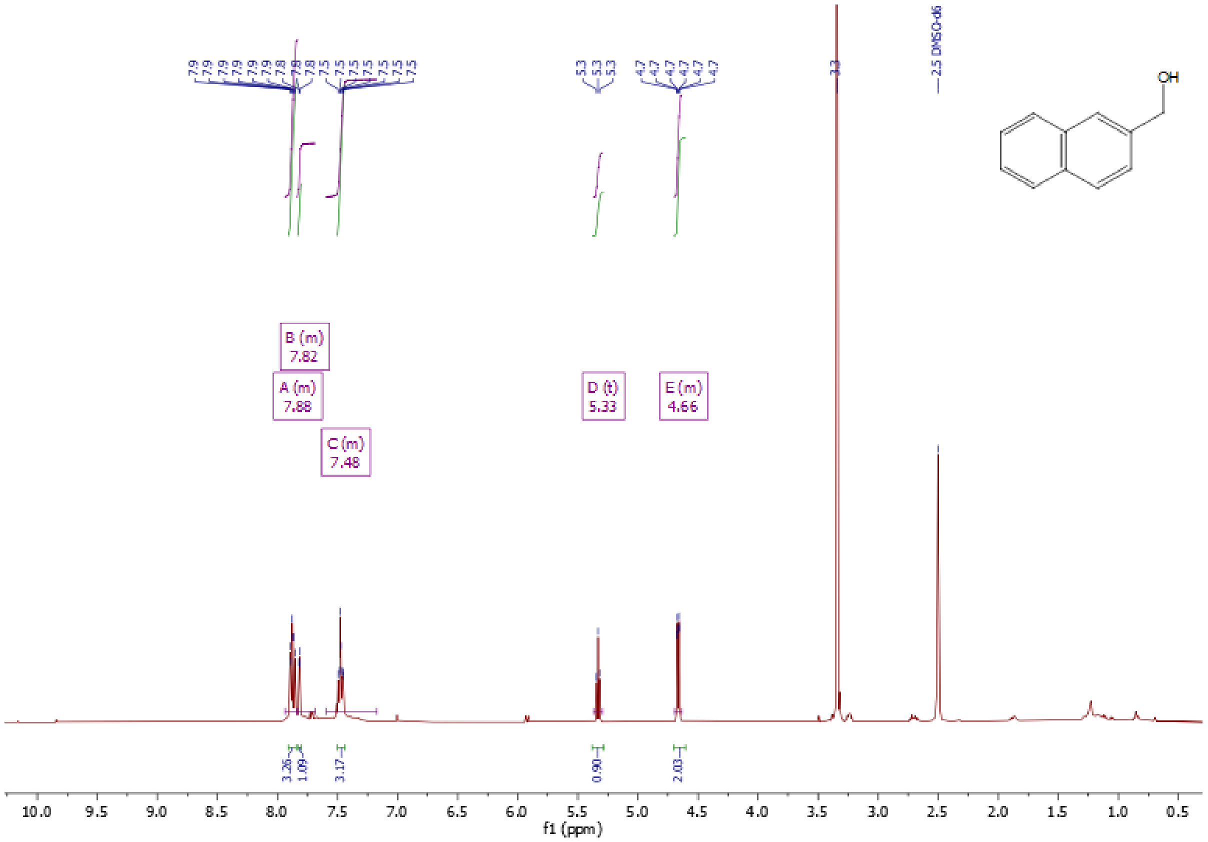

Figure S31. ^{1}H NMR spectrum of **2b** in DMSO-d_6.

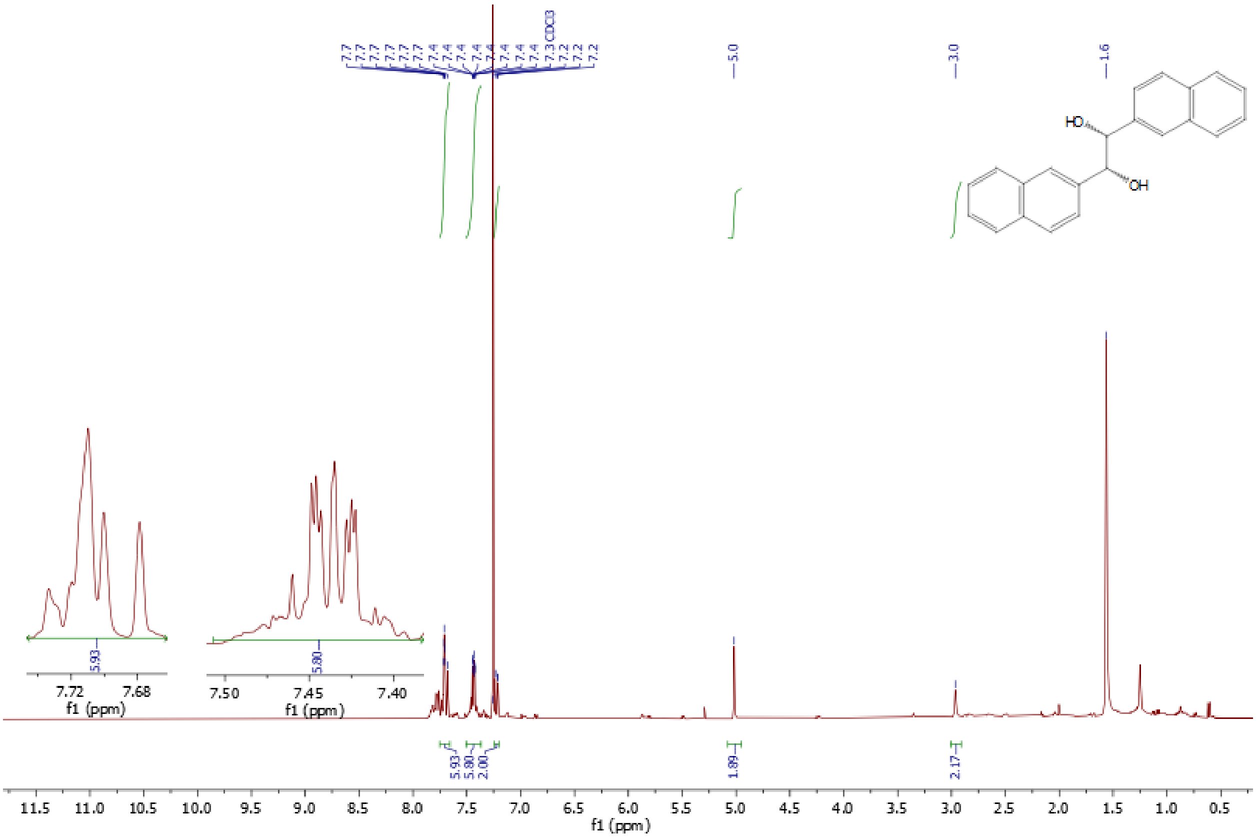

Figure S32. ^{1}H NMR spectrum of **2c** in $CDCl_3$.

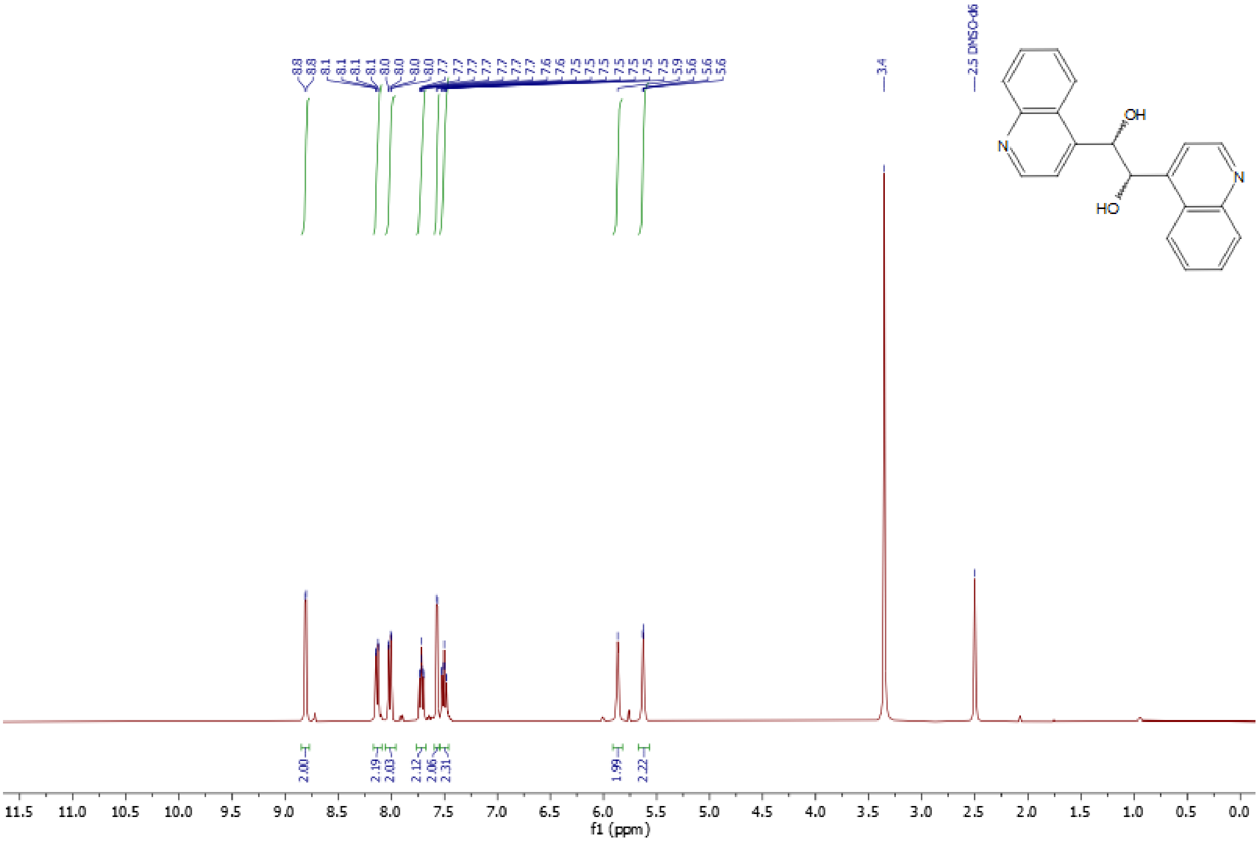

Figure S33. ^{1}H NMR spectrum of **3c** in DMSO-d_6.

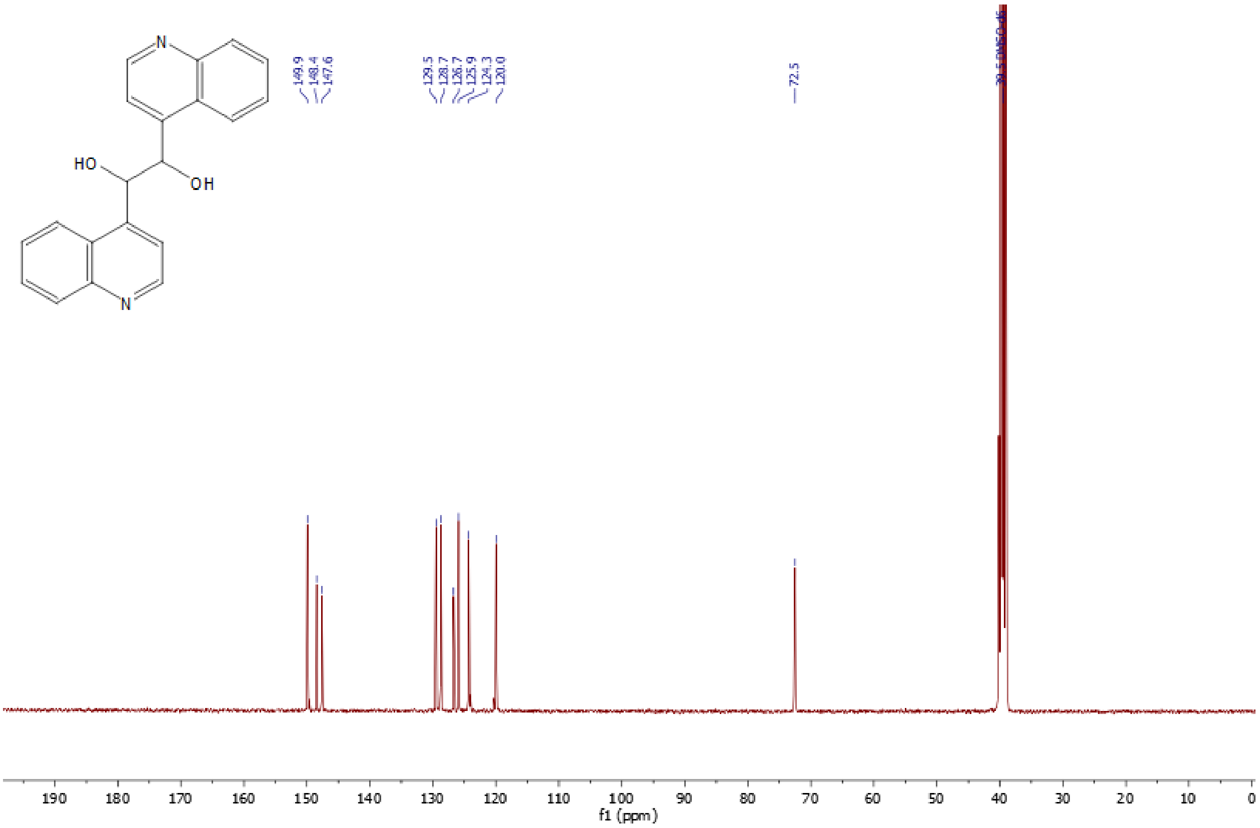

Figure S34. ^{13}C NMR spectrum of **3c** in DMSO-d_6.

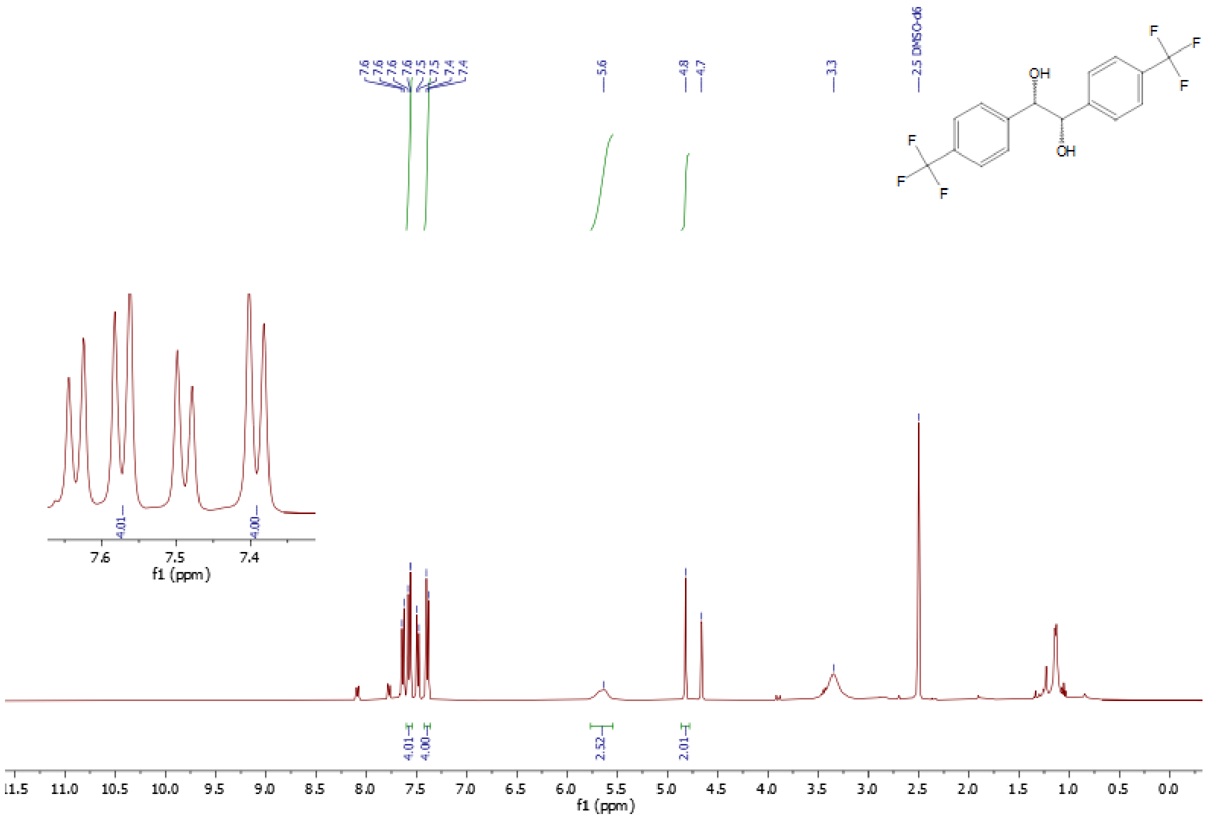

Figure S35. ^{1}H NMR spectrum of **4c** in DMSO-d_6.

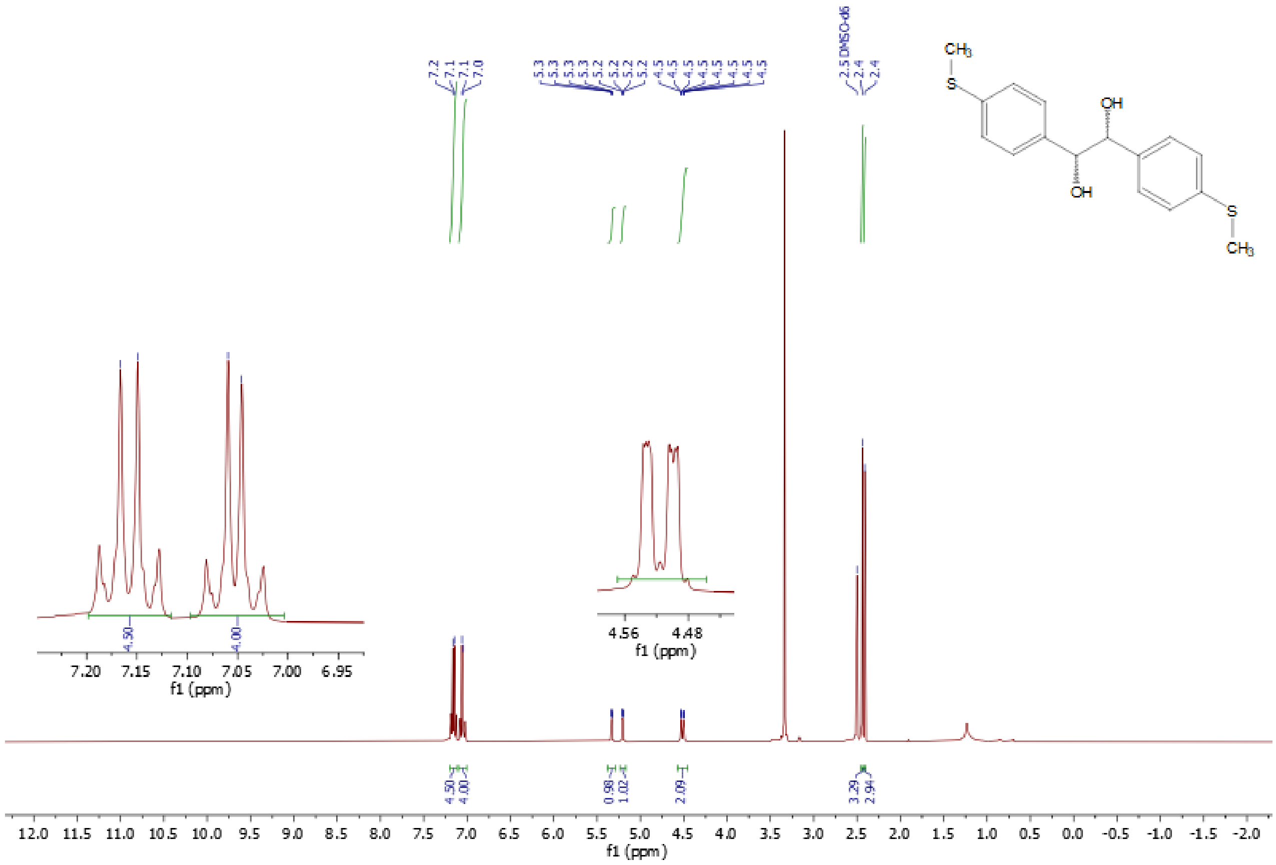

Figure S36. ^{1}H NMR spectrum of **5c** in DMSO-d_6.

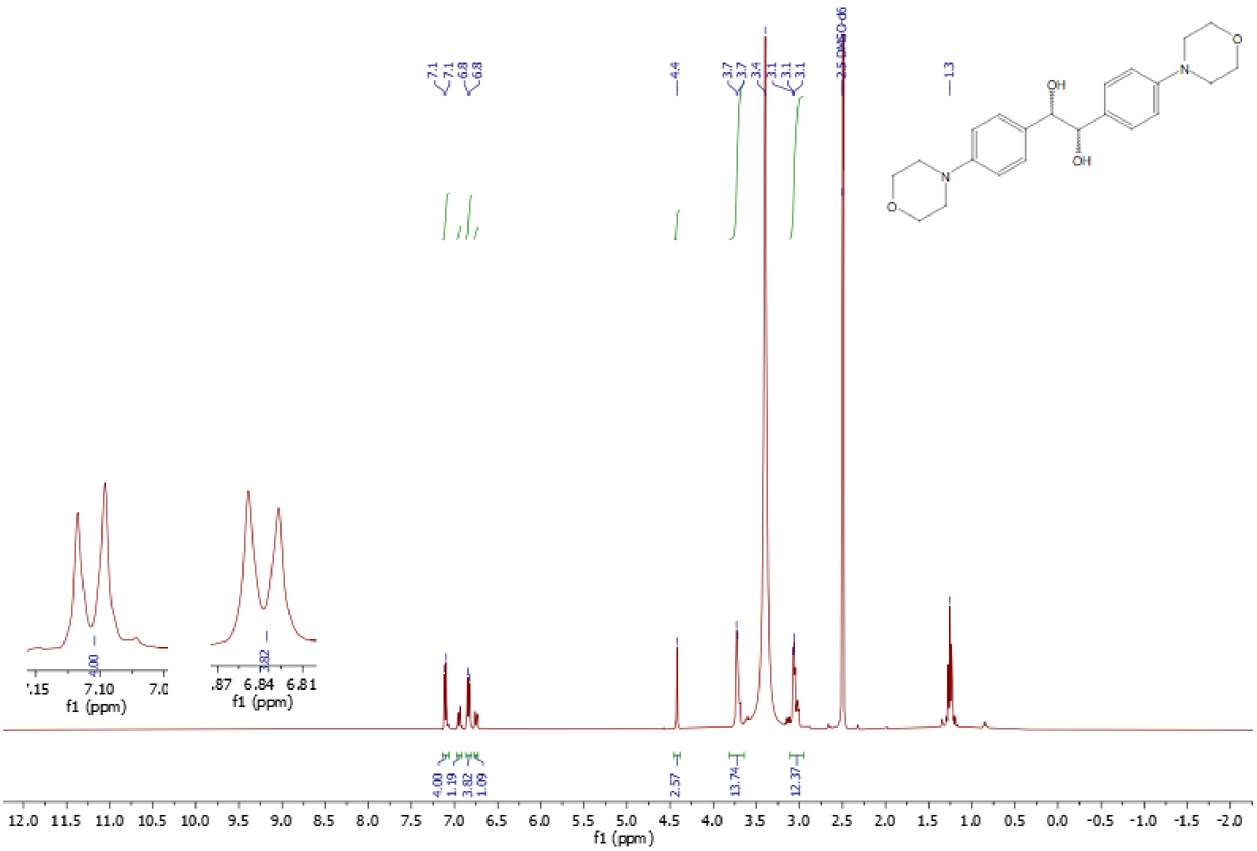

Figure S37. ^{1}H NMR spectrum of **6c** in DMSO-d_6.

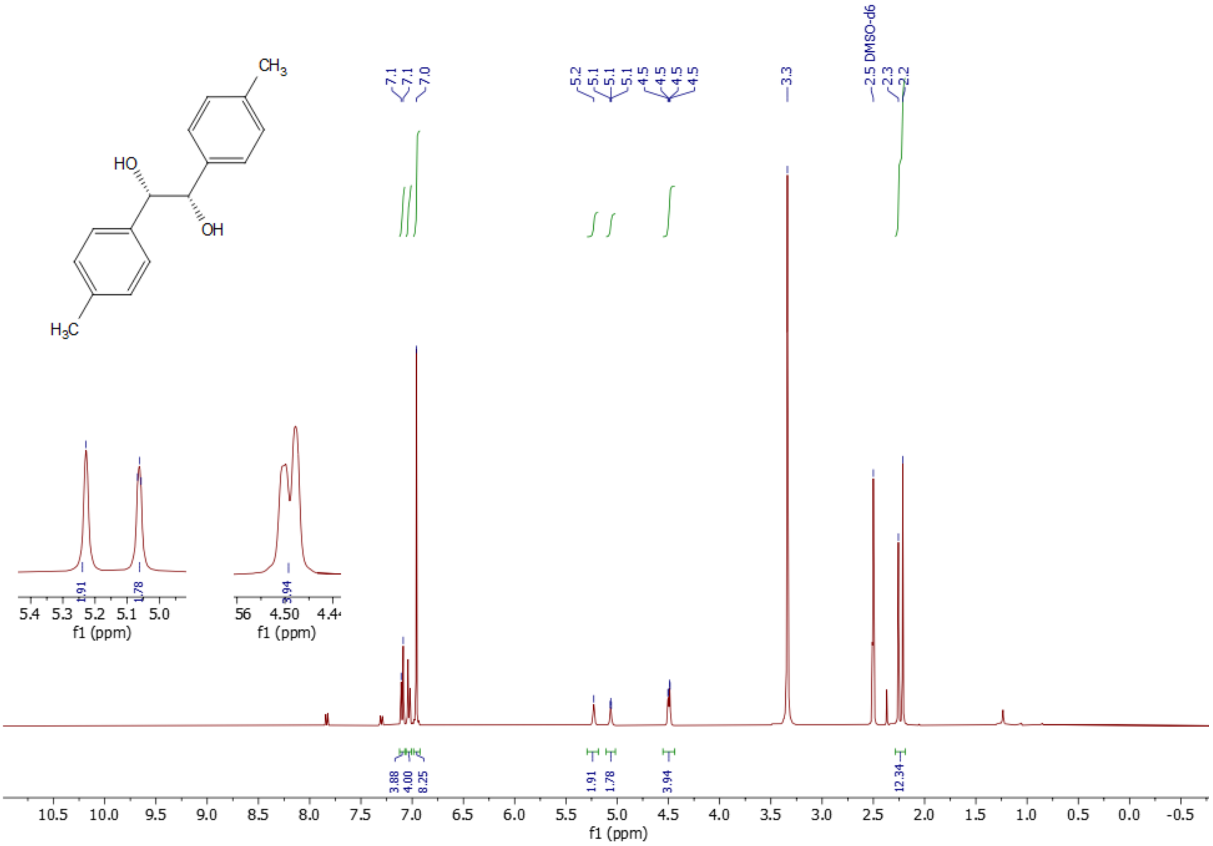

Figure S38. ^{1}H NMR spectrum of **7c** in DMSO-d_6.

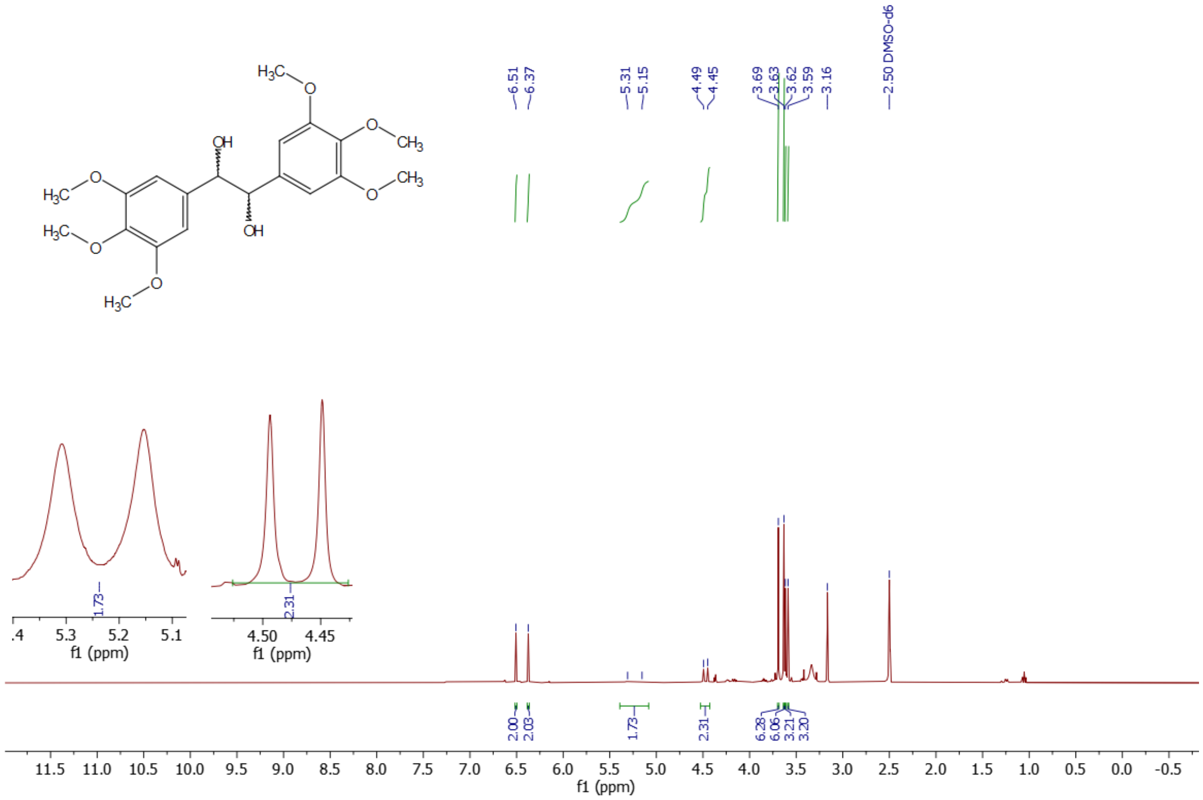

Figure S39. ^{1}H NMR spectrum of **8c** in DMSO-d_6.

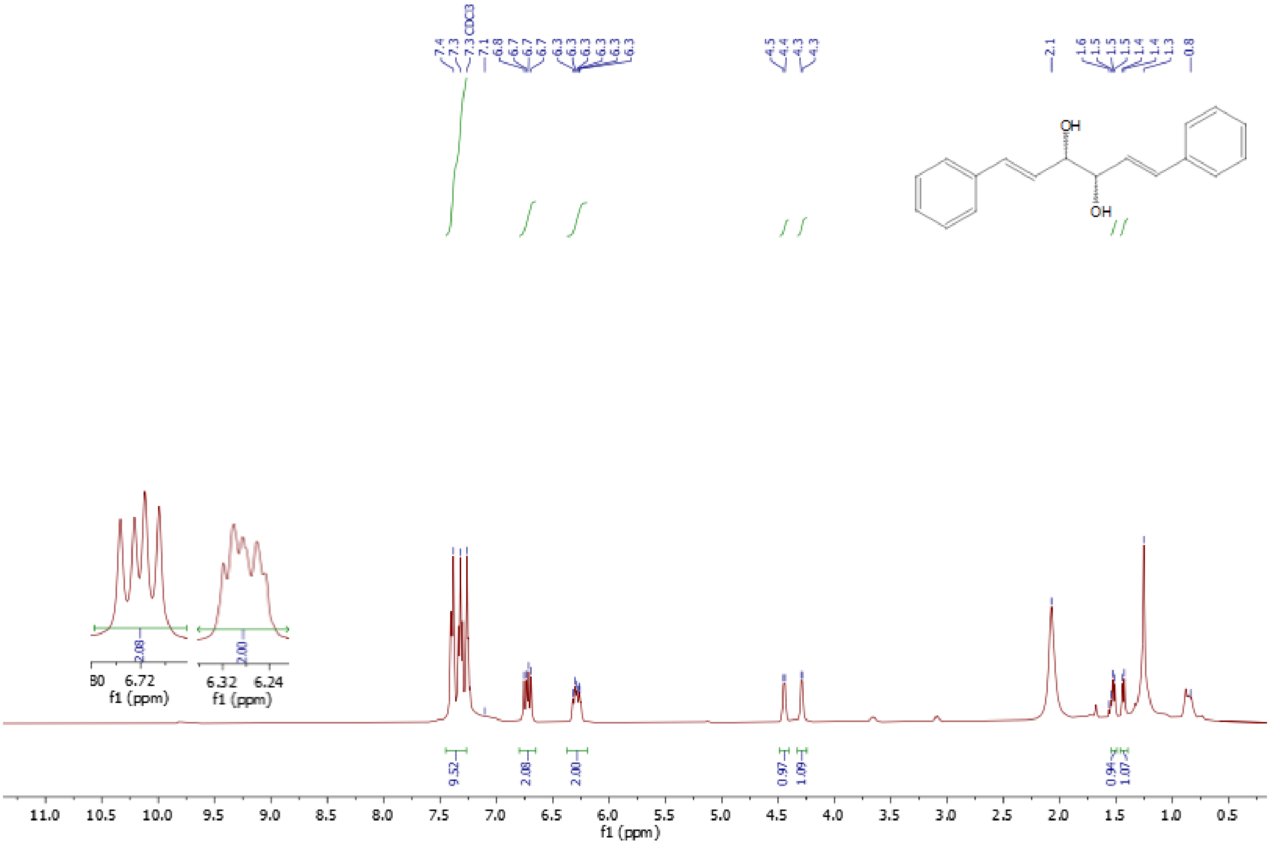

Figure S40. ^{1}H NMR spectrum of **9c** in $CDCl_3$.

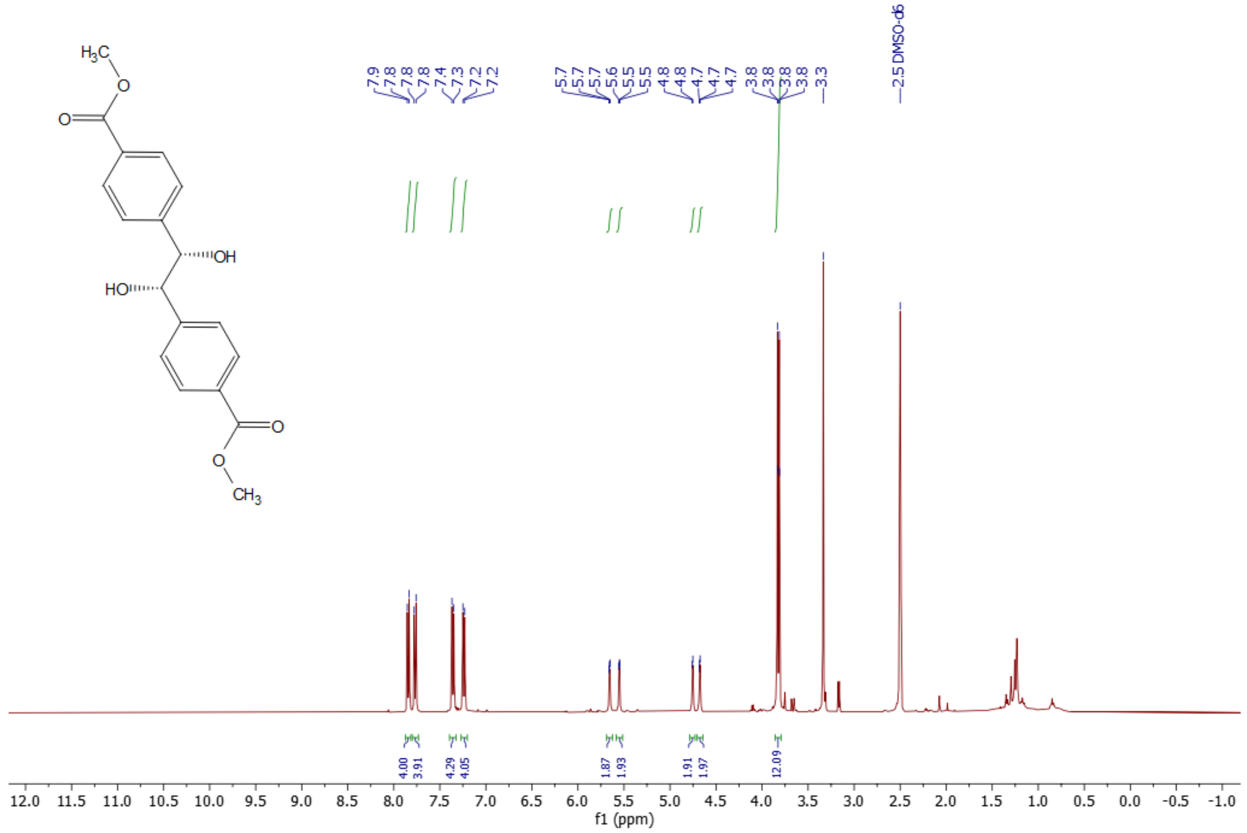

Figure S41. ^{1}H NMR spectrum of **10c** in DMSO-d_6.

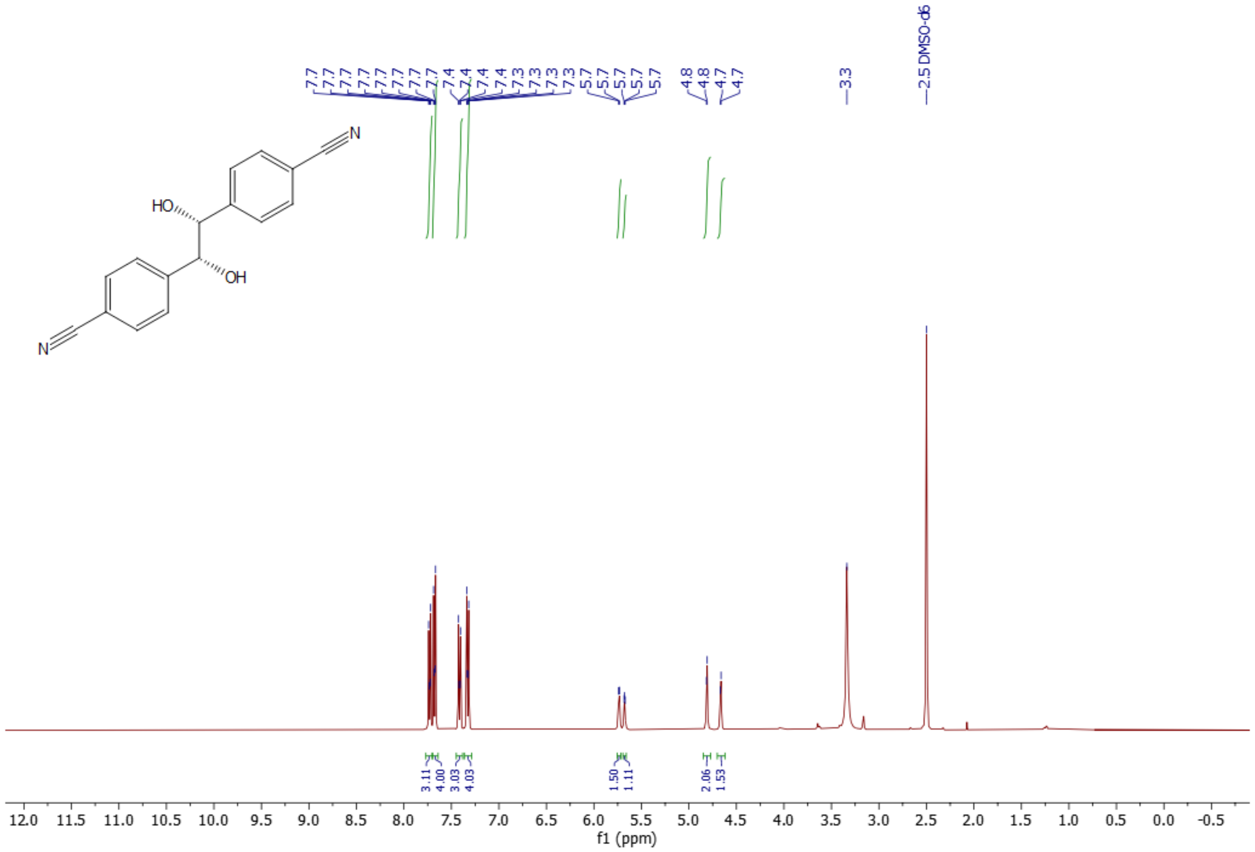

Figure S42. ^{1}H NMR spectrum of **11c** in DMSO-d_6.

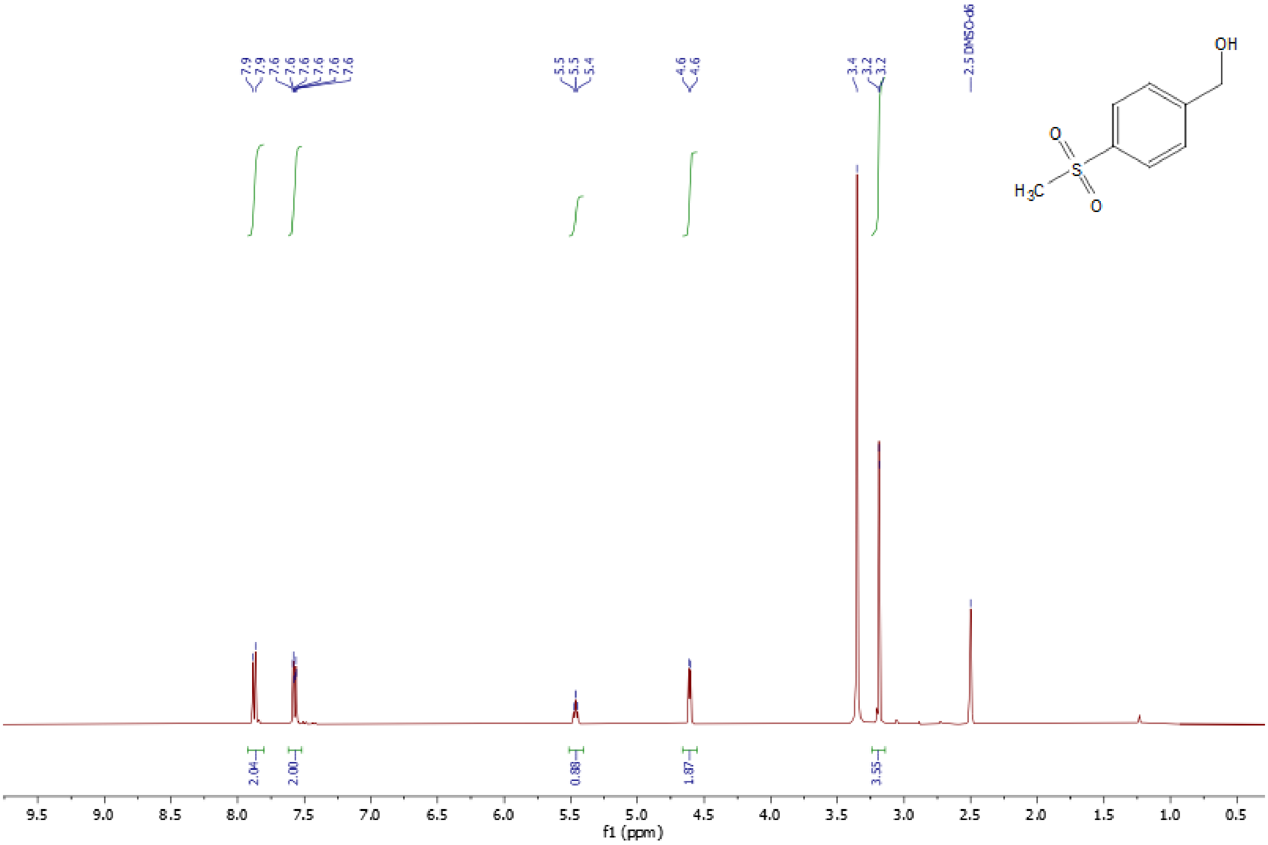

Figure S43. ^{1}H NMR spectrum of **12b** in DMSO-d_6.

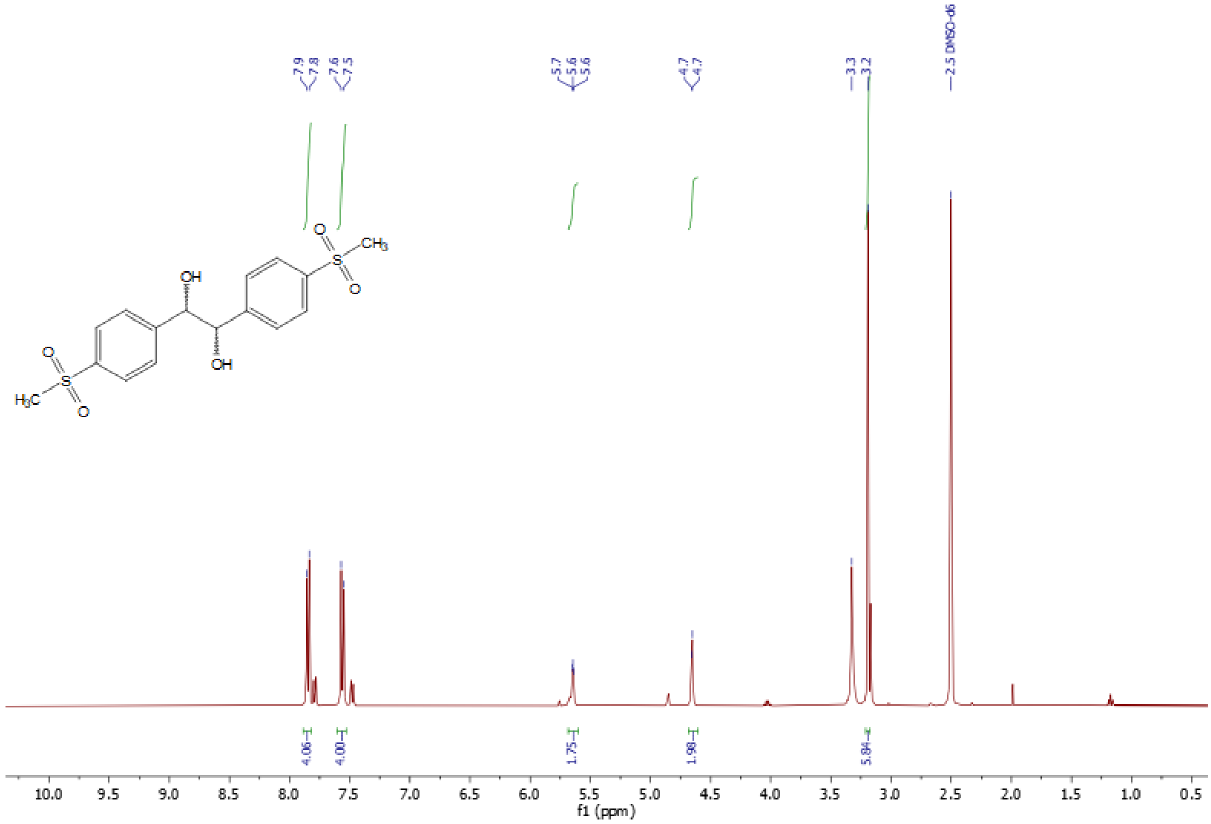

Figure S44. ^{1}H NMR spectrum of **12c** in DMSO-d_6.

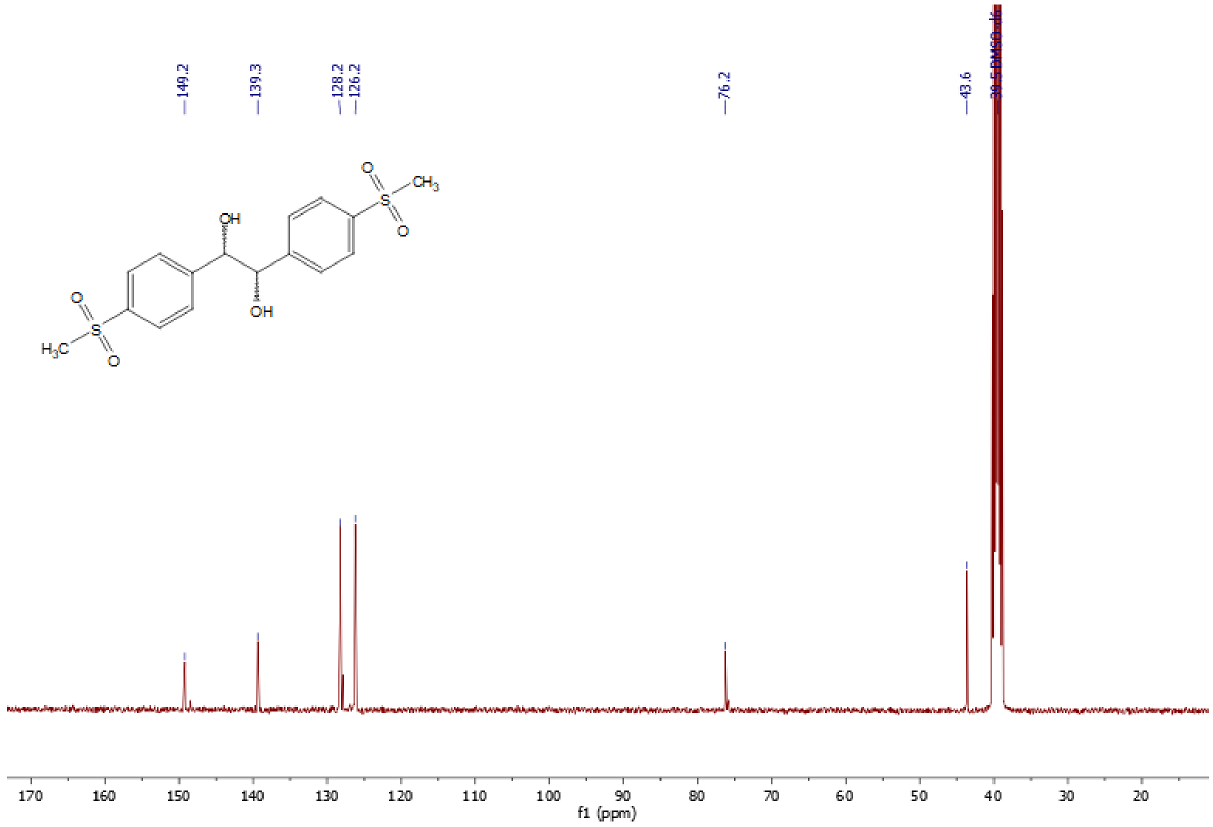

Figure S45. ^{13}C NMR spectrum of **12c** in DMSO-d_6.

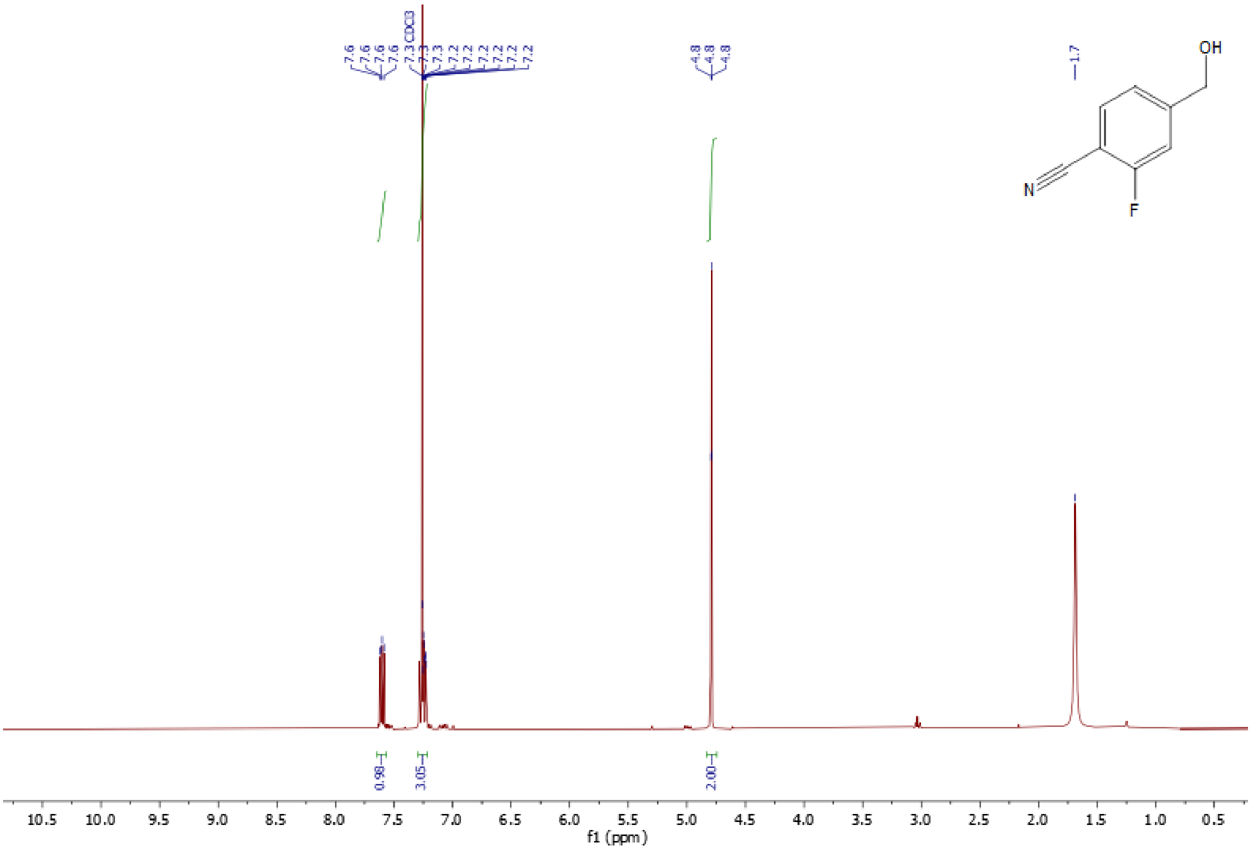

Figure S46. ^{1}H NMR spectrum of **13b** in $CDCl_3$.

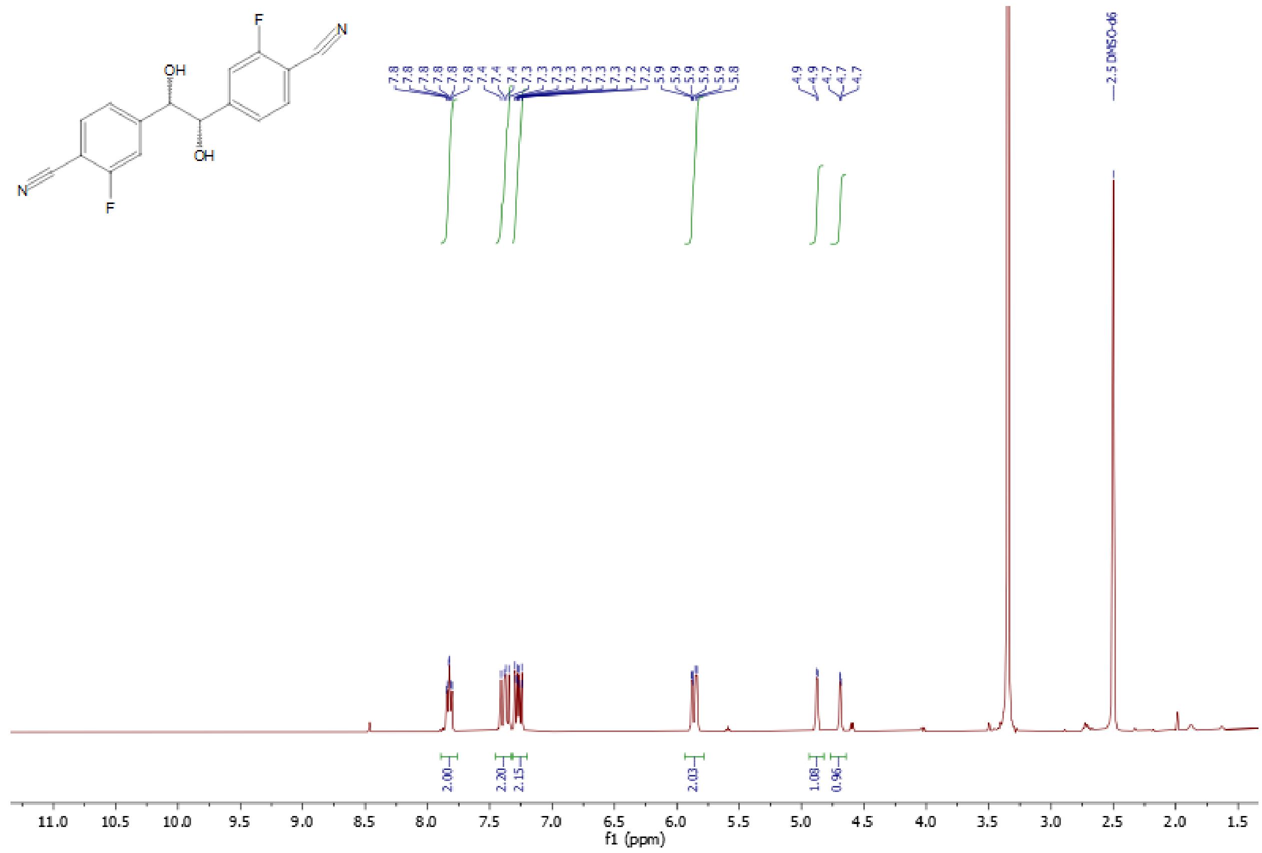

Figure S47. ^{1}H NMR spectrum of **13c** in DMSO-d_6.

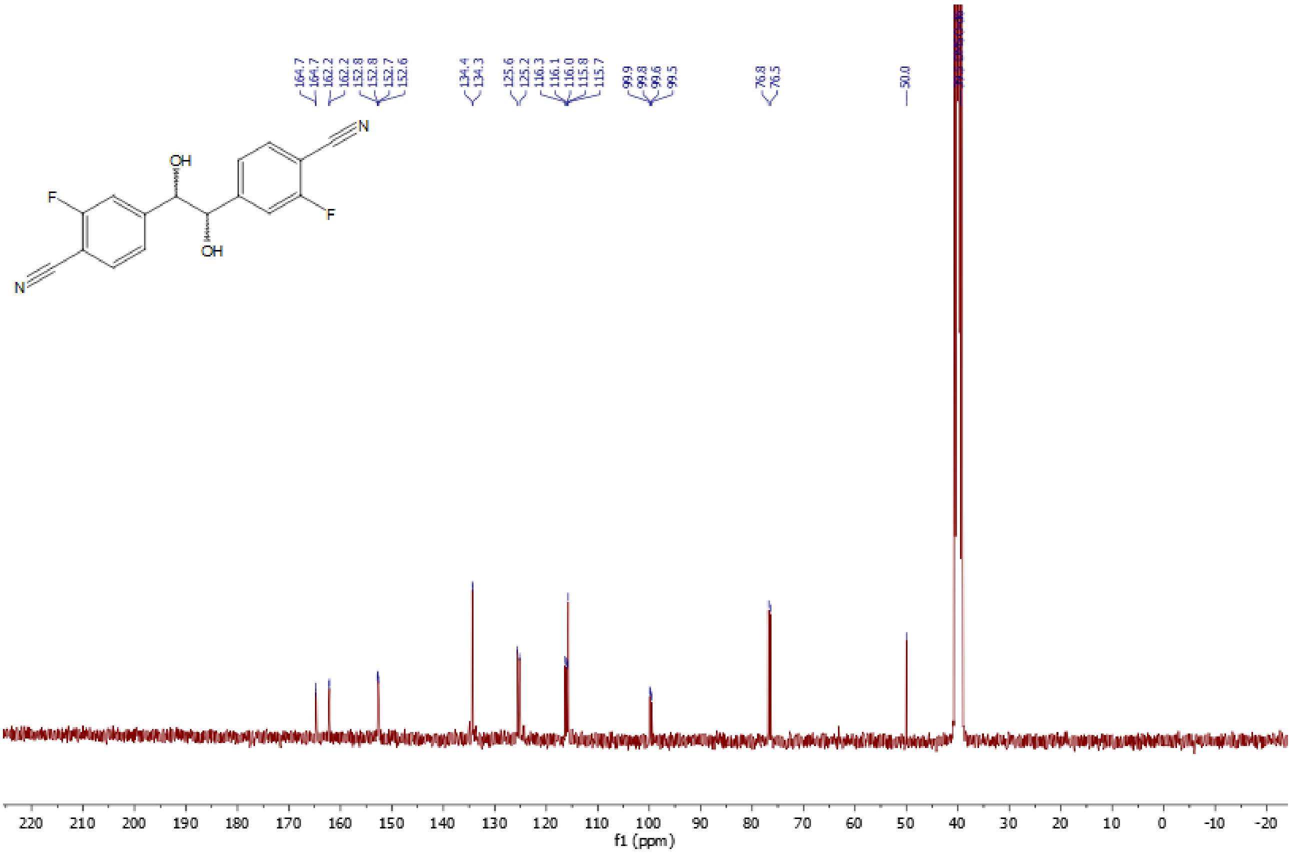

Figure S48. ^{13}C NMR spectrum of **13c** in DMSO-d_6.

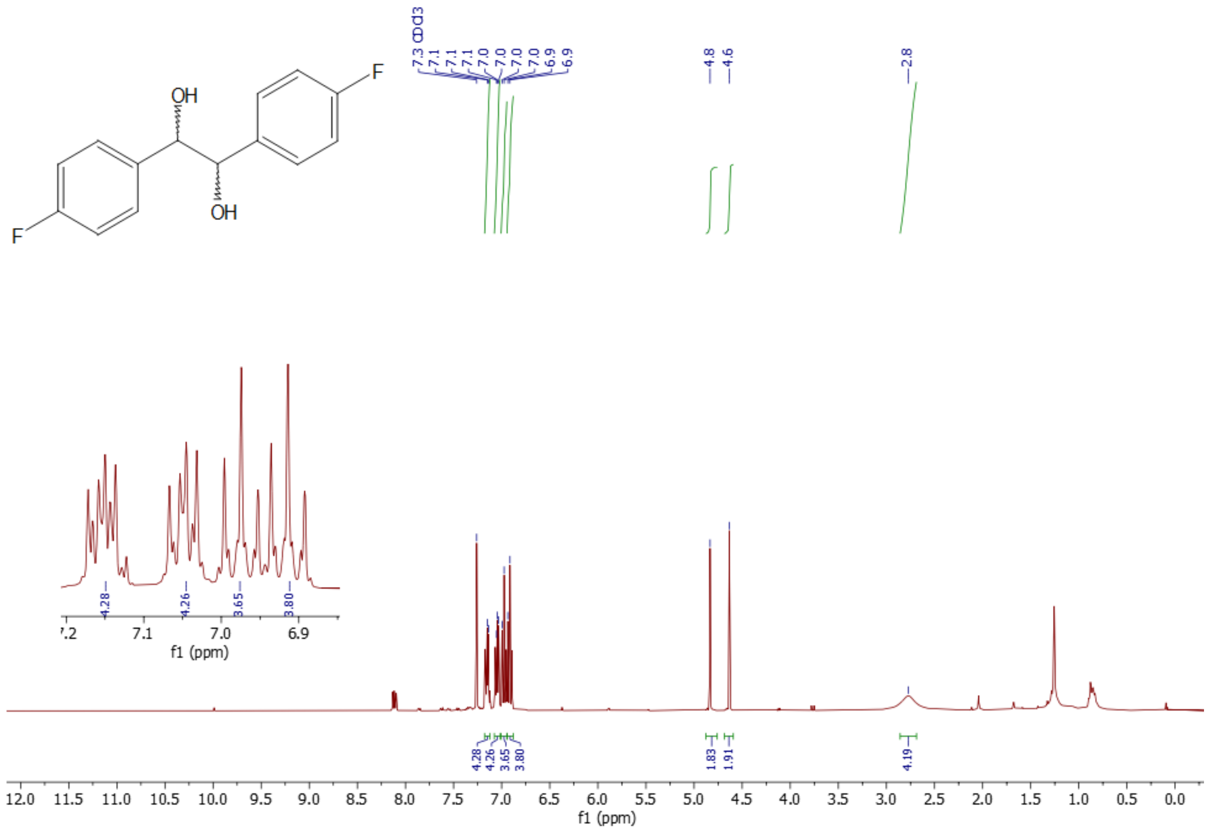

Figure S49. ^{1}H NMR spectrum of **14c** in $CDCl_3$.

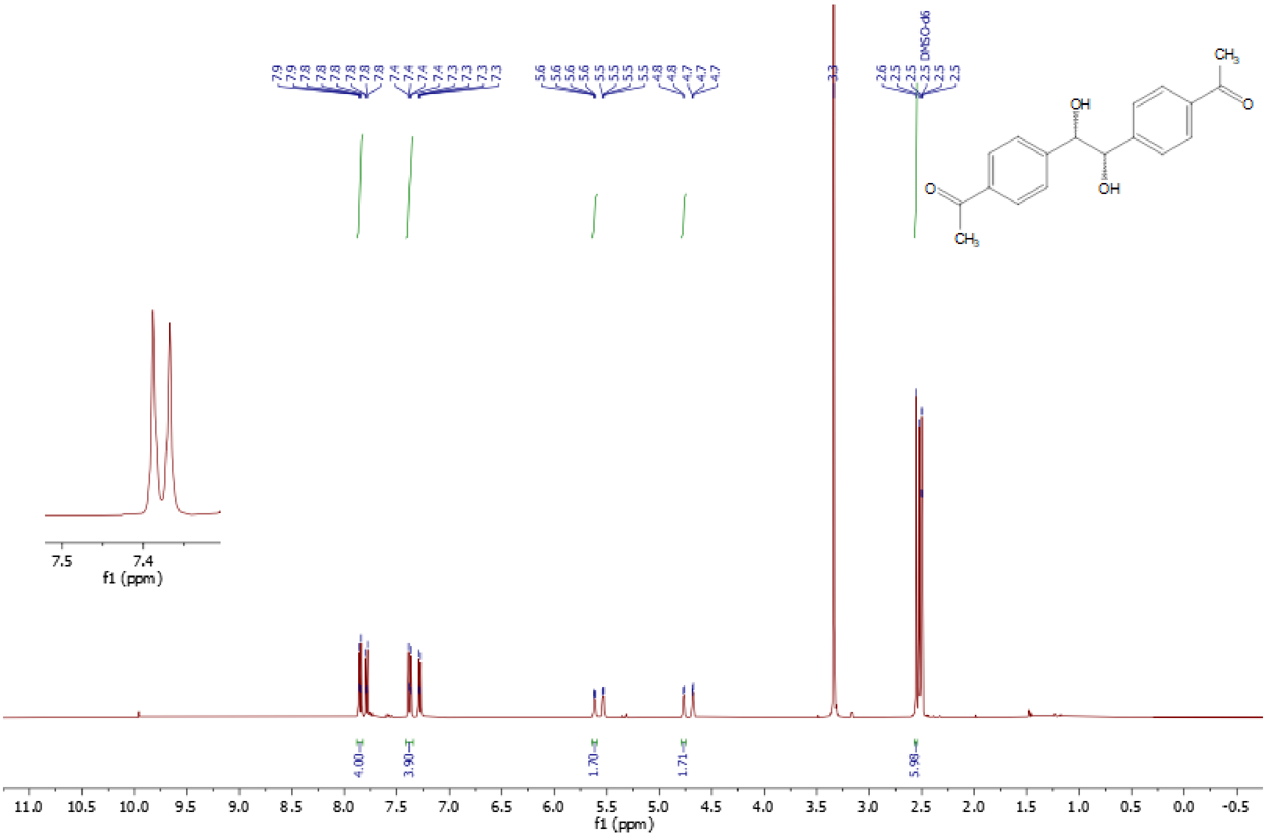

Figure S50. ^{1}H NMR spectrum of **15c** in DMSO-d_6.

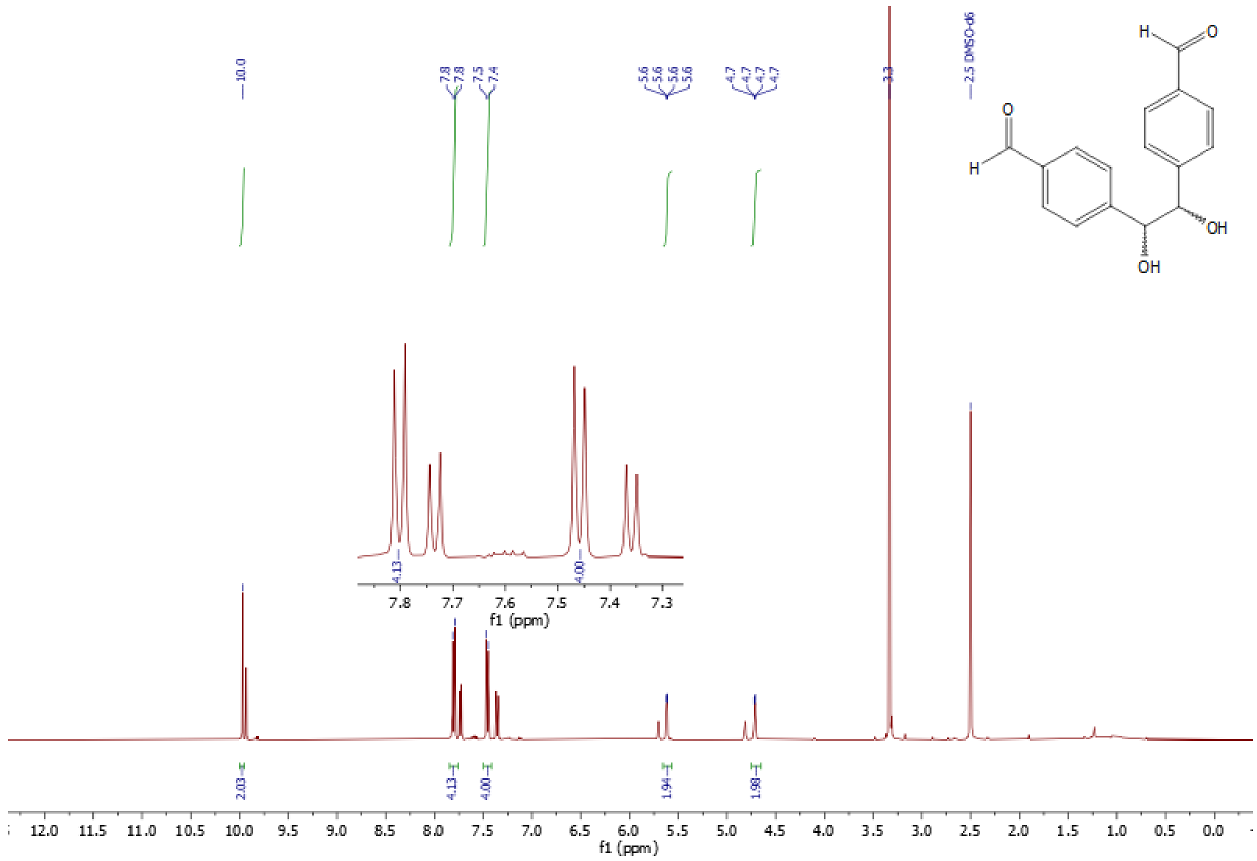

Figure S51. ^{1}H NMR spectrum of **16c** in DMSO-d_6.

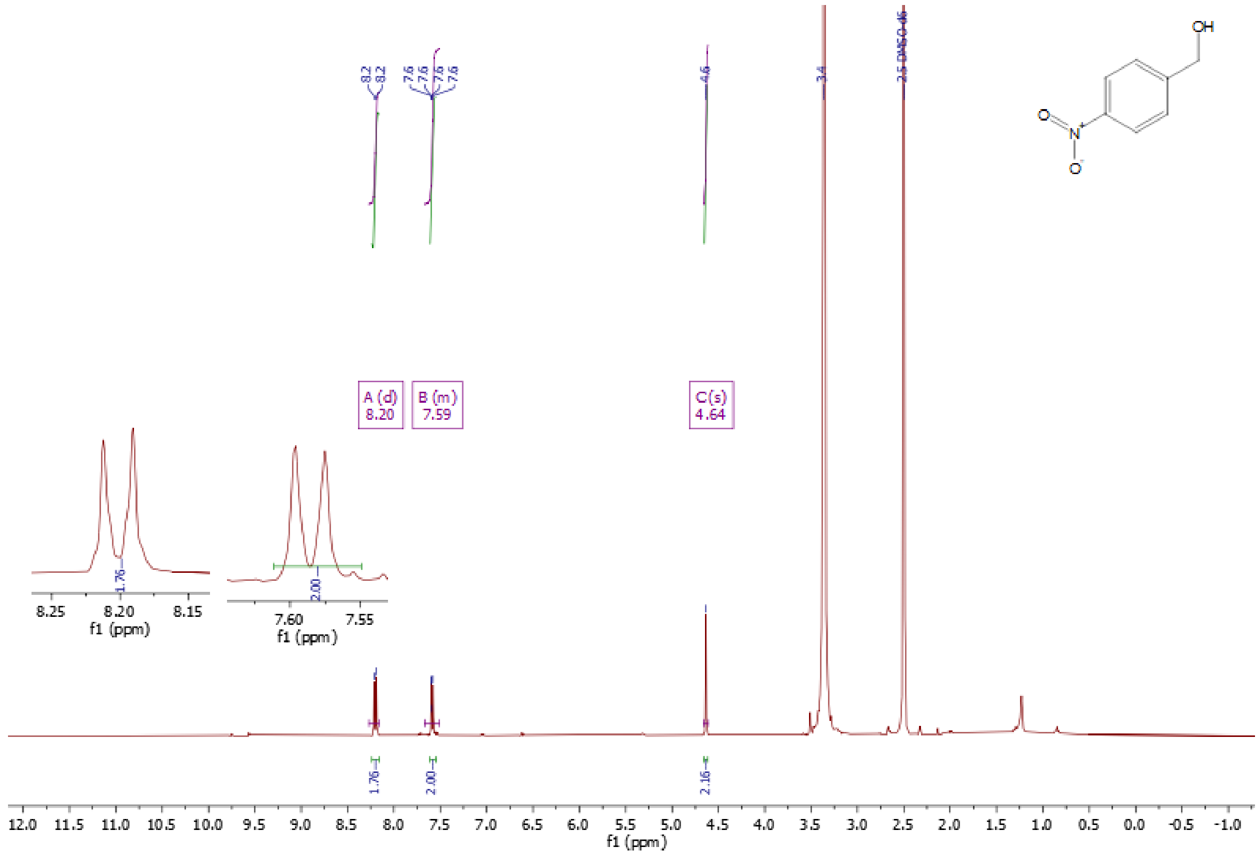

Figure S52. ^{1}H NMR spectrum of **17b** in DMSO-d_6.

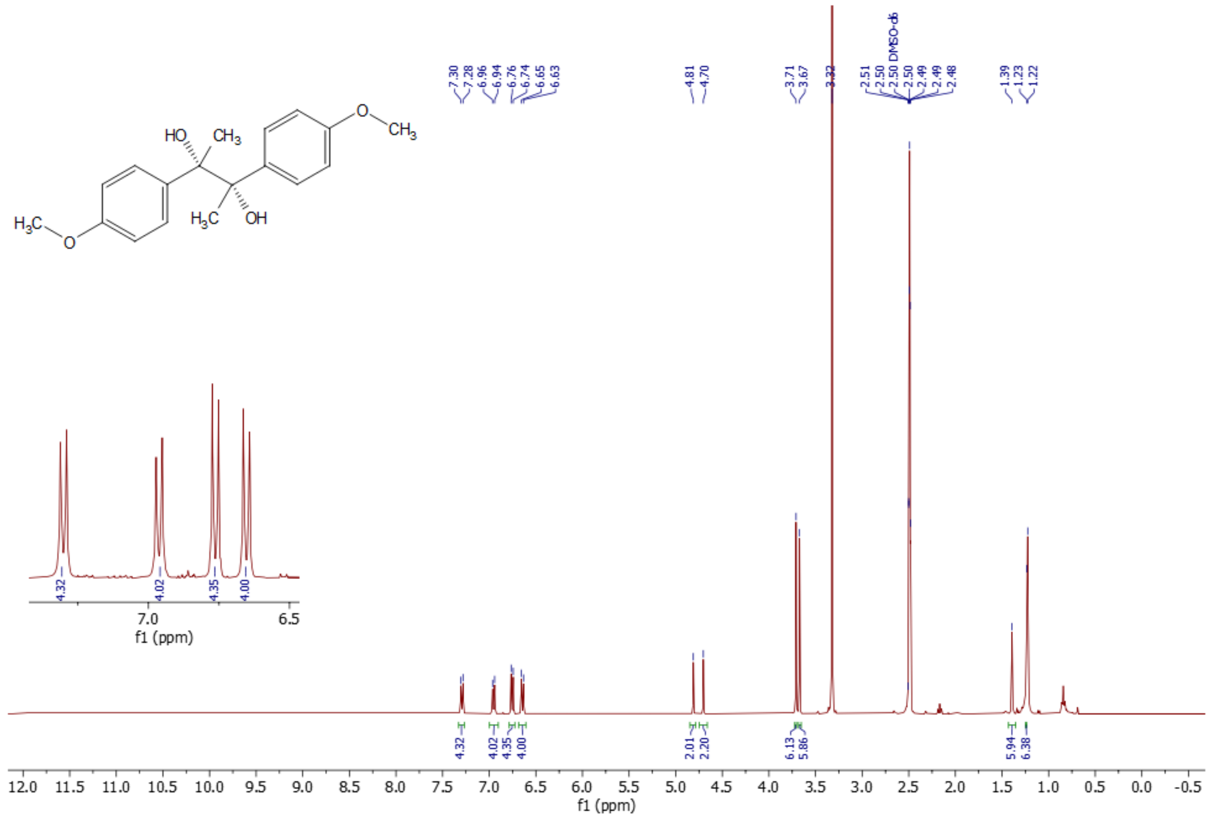

Figure S53. ^{1}H NMR spectrum of **18c** in DMSO-d_6.

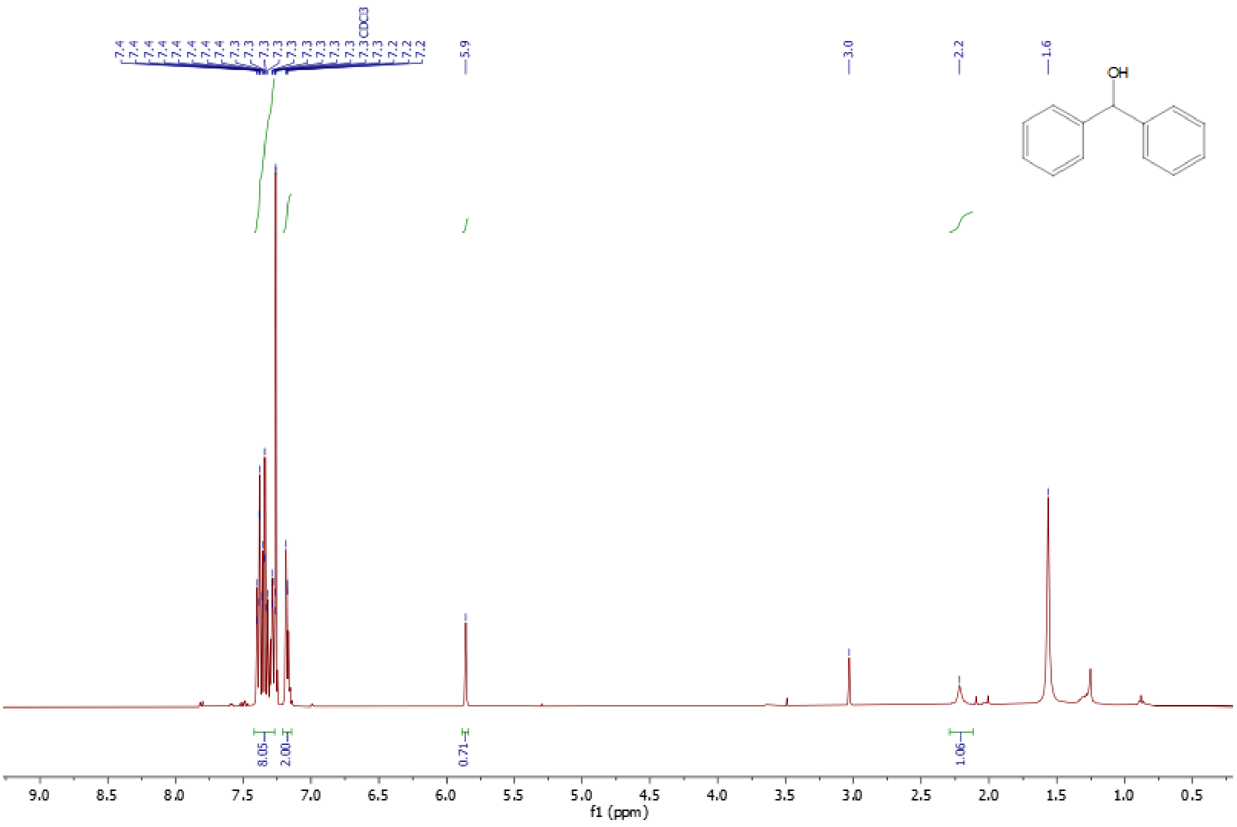

Figure S54. ^{1}H NMR spectrum of **19b** in $CDCl_3$.

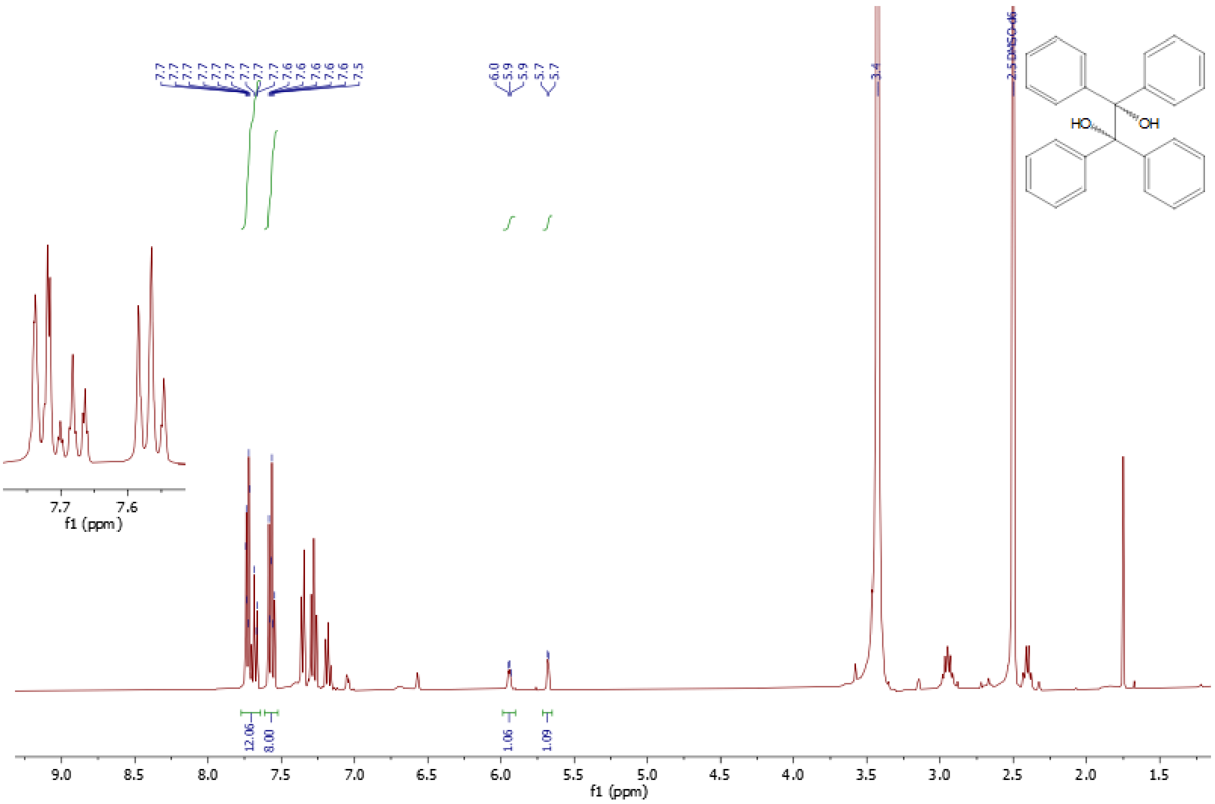

Figure S55. ^{1}H NMR spectrum of **19c** in DMSO-d_6.

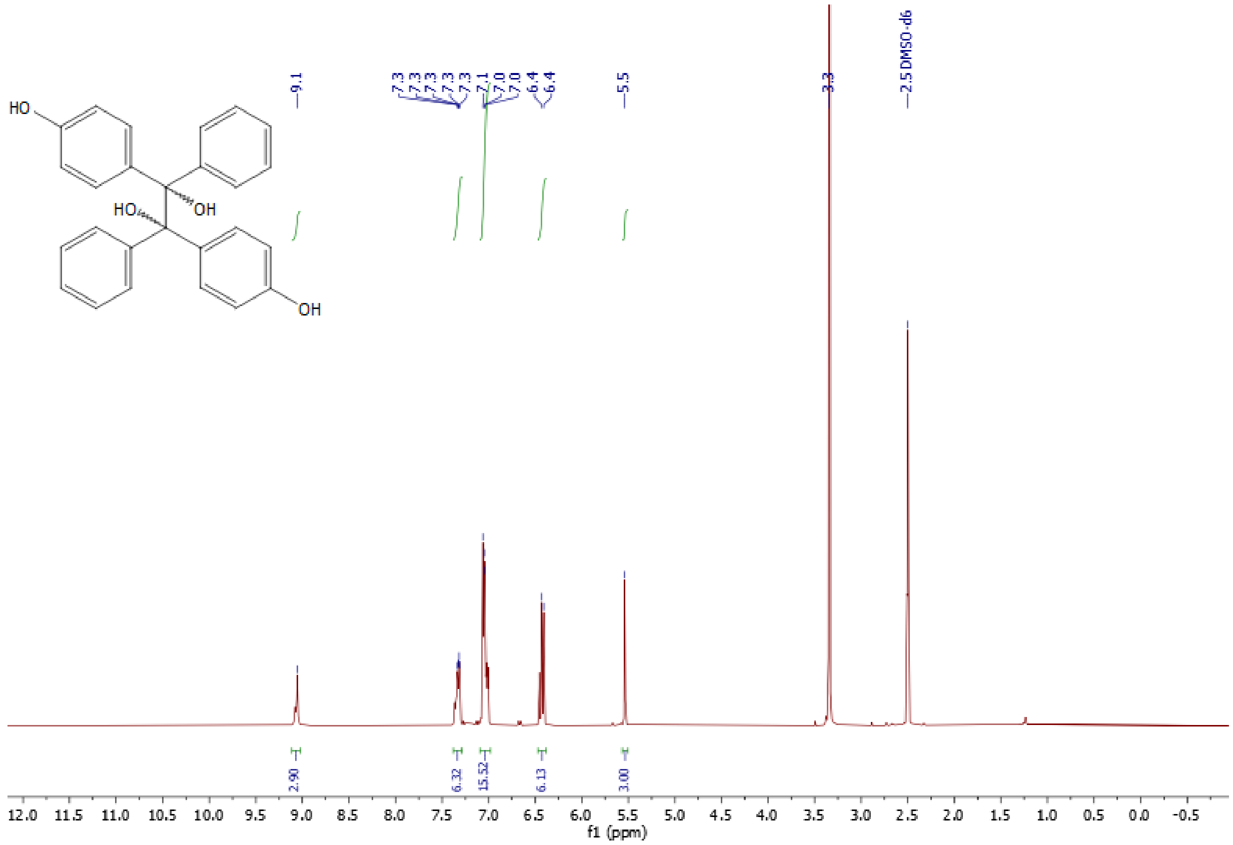

Figure S56. ^{1}H NMR spectrum of **20c** in DMSO-d_6.

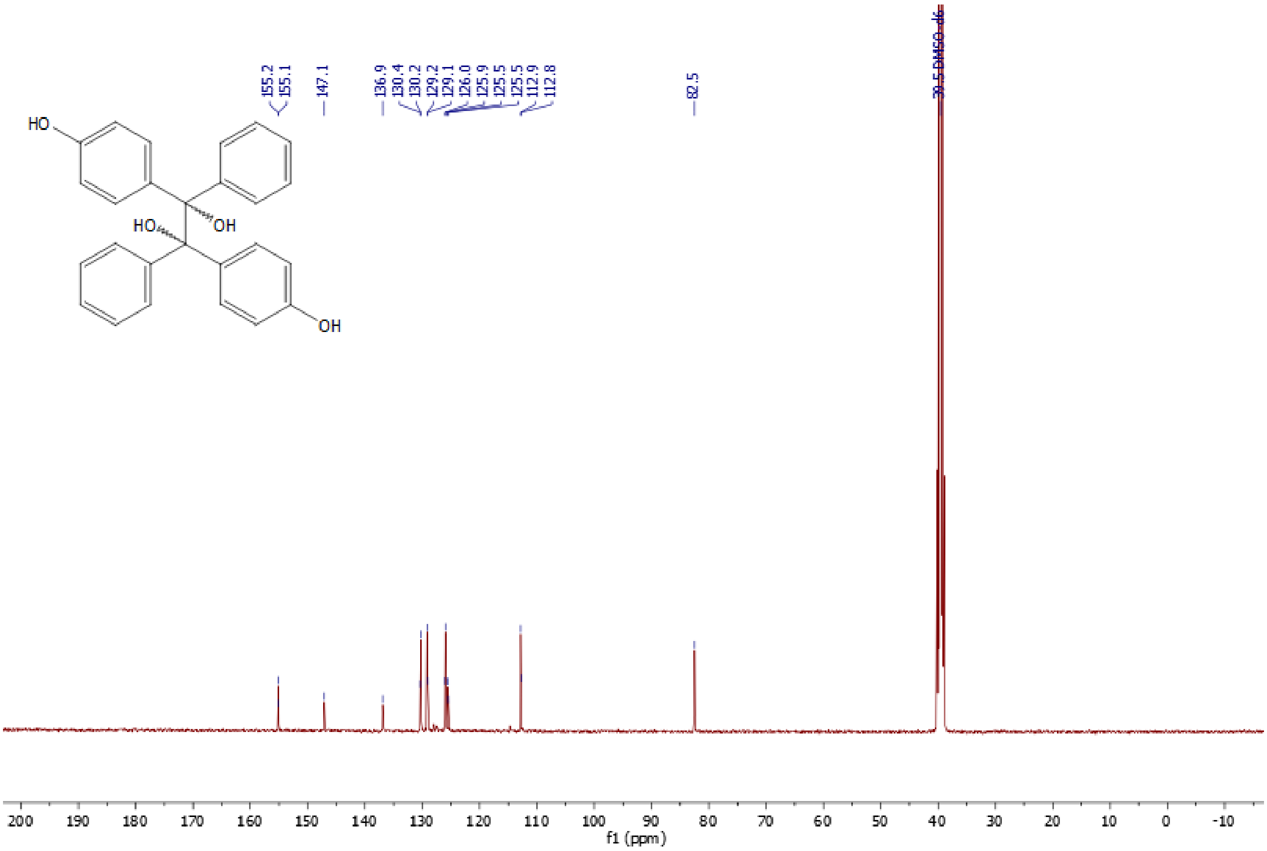

Figure S57. ^{13}C NMR spectrum of **20c** in DMSO-d_6.

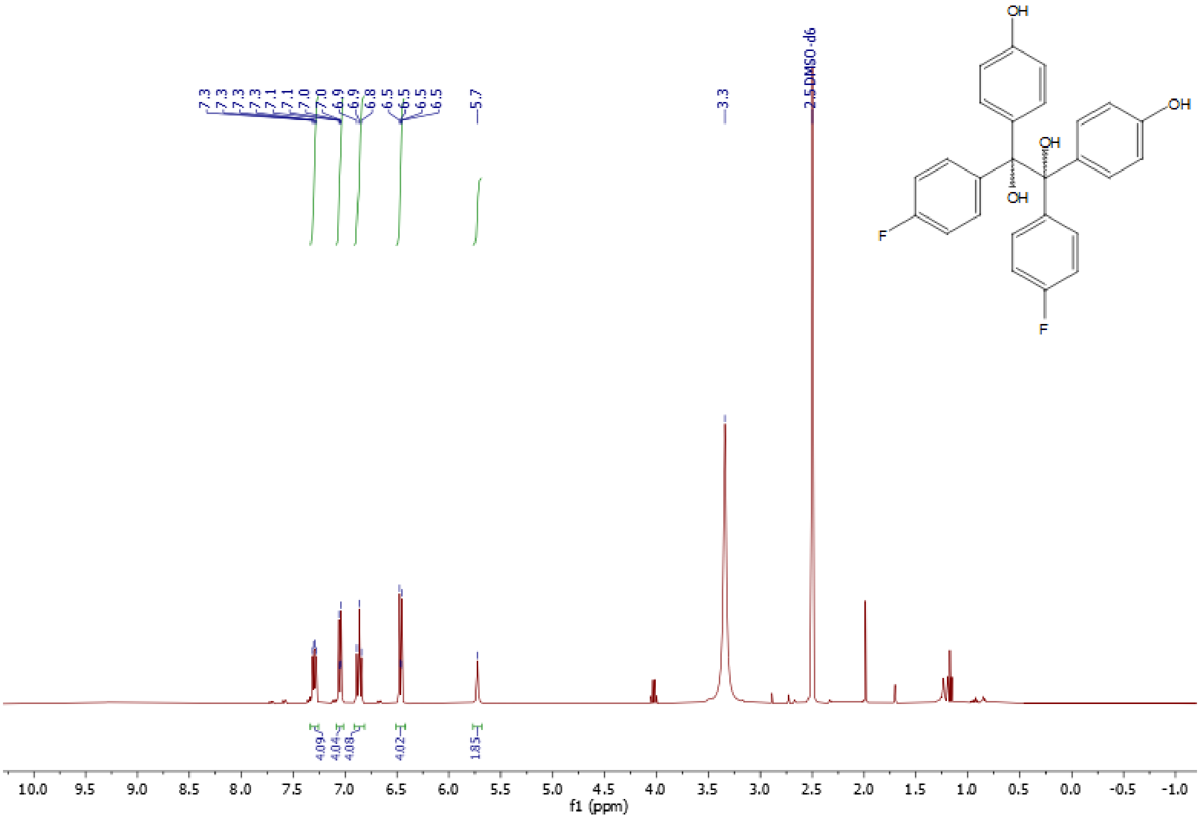

Figure S58. ^{1}H NMR spectrum of **21c** in DMSO-d_6.

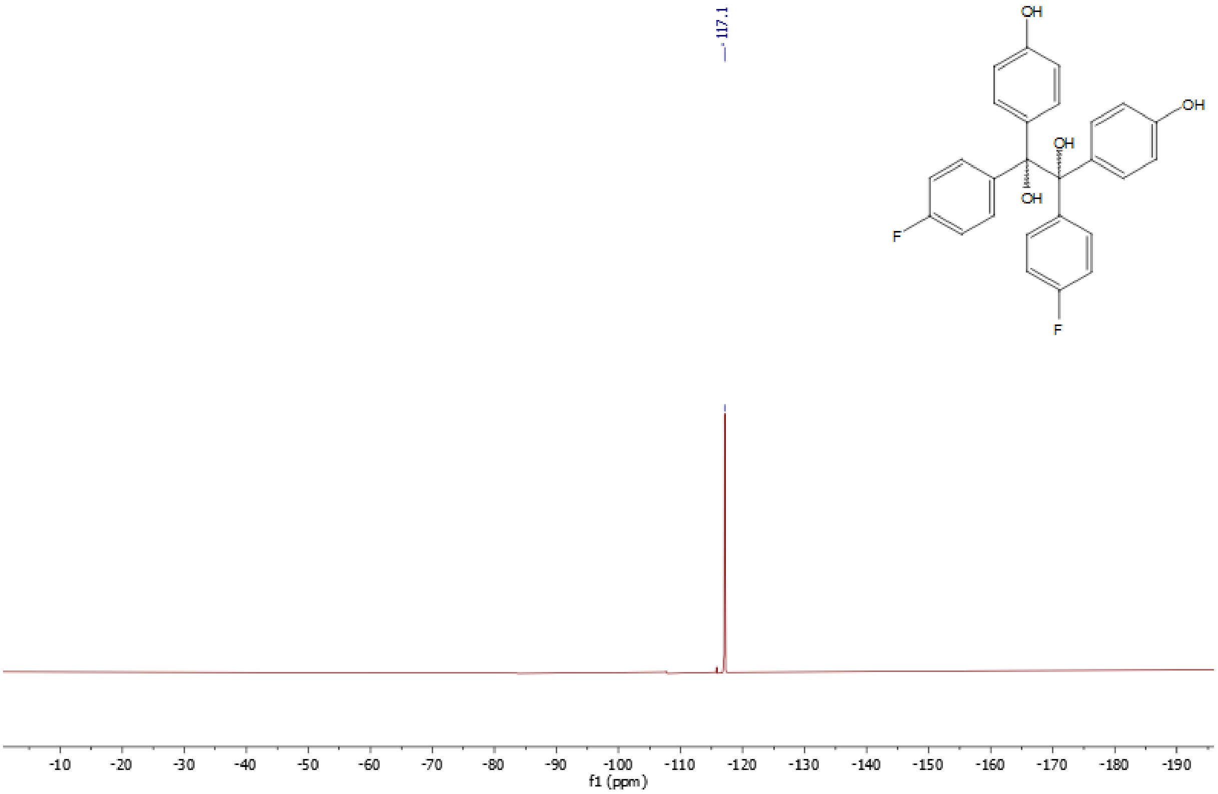

Figure S59. ^{19}F NMR spectrum of **21c** in DMSO-d_6.

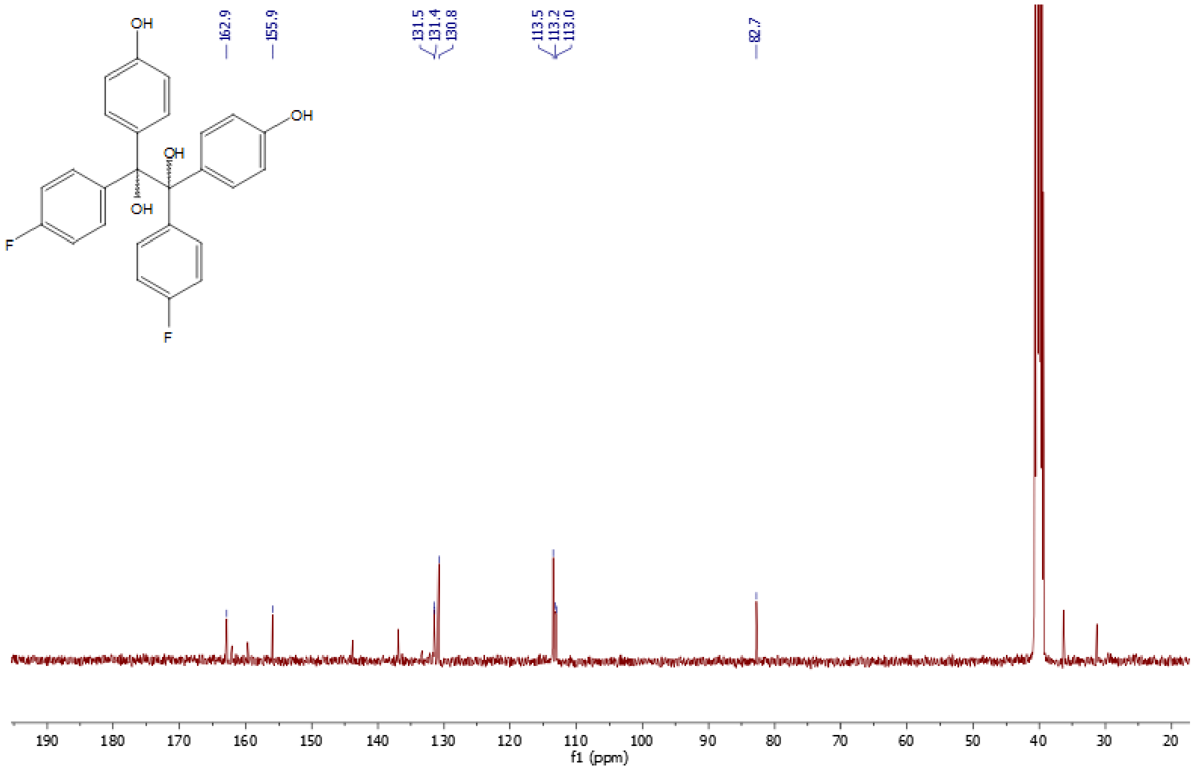

Figure S60. ^{13}C NMR spectrum of **21c** in DMSO-d_6.

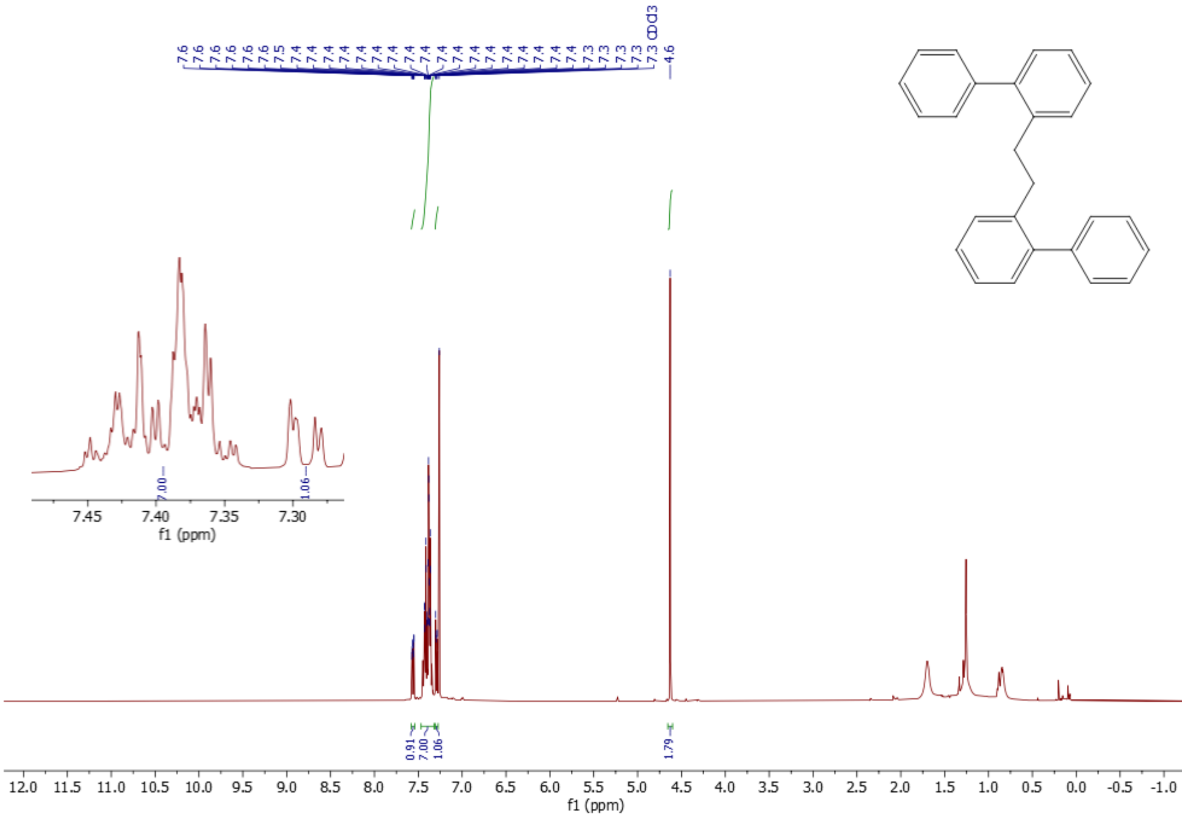

Figure S61. ^{1}H NMR spectrum of **22b** in $CDCl_3$.

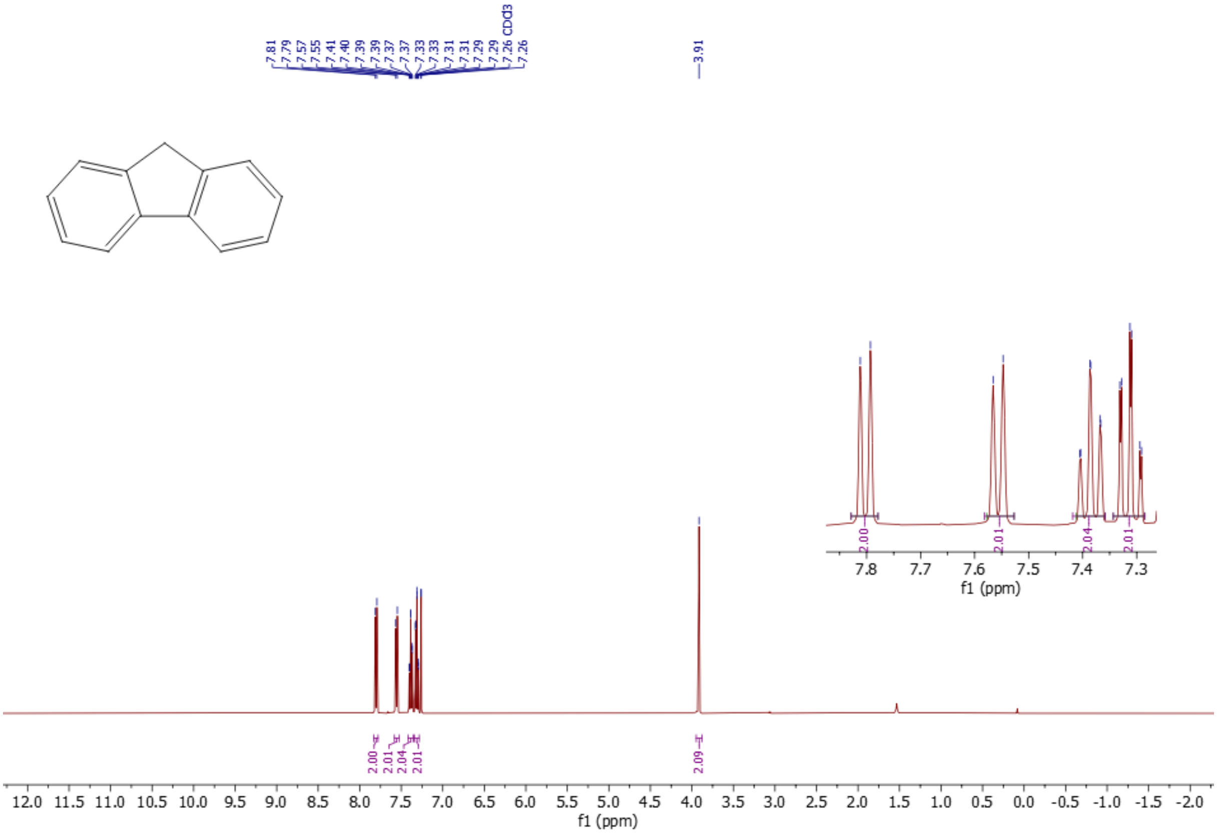

Figure S62. ^{1}H NMR spectrum of **22c** in $CDCl_3$.

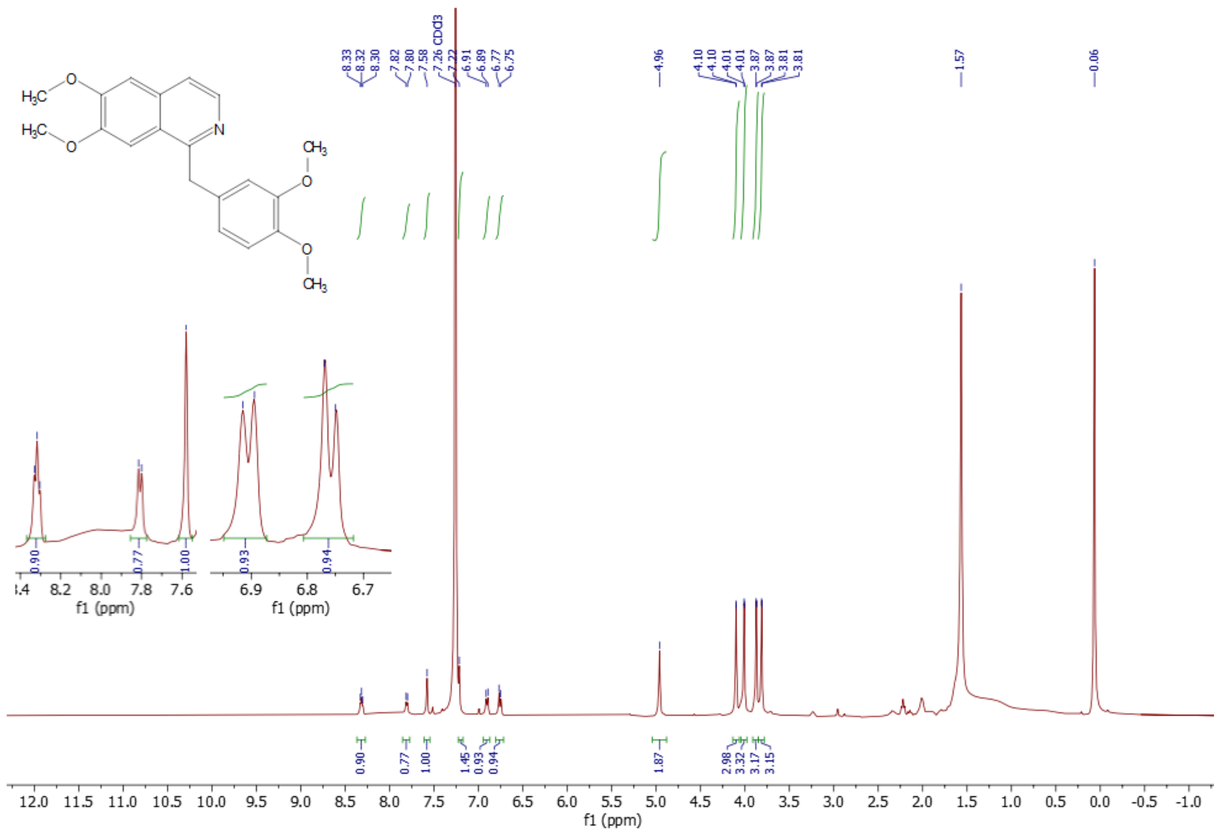

Figure S63. ^{1}H NMR spectrum of **23b** in $CDCl_3$.

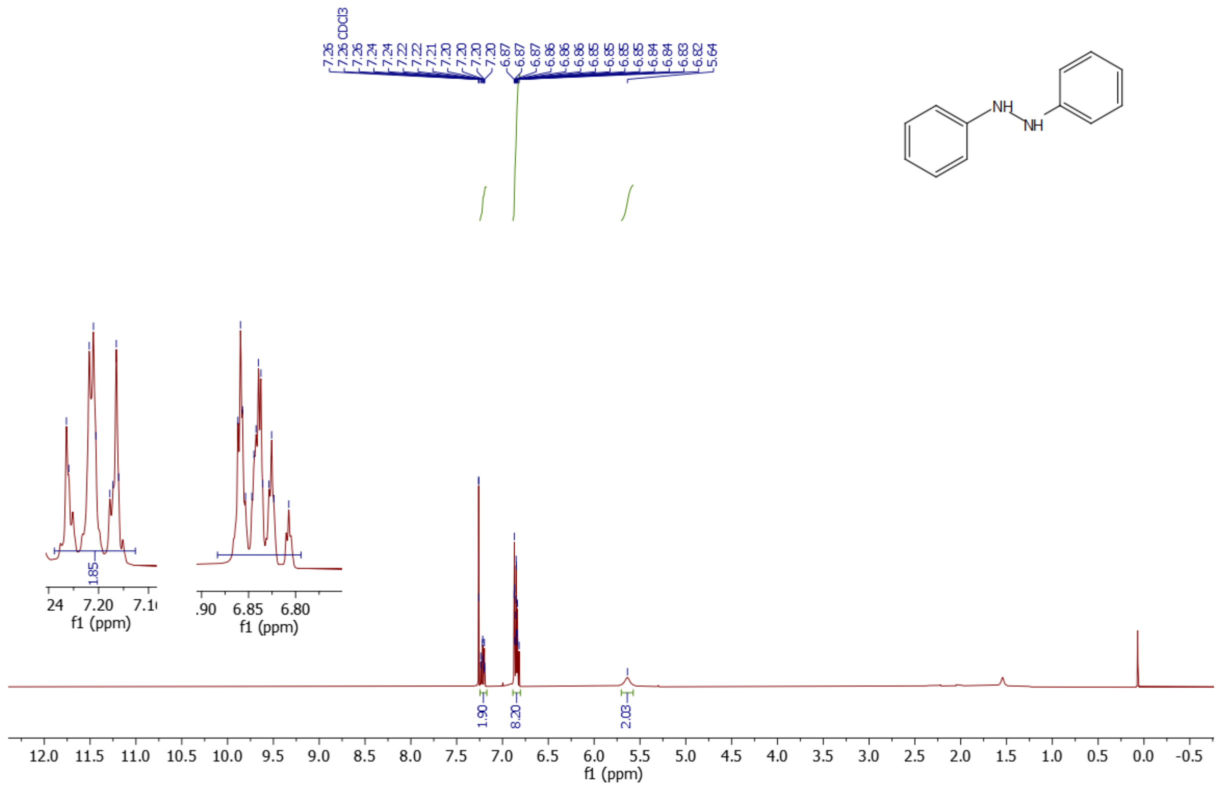

Figure S64. ^{1}H NMR spectrum of **24b** in $CDCl_3$.

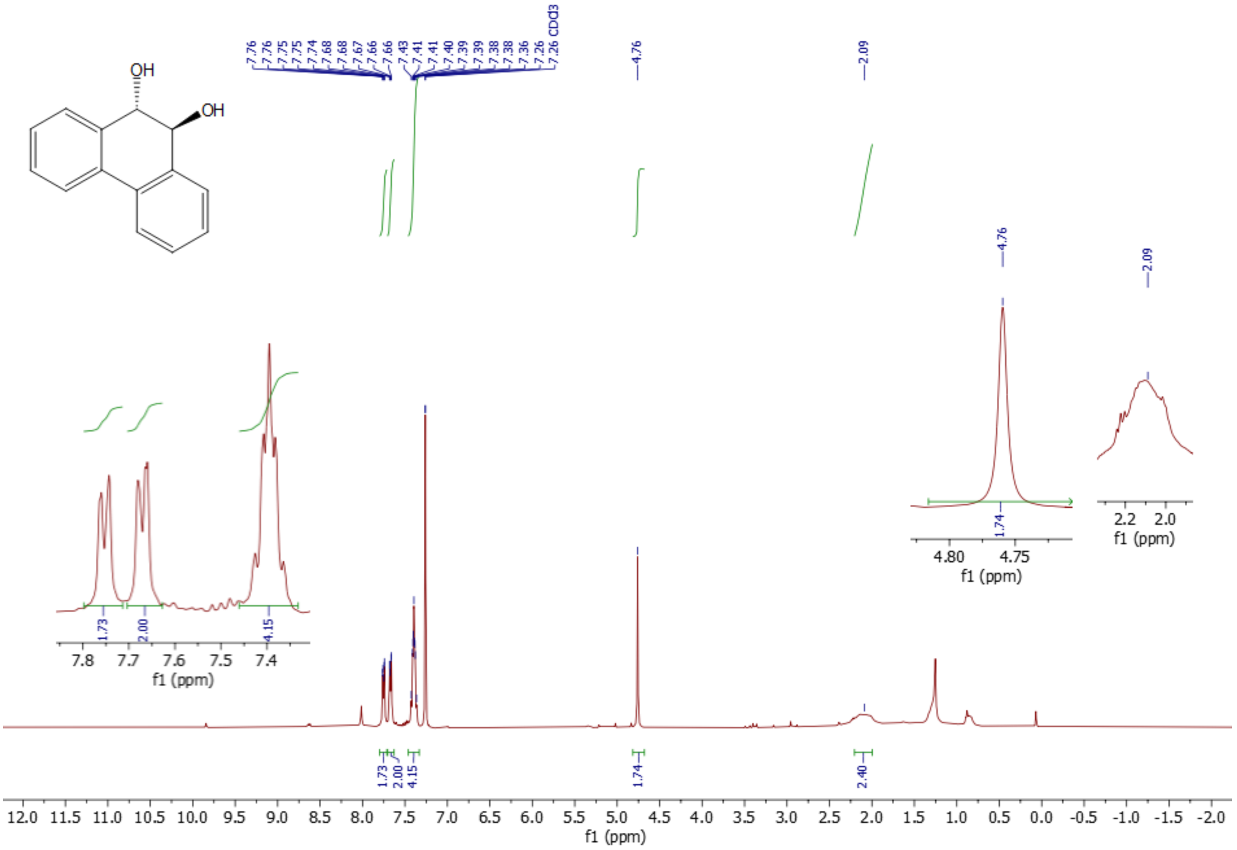

Figure S65. ^{1}H NMR spectrum of **25b** in $CDCl_3$.

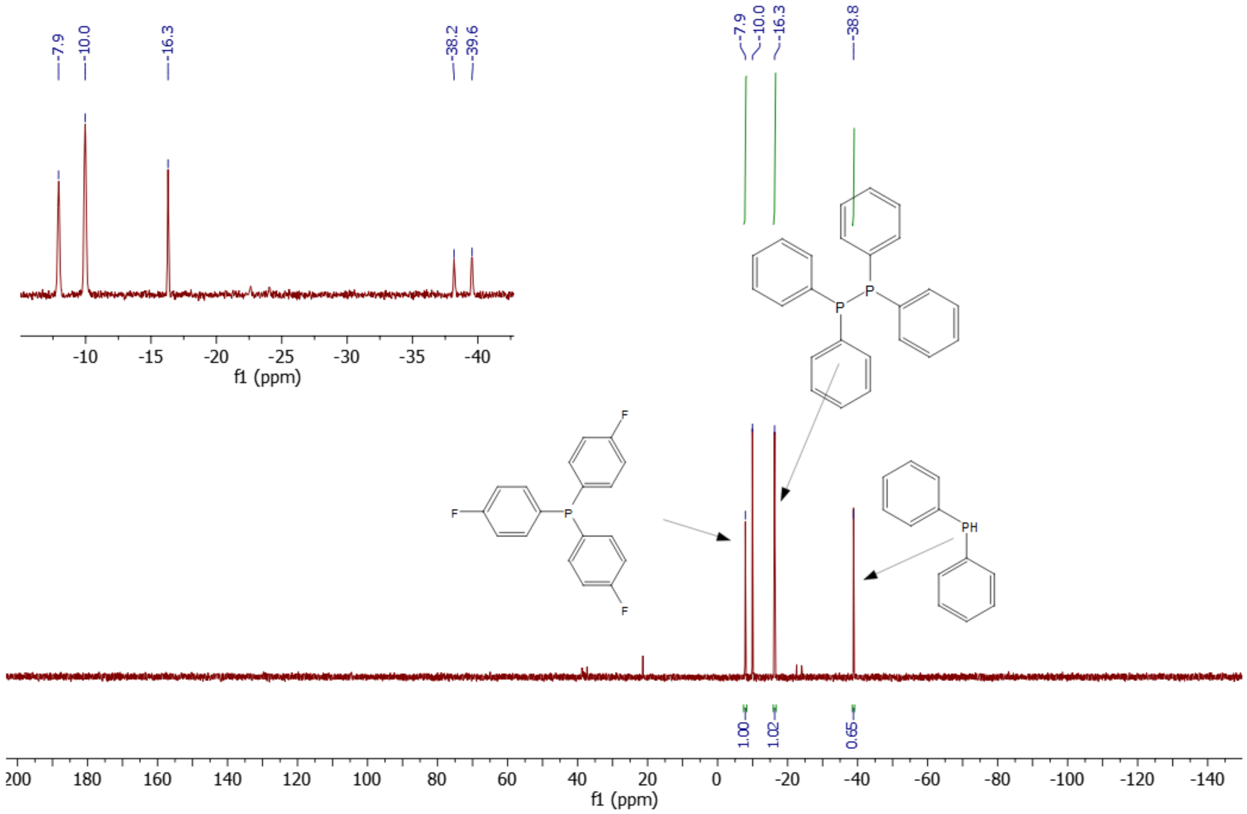

Figure S66. ^{31}P NMR spectrum of **26b** and **26c** in MeCN, tris(4-fluorophenyl) phosphine is used as an internal standard to determine reaction yields.

10. HR-MS data for new compounds

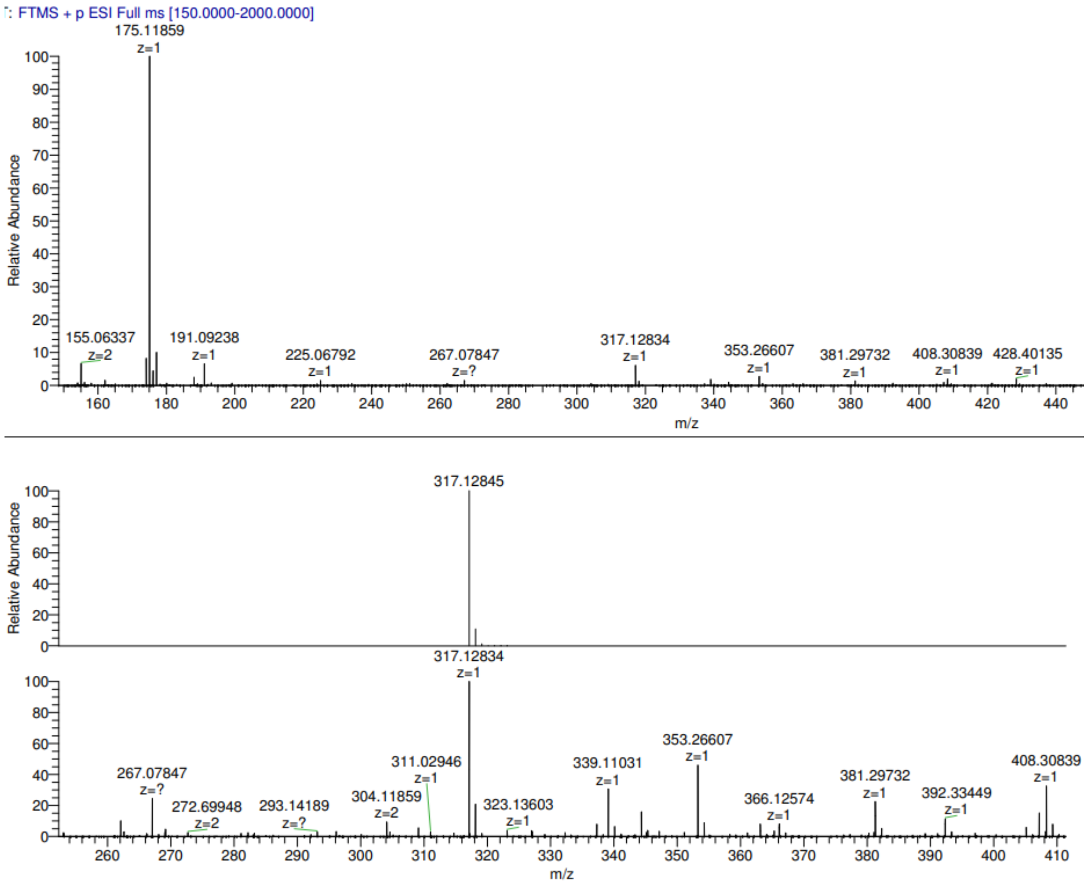

Figure S67. HR-MS of **3c**.

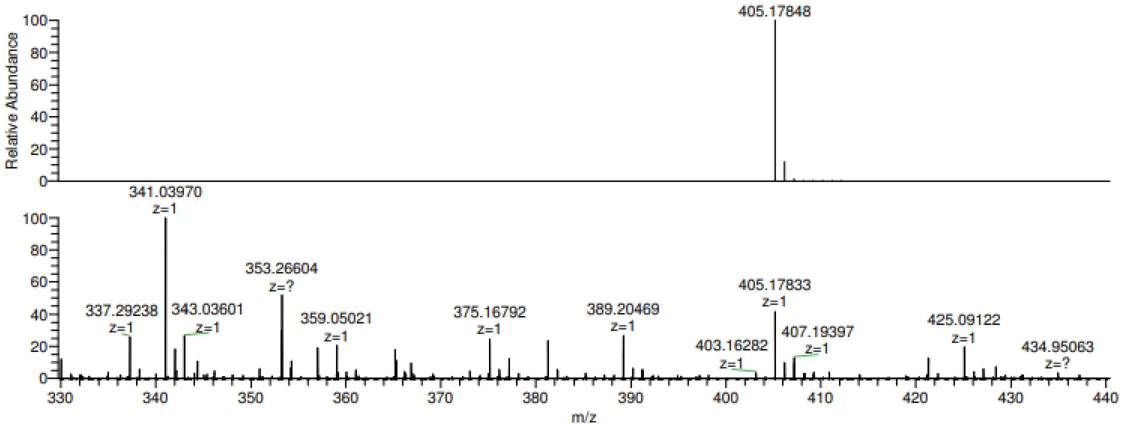

Figure S68. HR-MS of **6c**.

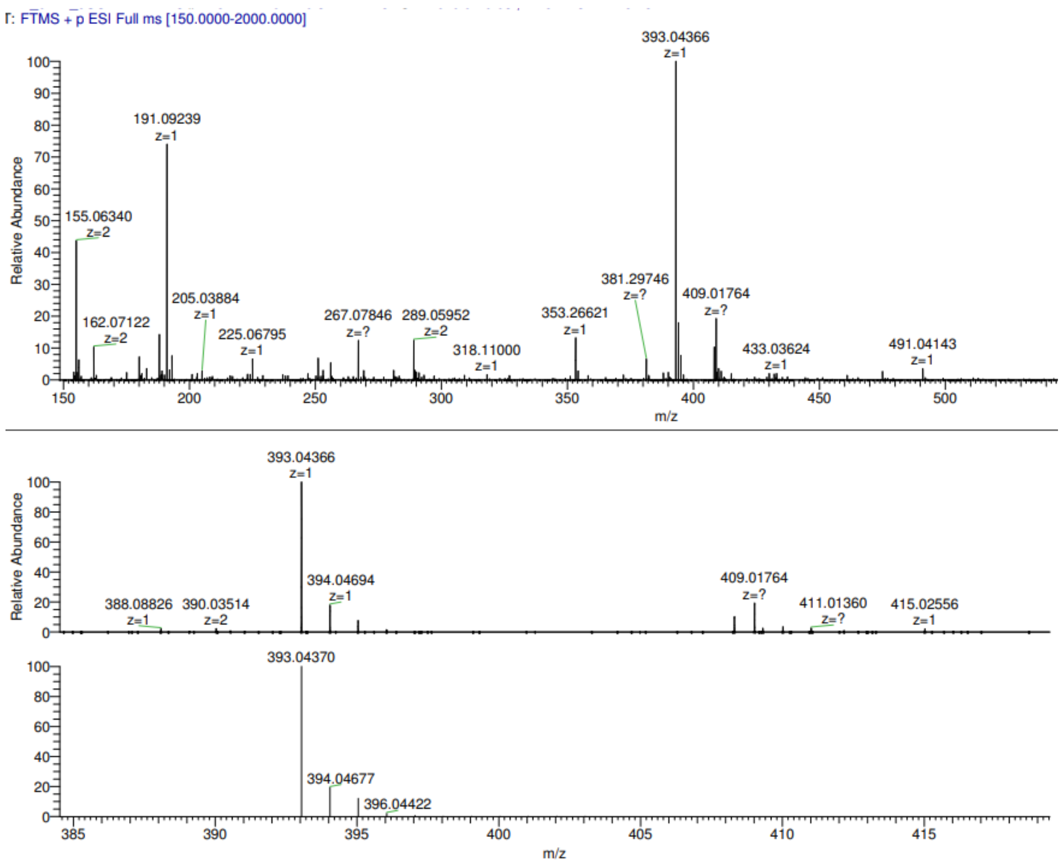

Figure S69. HR-MS of **12c**.

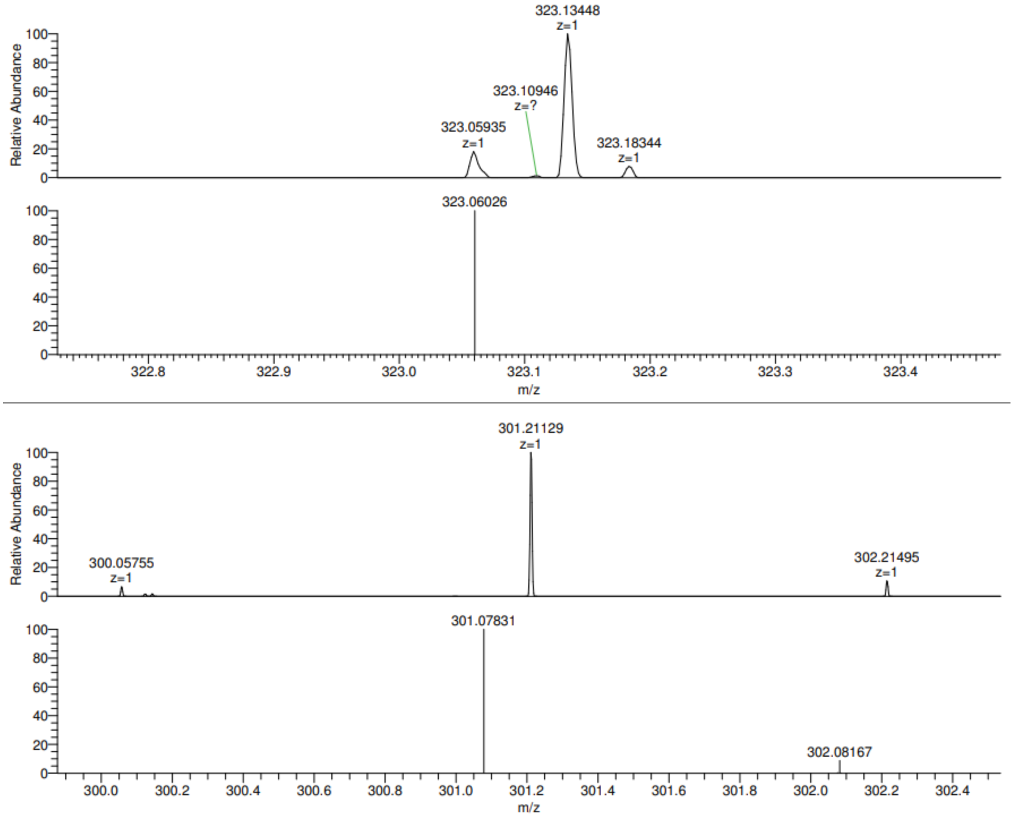

Figure S70. HR-MS of **13c**.

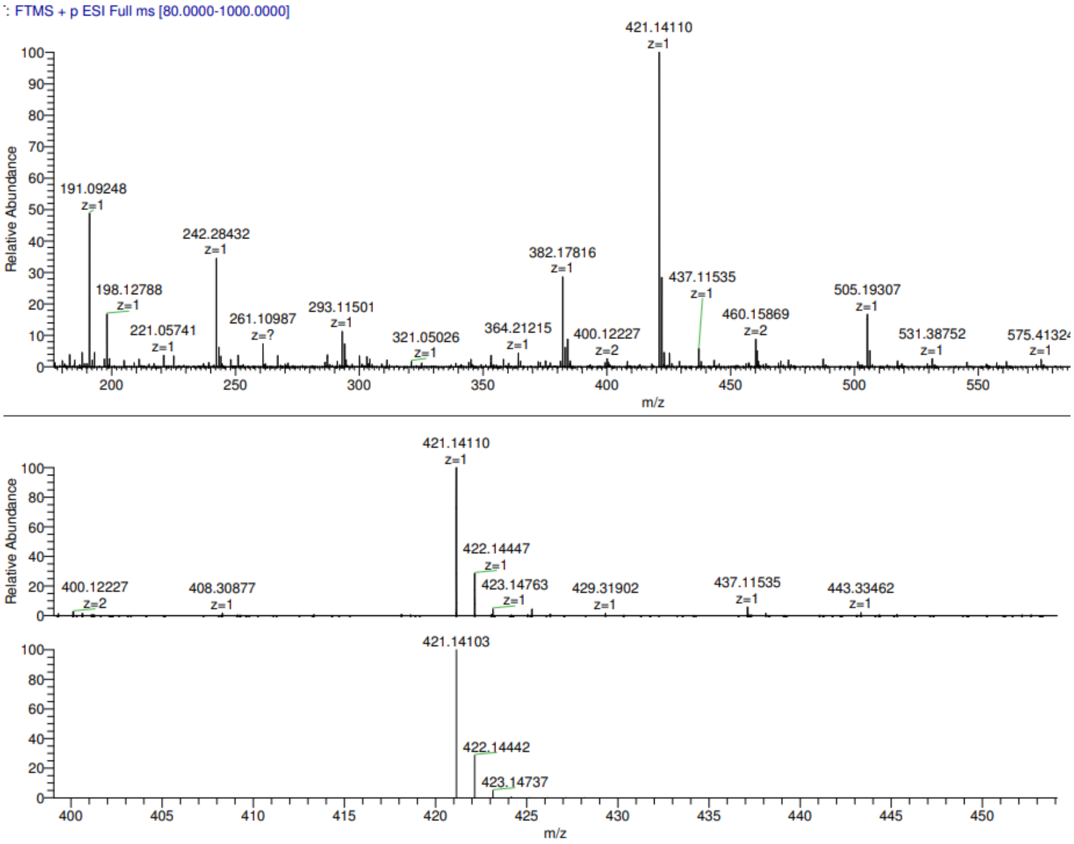

Figure S71. HR-MS of **20c**.

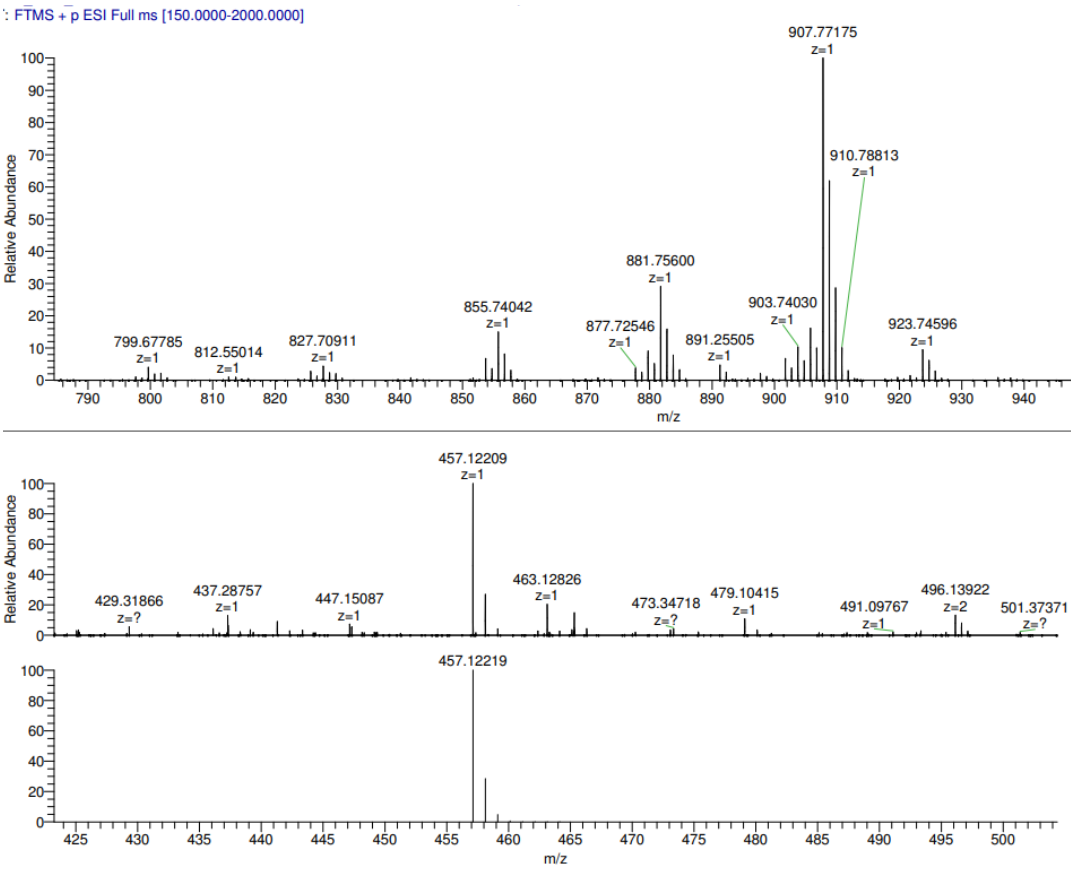

Figure S72. HR-MS of **21c**.

11. References

(1) Mutra, M. R.; Li, J.; Wang, J.-J. Light-mediated sulfonyl-iodination of ynamides and internal alkynes. *Chem. Commun.* **2023**, *59*(43), 6584-6587.
(2) Varma Nallaparaju, J.; Nikonovich, T.; Jarg, T.; Merzhyievskyi, D.; Aav, R.; Kananovich, D. G. Mechanochemistry-Amended Barbier Reaction as an Expedient Alternative to Grignard Synthesis. *Angew. Chem. Int. Ed.* **2023**, *62*(39), e202305775.
(3) Kim, Y. J.; Kim, S. M.; Hosono, H.; Yang, J. W.; Kim, S. W. The scalable pinacol coupling reaction utilizing the inorganic electride $[Ca_2N]^+ \cdot e^-$ as an electron donor. *Chem. Commun.* **2014**, *50*(37), 4791-4794.
(4) Li, L.; Kuo, H.-T.; Wang, X.; Merkens, H.; Colpo, N.; Radchenko, V.; Schaffer, P.; Lin, K.-S.; Bénard, F.; Orvig, C. ^{t}Bu$_4$octapa-alkyl-NHS for metalloradiopeptide preparation. *Dalton Trans.* **2020**, *49*(22), 7605-7619.
(5) Yan, Y.; Li, G.; Ma, J.; Wang, C.; Xiao, J.; Xue, D. Photoinduced generation of ketyl radicals and application in C–C coupling withoutexternal photocatalyst. *Green Chem.* **2023**, *25*(10), 4129-4136.
(6) Duan, X.-F.; Feng, J.-X.; Zi, G.-F.; Zhang, Z.-B. A Convenient Synthesis of Unsymmetrical Pinacols by Coupling of Structurally Similar Aromatic Aldehydes Mediated by Low-Valent Titanium. *Synthesis* **2009**, *2009*(02), 277-282.
(7) Lan, F.; Liu, C.-S.; Zhou, C.; Huang, X.; Wu, J.-Y.; Zhang, X. Developing highly reducing conjugated porous polymer: a metal-free and recyclable approach with superior performance for pinacol C–C coupling under visible light. *J. Mat. Chem. A* **2022**, *10*(31), 16578-16584.
(8) Li, J.-T.; Bian, Y.-J.; Zang, H.-J.; Li, T.-s. Pinacol coupling of aromatic aldehydes and ketones using magnesium in aqueous ammonium chloride under ultrasound. *Synth. Commun.* **2002**, *32*(4), 547-551.
(9) Kundu, S.; Roy, L.; Maji, M. S. Development of Carbazole-Cored Organo-Photocatalyst for Visible Light-Driven Reductive Pinacol/Imino-Pinacol Coupling. *Org. Lett.* **2022**, *24*(49), 9001-9006.
(10) Xu, X.; Yan, L.; Wang, S.; Wang, P.; Yang, A. X.; Li, X.; Lu, H.; Cao, Z.-Y. Selective synthesis of sulfoxides and sulfones via controllable oxidation of sulfides with N-fluorobenzenesulfonimide. *Org. Biomol. Chem.* **2021**, *19*(40), 8691-8695.
(11) Sandford, C.; Fries, L. R.; Ball, T. E.; Minteer, S. D.; Sigman, M. S. Mechanistic Studies into the Oxidative Addition of Co(I) Complexes: Combining Electroanalytical Techniques with Parameterization. *J. Am. Chem. Soc.* **2019**, *141*(47), 18877-18889.
(12) Zhang, D.; Ren, P.; Liu, W.; Li, Y.; Salli, S.; Han, F.; Qiao, W.; Liu, Y.; Fan, Y.; Cui, Y.; et al. Photocatalytic Abstraction of Hydrogen Atoms from Water Using Hydroxylated Graphitic Carbon Nitride for Hydrogenative Coupling Reactions. *Angew. Chem. Int. Ed.* **2022**, *61*(24), e202204256.
(13) Clerici, A.; Greco, C.; Panzeri, W.; Pastori, N.; Punta, C.; Porta, O. Reductive Coupling of Aromatic Aldehydes Promoted by an Aqueous $TiCl_3$/^{t}BuOOH System in Alcoholic Cosolvents. *Eur. J. Org. Chem.* **2007**, *2007*(24), 4050-4055.
(14) A. Sahade, D.; Kawaji, T.; Sawada, T.; Mataka, S.; Thiemann, T.; Tsukinoki, T.; Tashiro, M. Aluminium-mediated Reductive Dimerization of Aromatic Dialdehydes. *J. Chem. Res.* **1999**, 210-211.
(15) Iyer, K. S.; Nelson, C.; Lipshutz, B. H. Facile, green, and functional group-tolerant reductions of carboxylic acids…in, or with, water. *Green Chem.* **2023**, *25*(7), 2663-2671.
(16) Tomar, M.; Bhimpuria, R.; Kocsi, D.; Thapper, A.; Borbas, K. E. Photocatalytic Generation of Divalent Lanthanide Reducing Agents. *J. Am. Chem. Soc.* **2023**, *145*(41), 22555-22562.
(17) Shi, H.; Du, C.; Zhang, X.; Xie, F.; Wang, X.; Cui, S.; Peng, X.; Cheng, M.; Lin, B.; Liu, Y. Lewis Acid Assisted Electrophilic Fluorine-Catalyzed Pinacol Rearrangement of Hydrobenzoin Substrates: One-Pot Synthesis of (±)-Latifine and (±)-Cherylline. *J. Org. Chem.* **2018**, *83*(3), 1312-1319.
(18) Laha, J. K.; Gulati, U.; Saima. Effect of ortho-substitution on persulfate-mediated decarboxylation and functionalization of arylacetic acids. *New J. Chem.* **2023**, *47*(32), 15137-15142.
(19) Morimoto, K.; Itoh, M.; Hirano, K.; Satoh, T.; Shibata, Y.; Tanaka, K.; Miura, M. Synthesis of Fluorene Derivatives through Rhodium-Catalyzed Dehydrogenative Cyclization. *Angew. Chem. Int. Ed.* **2012**, *51*(22), 5359-5362.

(20) Chu, H.; Sun, S.; Yu, J.-T.; Cheng, J. Rh-catalyzed sequential oxidative C–H activation/annulation with geminal-substituted vinyl acetates to access isoquinolines. *Chem. Commun.* **2015**, *51*(68), 13327-13329.
(21) Arkhypchuk, A. I.; Tran, T. T.; Charaf, R.; Hammarström, L.; Ott, S. Mechanistic Insights and Synthetic Explorations of the Photoredox-Catalyzed Activation of Halophosphines. *Inorg. Chem.* **2023**, *62*(45), 18391-18398.
